# Design, Synthesis, Anti-Varicella-Zoster and Antimicrobial Activity of (Isoxazolidin-3-yl)Phosphonate Conjugates of N1-Functionalised Quinazoline-2,4-Diones

**DOI:** 10.3390/molecules27196526

**Published:** 2022-10-02

**Authors:** Magdalena Łysakowska, Iwona E. Głowacka, Graciela Andrei, Dominique Schols, Robert Snoeck, Paweł Lisiecki, Magdalena Szemraj, Dorota G. Piotrowska

**Affiliations:** 1 Bioorganic Chemistry Laboratory, Faculty of Pharmacy, Medical University of Lodz, Muszynskiego 1, 90-151 Lodz, Poland; 2 Laboratory of Virology and Chemotherapy, Rega Institute, Department of Microbiology, Immunology and Transplantation, KU Leuven, B-3000 Leuven, Belgium; 3 Department of Pharmaceutical Microbiology and Microbiological Diagnostics, Faculty of Pharmacy, Medical University of Lodz, Muszynskiego 1, 90-151 Lodz, Poland

**Keywords:** isoxazolidines, quinazoline-2,4-diones, phosphonates, antiviral activity, antimicrobial activity

## Abstract

Dipolar cycloaddition of the N-substituted *C*-(diethoxyphosphonyl)nitrones with *N*^3^-allyl-*N*^1^-benzylquinazoline-2,4-diones produced mixtures of diastereoisomeric 3-(diethoxyphosphonyl)isoxazolidines with a *N*^1^-benzylquinazoline-2,4-dione unit at C5. The obtained compounds were assessed for antiviral and antibacterial activities. Several compounds showed moderate inhibitory activities against VZV with EC_50_ values in the range of 12.63–58.48 µM. A mixture of isoxazolidines *cis*-**20c**/*trans*-**20c** (6:94) was found to be the most active against *B. cereus* PCM 1948, showing an MIC value 0.625 mg/mL, and also was not mutagenic up to this concentration.

## 1. Introduction

**Figure 1:**
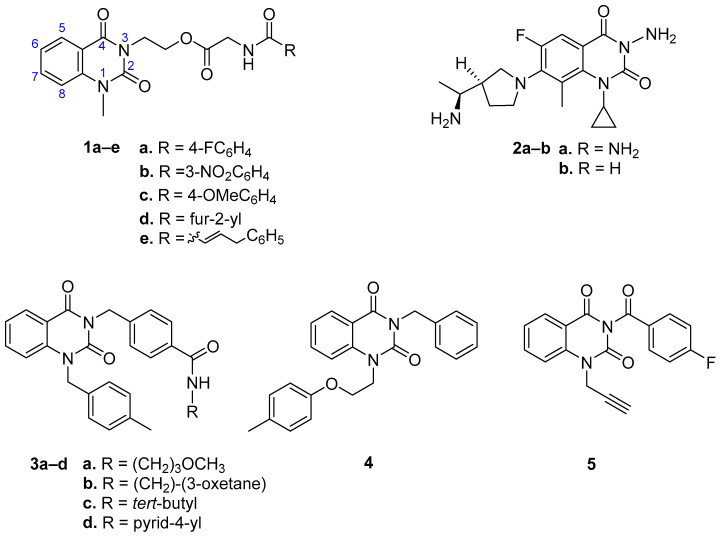
Examples of quinazoline-2,4-dione derivatives showing antibacterial and antiviral activity.

Quinazoline-2,4-diones belong to an important class of nitrogen-containing heterocyclic compounds with a wide spectrum of biological activities, including anticancer [1,2,3,4,5], antihypertensive [6], hypoglycemic [7] and anticonvulsant [8] activities, among others. Considerable attention has been focused on studies of antimicrobial and antiviral activities of 1,3-substituted quinazoline-2,4-diones, as some of them have promising biological activity (Figure 1). For example, *N*^1^-methylquinazoline-2,4-diones **1a–e** exhibited high antibacterial properties toward MRSA and *B. subtilis* and were slightly active against *S. aureus* [9], whereas compounds **2a–b** appeared to be suitable gyrase inhibitors that are active toward multidrug-resistance Gram-positive bacterial strains [10]. On the other hand, substituted quinazoline-2,4-dione derivatives **3a–d** displayed potent inhibitory activity against respiratory syncytial virus (RSV) (EC_50_ = 0.7–2.2 µM) [11], while compound **4** showed antiviral *activity against HIV-*1 and inhibited the recombinant RT in vitro [12], and *N*^1^-propargylquinazoline-2,4-dione **5** proved to be active towards adenovirus-2 (EC_50_ = 8.3 µM) [13].

**Figure 2:**
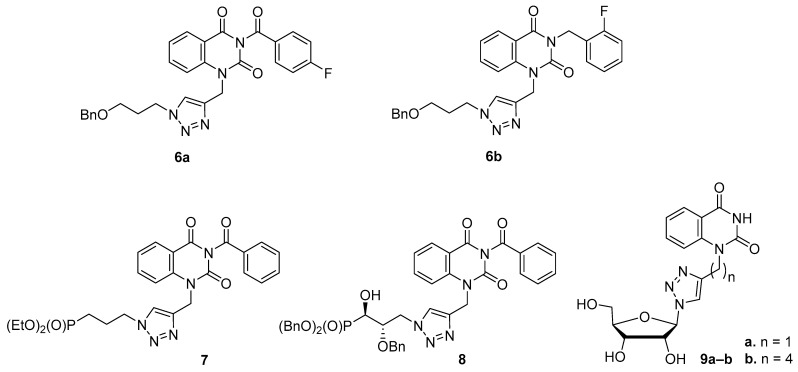
Examples of quinazoline-2,4-dione-containing nucleosides and nucleotides analogues.

Compounds with promising antiviral activity have been found as nucleoside and nucleotide analogues (Figure 2). For instance, 1,2,3-triazoles **6a–b** [13] and their phosphonylated analogues **7–8** [1,14] functionalized with quinazoline-2,4-dione moiety, both designed as acyclic nucleoside analogues, appeared to be active against herpes simplex viruses (HSV-1 and HSV-2) (EC_50_ values in the range of 2.9–17 µM, 4–11 µM towards HSV-1 and HSV-2, respectively) [1,13,14]. Additionally, compound **6a** inhibited the replication of varicella-zoster virus (VZV) at EC_50_ = 6.8–8.2, while derivatives **7** and **8** were also active toward feline herpes viruses (EC_50_ = 4, 24 µM for **7** and **8**, respectively) [1,13,14]. Quinazoline-2,4-dione-containing nucleosides **9a–b** showed antiviral activity against influenza virus (H1N1) (IC_50_ = 30–42 µM) [15].

**Figure 3:**
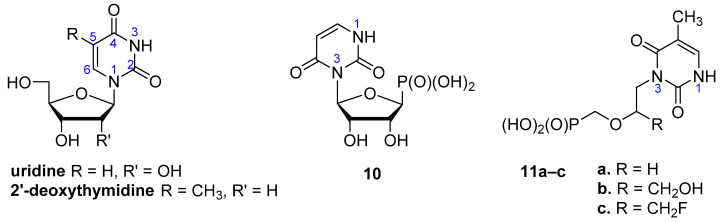
Examples of natural nucleosides (uridine, 2′-deoxythymidine) and N3-substituted pyrimidine nucleotide analogues **10–11**.

In the vast majority of natural pyrimidine nucleosides, e.g., uridine and 2′-deoxythymidine and their synthetic analogues, furanose ring or its mimetic is linked to the N1 of a pyrimidine moiety; however, analogues with nucleobase attached to sugar via the N3 atom of pyrimidine have been also obtained. Among them, nucleotide analogues **10** and **11** (Figure 3) show even higher antiviral activity than the respective N1-isomeric nucleotides [16,17].

**Scheme 1:**
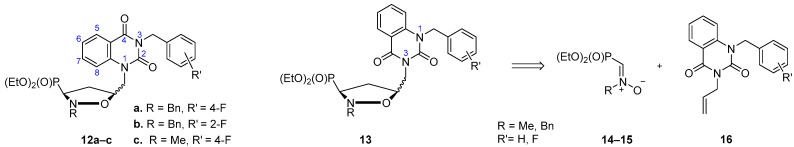
The general structure of compound **12** and retrosynthesis of newly designed quinazoline-2,4-dione-conjugates of isoxazolidine **13**.

Recently, we achieved the synthesis of isoxazolidine nucleotide analogues **12a–c** with functionalized quinazoline-2,4-dione as a nucleobase replacer, and their promising anti-varicella-zoster virus (VZV) activity has been recognized (EC_50_ = 3.0–5.1 µM) [18]. Based on the observed biological properties of various quinazoline-2,4-dione derivatives, such as **12,** and in continuation of our studies on isoxazolidine analogues of nucleosides, a new series of compounds of the general formula **13** with quinazoline-2,4-dione linked via the N3 atom to the isoxazolidine moiety was synthesized to evaluate the biological activity. The route to construct the designed compound **13** relies on the 1,3-dipolar cycloaddition of N-substituted *C*-(diethoxyphosphonyl)nitrones **14–15** [19] with selected N3-allylated quinazoline-2,4-dione **16** functionalized at N3 with the respective benzyl group (Scheme 1).

## 2. Results and Discussion

### 2.1. Chemistry

**Scheme 2:**
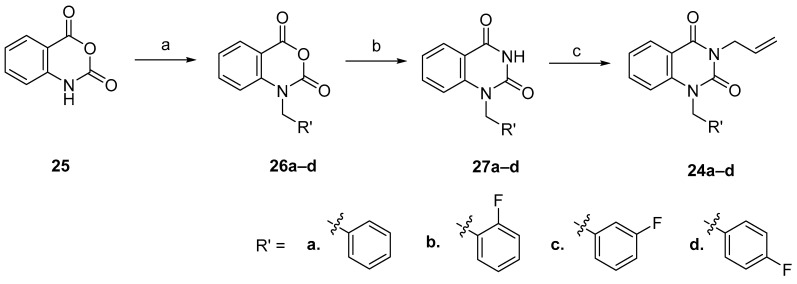
Synthesis of *N*^3^-allyl-*N*^1^-benzylquinazoline-2,4-diones **24a–d**. Reaction and conditions: (a) selected benzyl bromide, NaH, DMF, r.t., 18 h; (b) urea, DMF, reflux, 5 h; (c) allyl bromide, MeCN, 105 °C, 4 h.

The respective *N*^3^-allyl-*N*^1^-benzylquinazoline-2,4-diones **24a–d** were synthesized from the commercially available isatoic anhydride **25** (Scheme 2). Benzylation of **25** with the selected benzyl bromide [20], followed by the reaction of the resulted compounds **26a–d** with urea, led to the formation of the respective *N*^1^-benzylquinazoline-2,4-diones **27a–d**. Subsequent allylation of derivatives **27a–d** with allyl bromide produced compounds **24a–d**.

**Scheme 3:**
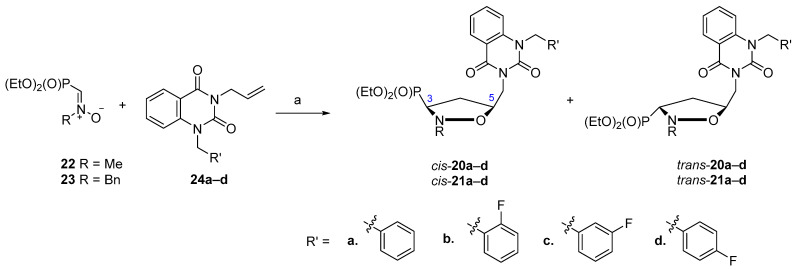
Synthesis of isoxazolidines **20** and **21**. Reaction and conditions: (a) toluene, 60 °C, 72 h.

The 1,3-dipolar cycloadditions of the respective nitrone **22** (R = Me) or **23** (R = Bn) with the selected *N*^3^-allyl-*N*^1^-benzylquinazoline-2,4-diones **24a–d** were carried out at 60 °C in toluene. In all cases, the regiospecific formation of the diastereoisomeric mixtures of 3,5-disubstituted isoxazolidines *trans*-**20** and *cis*-**20** or *trans*-**21** and *cis*-**21** was observed, with the *trans*-isomer predominating (Scheme 3, Table 1). In the case of isoxazolidines **20a** and **20c** as well as **21a–d** *trans/cis* ratios, diastereoisomers were determined based on analyses of ^31^P NMR spectra of crude products, since two well-separated signals were observed. For diastereoisomeric pairs of isoxazolidines **20b** and **20d,** *trans/cis* ratios were calculated from ^1^H NMR spectra of crude reaction mixtures by comparison of diagnostic resonances of CH_3_–N protons in the isoxazolidine ring. The diastereoselectivity values (d.e.) ranged from 70 to 84%. The mixtures of the respective cycloadducts were subjected to purification on silica gel columns; however, attempts to isolate pure diastereoisomers were fruitless and, in each case, only mixtures of diastereoisomers isoxazolidines *trans*-**20** and *cis*-**20** or *trans*-**21** and *cis*-**21** were isolated.

The relative configurations of the isoxazolidine cycloadducts *trans*-**20** and *cis*-**20** or *trans*-**21** and *cis*-**21** were established by taking in account our previous studies on the stereochemistry of cycloaddition of N-substituted *C*-diethyoxyphosphonylated nitrones **22** and **23** with allylated derivatives of various (hetero)aromatic compounds [21,22], including N3-substituted N1-allylquinazoline-2,4-diones [18]. Since modification of the substituents in quinazoline-2,4-dione moiety, including the relocation of substituents at N1 and N3, has no influence on the stereochemical outcome of the cycloaddition of nitrones **22** and **23** to N-allylquinazoline-2,4-dione dipolarophiles, configurations of all major isoxazolidines **20** and **21** were assigned *trans*, while minor isomers were assigned *cis*, by analogy to previously established configurations of *trans*- and *cis*-isoxazolidines **12a–c** [18].

### 2.2. Antiviral and Antimicrobial Evaluation

#### 2.2.1. Antiviral Activity

All synthesized alkenes **24a–d** and the respective diastereoisomeric mixtures of isoxazolidines *cis*-**20a**/*trans*-**20a** (10:90), *cis*-**20b**/*trans*-**20b** (8:92), *cis*-**20c**/*trans*-**20c** (6:94), *cis*-**20d**/*trans*-**20d** (10:90), *cis*-**21a**/*trans*-**21a** (10:90), *cis*-**21b**/*trans*-**21b** (15:85), *cis*-**21c**/*trans*-**21c** (10:90), and *cis*-**21d**/*trans*-**21d** (15:85) were tested for inhibitory activity of a wide variety of DNA and RNA viruses, using the following cell-based assays: (a) human embryonic lung (HEL) cells: herpes simplex virus-1 (KOS), herpes simplex virus-2 (G), herpes simplex virus-1 (TK^–^ KOS ACV^r^), cytomegalovirus (AD-169 strain, Davis strain), varicella-zoster virus (TK^+^ VZV OKA strain and TK^–^ VZV 07-1 strain), vaccinia virus, adenovirus-2, human coronavirus (229E); (b) HeLa cell cultures: vesicular stomatitis virus, Coxsackie virus B4, respiratory syncytial virus; (c) Vero cell cultures: para-influenza-3 virus, reovirus-1, Sindbis virus, Coxsackie virus B4, Punta Toro virus, yellow fever virus; and (d) Madin–Darby canine kidney (MDCK) cell cultures: influenza A virus (H1N1 and H3N2 types), influenza B virus. Ganciclovir, cidofovir, acyclovir, brivudin, zalcitabine, alovudine, UDA, ribavirin, dextran sulfate (molecular weight 10000, DS.-10000), mycophenolic acid, zanamivir, amantadine, and rimantadine were used as the reference compounds. The antiviral activity was expressed as the EC_50_: the effective concentration required to reduce virus plaque formation (VZV, HCMV) by 50% or to reduce virus-induced cytopathogenicity by 50% (other viruses).

The synthesized compounds only showed some antiviral activity toward varicella-zoster virus, including both TK^+^ and TK^–^ VZV strains (Table 2). Among the series of tested compounds, isoxazolidines *cis*-**20a–d**/*trans*-**20a–d** and **21a–d**/*trans*-**21a–d** showed higher inhibitory activity against VZV than the respective alkenes **24a–d**. Isoxazolidiens *cis*-**21b/***trans*-**21b** (15:85) and cis-**21a**/trans-**21a** (10:90) exhibited the highest activity against TK^+^ VZV OKA strain (EC_50_ = 12.63 µM and EC_50_ = 14.5 µM, respectively); however, this antiviral activity was lower than that of reference drug acyclovir. Moreover, preliminary structure–activity relationship observations revealed the slightly higher activity of compounds with benzyl group at nitrogen in the isoxazolidine ring in comparison to their N-methyl analogues (*cis*-**21**/*trans*-**21** vs. analogous *cis*-**20**/*trans*-**20**).

#### 2.2.2. Antimicrobial Activity

Due to the increasing resistance of bacteria to antibiotics, new compounds with antibacterial properties are being searched as alternatives to antibiotics. For this reason, the biological screening was expanded to evaluate the antimicrobial activity of alkenes **24a–d** and diastereoisomeric mixtures of isoxazolidines (*cis*-**20a**/*trans*-**20a** (10:90), *cis*-**20b**/*trans*-**20b** (8:92), *cis*-**20c**/*trans*-**20c** (6:94), *cis*-**20d**/*trans*-**20d** (10:90), *cis*-**21a**/*trans*-**21a** (10:90), *cis*-**21b**/*trans*-**21b** (15:85), *cis*-**21c**/*trans*-**21c** (10:90), and *cis*-**21d**/*trans*-**21d** (15:85)) towards selected bacterial strains (*E. faecalis* ATCC 29212, *S. aureus* ATCC 2593, *B. cereus* PCM 1948, *E. coli* ATCC 25922, and *P. aeruginosa* ATCC 27853) and two fungal strains *(C. albicans* ATCC 10241 and *A. brasiliensis* ATCC 16404). Previous studies have shown that compounds containing a substituted quinazoline-2,4-dione moiety exhibit promising activity against Gram-negative and Gram-positive bacteria and fungi [9,23,24,25]. The antimicrobial activity was expressed as the MIC, minimal inhibitory concentrations, and the MBC, minimal bactericidal concentrations. Among all tested isoxazolidines, compounds *cis*-**20c/***trans*-**20c** were found to be the most active against *B. cereus* PCM 1948 (MIC = 0.625 mg/mL). *B. cereus* is a Gram-positive spore-forming bacterium commonly found in the environment and can contaminate food. *B. cereus* bacteria can multiply rapidly at room temperature and produce toxins that can cause food poisoning of the diarrheal and vomiting type. *B. cereus* is also associated with infections of the eye, respiratory tract, and wounds [26,27]. Alkene **24c** and isoxazolidines *cis*-**20a**/*trans*-**20a**, *cis*-**21c**/*trans*-**21c** and *cis*-**21d**/*trans*-**21d** showed noticeable activity against *E. coli* ATCC 25922 (MIC = 1.25 mg/mL). None of the synthesized compounds exhibited antifungal activity against tested strains (Table 3).

Since mutagenic compounds can be capable of inducing cancer [28], we decided to evaluate the mutagenic potential of the most active agent, i.e., the inseparable mixture of compounds *cis*-**20c/***trans*-**20c** (6:94) (Table 3) using the Ames mutagenicity assay (the bacterial reverse mutation test). The study was performed using a standard microplate Ames MPF^TM^ Penta I kit (Xenometrix, Allschwil, Switzerland), compliant with the OECD guideline 417 [29] and the International Organization for Standardization [30,31]. This test is a widely accepted short-term bacterial assay for the identification of substances that can produce genetic damage that leads to gene mutations. The bacterial strains used in this assay have various mutations that inactivate a gene involved in the synthesis of essential amino acids, either histidine (*Salmonella typhimurium*, TA98, TA100, TA1535 and TA1537 strains) or tryptophan (*Escherichia coli*, WP2uvrA[pKM101 strain), so they can only grow in the culture medium that is supplemented with that amino acid. The mutagenic potential of the sample was assessed after metabolic activation in the presence of Aroclor 1254-induced rat liver S9 (S9 Cofactor kit, Xenometrix, Allschwil, Switzerland). When the bacteria are exposed to a mutagen, mutations occur that may restore or reverse the ability of the bacteria to synthesize the amino acid and to continue growing once the limited amount of amino acid in the liquid medium is depleted. The following mutagenic compounds were used as positive controls: 2-aminoanthracene (for *S. typhimurium* TA98, TA100, TA1535 and TA1537) and 2-aminofluorene (for *E.coli* strain WP2 uvrA[pKM101]). As the negative control, 50% DMSO was used (solvent for the tested compound). Based on the obtained results, the tested agent, the inseparable mixture of compounds *cis*-**20c/***trans*-**20c** (6:94), should be considered as not mutagenic in the tested species of bacteria at the concentration up to 0.625 mg/mL.

## 3. Materials and Methods

### 3.1. General Information

^1^H, ^13^C, and ^31^P NMR spectra were taken in CDCl_3_ on the Bruker Avance III spectrometers (600 MHz) with TMS as internal standard at 600, 151, and 243 MHz, respectively. IR spectra were measured on an Infinity MI-60 FT-IR spectrometer. Melting points were determined on a Boetius apparatus and were uncorrected. Elemental analyses were performed by the Microanalytical Laboratory of this faculty on Perkin-Elmer PE 2400 CHNS analyzer. The following adsorbents were used: column chromatography, Merck silica gel 60 (70–230 mesh), analytical TLC, and Merck TLC plastic sheets silica gel 60 F_254_. *N*-methyl- and *N*-benzyl-*C*-(diethoxyphosphonyl)nitrones **14** and **15** were obtained according to procedures in the literature [19].

^1^H-, ^13^C- and ^31^P-NMR spectra of all new synthesized compounds are provided in Supplementary Materials.

### 3.2. General Procedure for Benzylation of 2H-Benzo[d][1,3]Oxazine-2,4-Diones ***25***

Sodium hydride (1.10 mmol) was added under argon atmosphere to a solution of 2*H-*benzo[*d*][1,3]oxazine-2,4-dione **25** (1.00 mmol) in anhydrous DMF (3 mL) and stirred at room temperature for 1 h. The respective benzyl bromide (1.10 mmol) was added and the reaction mixture was stirred for 18 h. The reaction mixture was poured onto ice water (20 mL). The suspension was filtered, washed with water (3 × 10 mL), and dried and crystallized from a chloroform-petroleum ether mixture to produce **26a–d**.

**1-Benzyl-2*H*-benzo[*d*][1,3]oxazine-2,4**(**1*H***)**-dione** (**26a**)**.** According to the general procedure from 2*H*-benzo[*d*][1,3]oxazine-2,4(1*H*)-dione **25** (1.00 g, 6.13 mmol), sodium hydride (0.162 g, 6.74 mmol), and benzyl bromide (0.838 mL, 6.74 mmol), 1-benzyl-2*H*-benzo[*d*][1,3]oxazine-2,4(1*H*)-dione **26a** (1.14 g, 73%) was obtained as an amorphous solid. M.p. 141–143 °C (lit. m.p. 140–142 °C) [20]. IR (KBr, cm^–1^) ν_max_: 3054, 2924, 1780, 1719, 1604, 1453, 1320, 1242, 1027, 758, 683. ^1^H NMR (600 MHz, CDCl_3_): δ = 8.19 (dd, *J* = 7.8 Hz, *J* = 1.4 Hz, 1H), 7.67–7.65 (m, 1H), 7.40–7.38 (m, 2H), 7.34–7.32 (m, 3H), 7.29 (t, *J* = 7.8 Hz, 1H), 7.15 (d, *J* = 8.5 Hz, 1H), 5.34 (s, 2H, C*H_2_*). ^13^C NMR (150 MHz, CDCl_3_): δ = 158.32 (C(O)), 148.48 (C(O)), 141.45, 137.17, 134.44, 130.87, 129.16, 128.15, 126.62, 124,15, 114.73, 111.90, 48.55. Anal. calcd. for C_15_H_11_NO_3_: C, 71.14; H, 4.38; N, 5.53. Found: C, 71.10; H, 4.16; N; 5.62.

**1-**(**2-Fluorobenzyl**)**-2*H*-benzo[*d*][1,3]oxazine-2,4**(**1*H***)**-dione** (**26b**)**.** According to the general procedure from 2*H*-benzo[*d*][1,3]oxazine-2,4(1*H*)-dione **25** (1.00 g, 6.13 mmol), sodium hydride (0.162 g, 6.74 mmol), and 2-fluorobenzyl bromide (0.838 mL, 6.74 mmol), 1-(2-fluorobenzyl)-2*H*-benzo[*d*][1,3]oxazine-2,4(1*H*)-dione **26b** (1.16 g, 70%) was obtained as an amorphous solid, m.p. 149–151.5 °C (lit. m.p. 151–154 °C) [20]. IR (KBr, cm^–1^) ν_max_: 3052, 2932, 1787, 1720, 1585, 1456, 1331, 1228, 1034, 788, 681. ^1^H NMR (600 MHz, CDCl_3_): δ = 8.20 (dd, *J* = 7.9 Hz, *J* = 1.5 Hz, 1H), 7.71–7.68 (m, 1H), 7.34–7.27 (m, 3H), 7.17–7.12 (m, 3H), 5.40 (s, 2H, C*H_2_*). ^13^C NMR (150 MHz, CDCl_3_): δ = 160.27 (d, ^1^*J*_(CF)_ = 246.4 Hz, C2’), 158.17 (C(O)), 148.53 (C(O)), 141.10, 137.35, 130.95, 129.99 (d, ^3^*J*_(CCCF)_ = 7.9 Hz, C6’), 128.43 (d, ^3^*J*_(CCCF)_ = 3.7 Hz, C4’), 124.92 (d, ^4^*J*_(CCCCF)_ = 3.9 Hz, C5’), 124.30, 121.57 (d, ^2^*J*_(CCF)_ = 13.4 Hz, C3’), 115.86, 115.79 (d, ^2^*J*_(CCF)_ = 21.2 Hz, C1’), 114.26 (d, ^5^*J*_(CNCCCF)_ = 2.2 Hz), 111.85, 42.11 (d, ^3^*J*_(CCCF)_ = 4.9 Hz). Anal. calcd. for C_15_H_10_FNO_3_: C, 66.42; H, 3.72; N, 5.16. Found: C, 66.71; H, 3.50; N, 5.29.

**1-**(**3-Fluorobenzyl**)**-2*H*-benzo[*d*][1,3]oxazine-2,4**(**1*H***)**-dione** (**26c**)**.** According to the general procedure from 2*H*-benzo[*d*][1,3]oxazine-2,4(1*H*)-dione **25** (1.00 g, 6.13 mmol), sodium hydride (0.162 g, 6.74 mmol), and 3-fluorobenzyl bromide (0.838 mL, 6.74 mmol), 1-(3-fluorobenzyl)-2*H*-benzo[*d*][1,3]oxazine-2,4(1*H*)-dione **26c** (1.18 g, 71%) was obtained as an amorphous solid. M.p. 131–133 °C (lit. m.p.133–136 °C) [20]. IR (KBr, cm^–1^) ν_max_: 3058, 2926, 1786, 1723, 1592, 1451, 1321, 1251, 1029, 791, 681. ^1^H NMR (600 MHz, CDCl_3_): δ = 8.22 (dd, *J* = 7.9 Hz, *J* = 1.4 Hz, 1H), 7.70–7.67 (m, 1H), 7.38–7.36 (m, 1H), 7.32 (t, *J* = 7.7 Hz, 1H), 7.12 (d, *J* = 7.9 Hz, 1H), 7.10 (d, *J* = 8.4 Hz, 1H), 7.05–7.02 (m, 2H), 5.33 (s, 2H, C*H_2_*). ^13^C NMR (150 MHz, CDCl_3_): δ = 163.24 (d, ^1^*J*_(CF)_ = 247.8 Hz, C3), 158.09 (C(O)), 148.40 (C(O)), 141.23, 137.00 (d, ^3^*J*_(CCCF)_ = 7.1 Hz, C5), 131.04, 130.87 (d, ^3^*J*_(CCCF)_ = 8.3 Hz, C1), 124.34, 122.19 (d, ^4^*J*_(CCCCF)_ = 2.8 Hz, C6), 115.23 (d, ^2^*J*_(CCF)_ = 21.2 Hz, C2), 114.46, 113.75 (d, ^2^*J*_(CCF)_ = 22.6 Hz, C4), 111.91, 48.06. Anal. calcd. for C_15_H_10_FNO_3_: C, 66.42; H, 3.72; N, 5.16. Found: C, 66.72; H, 3.56; N, 5.27.

**1-**(**4-Fluorobenzyl**)**-2*H*-benzo[*d*][1,3]oxazine-2,4**(**1*H***)**-dione** (**26d**)**.** According to the general procedure from 2*H*-benzo[*d*][1,3]oxazine-2,4(1*H*)-dione **25** (1.00 g, 6.13 mmol), sodium hydride (0.162 g, 6.74 mmol), and 4-fluorobenzyl bromide (0.838 mL, 6.74 mmol), 1-(4-fluorobenzyl)-2*H*-benzo[*d*][1,3]oxazine-2,4(1*H*)-dione **26d** (1.29 g, 78%) was obtained as an amorphous solid. M.p. 141–143 °C (lit. m.p. 143–145 °C) [20]. IR (KBr, cm^–1^) ν_max_: 3022, 2924, 1702, 1680, 1607, 1485, 1324, 1221, 1018, 759, 686. ^1^H NMR (600 MHz, CDCl_3_): δ = 8.22 (dd, *J* = 7.9 Hz, *J* = 1.4 Hz, 1H), 7.70–7.67 (m, 1H), 7.38–7.36 (m, 1H), 7.32 (t, *J* = 7.7 Hz, 1H), 7.12 (d, *J* = 7.9 Hz, 1H), 7.10 (d, *J* = 8.4 Hz, 1H), 7.05–7.02 (m, 2H), 5.33 (s, 2H, C*H_2_*). ^13^C NMR (150 MHz, CDCl_3_): δ = 162.46 (d, ^1^*J*_(CF)_ = 247.4 Hz, C4), 158.21 (C(O)), 148.46 (C(O)), 141.24, 137.27, 131.10, 130.21 (d, ^4^*J*_(CCCCF)_ = 3.2 Hz, C1), 128.56 (d, ^3^*J*_(CCCF)_ = 8.5 Hz, C2, C6), 121.31, 116.18 (d, ^2^*J*_(CCF)_ = 21.9 Hz, C3, C5), 114.52, 46.57. Anal. calcd. for C_15_H_10_FNO_3_: C, 66.42; H, 3.72; N, 5.16. Found: C, 66.61; H, 3.51; N, 5.27.

### 3.3. General Procedure for the Synthesis of 1-Benzylquinazoline-2,4-Diones ***26a–d***

Urea (1.50 mmol) was added to a solution of the respective 1-benzylbenzo[*d*][1,3]oxazine-2,4-dione **26a–d** (1.00 mmol) in anhydrous DMF (10 mL) and the mixture was heated under reflux for 5 h. The solvent was removed in vacuo and the residue was crystallized from ethanol to produce the corresponding **27a–d**.

**1-Benzylquinazoline-2,4-dione** (**27a**)**.** According to the general procedure from 1-benzylbenzo[*d*][1,3]oxazine-2,4-dione **26a** (0.500 g, 1.97 mmol) and urea (0.178 g, 2.96 mmol), 1-benzylquinazoline-2,4-dione **27a** (0.238 g, 48%) was obtained as a white amorphous solid, m.p. 218–220 °C. IR (KBr, cm^–1^) ν_max_: 3170, 2923, 1704, 1605, 1480, 1311, 1151, 1020, 856, 722. ^1^H NMR (600 MHz, CDCl_3_): δ = 8.70 (s, 1H, N*H*), 8.25 (dd, *J* = 7.9 Hz, *J* = 1.5 Hz, 1H), 7.60–7.58 (m, 1H), 7.38–7.36 (m, 2H), 7.32–7.29 (m, 3H), 7.26 (t, *J* = 7.9 Hz, 1H), 7.16 (t, *J* = 8.5 Hz, 1H), 5.39 (s, 2H, C*H_2_*). ^13^C NMR (150 MHz, CDCl_3_): δ = 161.84 (C(O)), 150.72 (C(O)), 141.11, 135.56, 135.39, 129.04, 128.81, 127.78, 126.50, 123.32, 116.13, 114.95, 46.58. Anal. calcd. for C_15_H_11_FN_2_O_2_: C, 71.42; H, 4.79; N, 11.10. Found: C, 71.12; H, 4.54; N, 10.87.

**1-**(**2-Fluorobenzyl**)**quinazoline-2,4-dione** (**27b**)**.** According to the general procedure from 1-(2-fluorobenzyl)benzo[*d*][1,3]oxazine-2,4-dione **26b** (0.500 g, 1.84 mmol) and urea (0.166 g, 2.76 mmol), 1-(2-fluorobenzyl)quinazoline-2,4-dione **27b** (0.253 g, 49%) was obtained as a white amorphous solid, m.p. = 228–230 °C. IR (KBr, cm^–1^) ν_max_: 3177, 2922, 1701, 1606, 1457, 1371, 1230, 1020, 852, 749. ^1^H NMR (600 MHz, CDCl_3_): δ = 8.42 (s, 1H, N*H*), 8.25 (dd, *J* = 7.9 Hz, *J* = 1.5 Hz, 1H), 7.64–7.61 (m, 1H), 7.32–7.27 (m, 2H), 7.19–7.13 (m, 3H), 7.11–7.09 (m, 1H), 5.44 (s, 2H, C*H_2_*). ^13^C NMR (150 MHz, CDCl_3_): δ = 161.49 (C(O)), 160.31 (d, ^1^*J*_(CF)_ = 245.7 Hz, C2’), 150.58 (C(O)), 140.79, 135.72, 129.53 (d, ^3^*J*_(CCCF)_ = 8.0 Hz, C6’), 128.87, 128.18 (d, ^3^*J*_(CCCF)_ = 3.9 Hz, C4’), 124.76 (d, ^4^*J*_(CCCCF)_ = 3.9 Hz, C5’), 123.47, 122.46 (d, ^2^*J*_(CCF)_ = 14.2 Hz, C3’), 116.11, 115.65 (d, ^2^*J*_(CCF)_ = 21.7 Hz, C1’), 114.47 (d, ^5^*J*_(CNCCCF)_ =1.7 Hz), 40.18 (d, ^3^*J*_(CCCF)_ = 5.4 Hz). Anal. calcd. for C_15_H_11_FN_2_O_2_: C, 66.66; H, 4.10; N, 10.37. Found: C, 66.55; H, 3.90; N, 10.60.

**1-**(**3-Fluorobenzyl**)**quinazoline-2,4-dione** (**27c**)**.** According to the general procedure from 1-(3-fluorobenzyl)benzo[*d*][1,3]oxazine-2,4-dione **26c** (0.500 g, 1.84 mmol) and urea (0.166 g, 2.76 mmol), 1-(3-fluorobenzyl)quinazoline-2,4-dione **27c** (0.225 g, 45%) was obtained as a white amorphous solid, m.p. = 227–229 °C. IR (KBr, cm^–1^) ν_max_: 3176, 2964, 1691, 1607, 1439, 1317, 1244, 1020, 939, 738. ^1^H NMR (600 MHz, CDCl_3_): δ = 8.65 (s, 1H, N*H*), 8.25 (dd, *J* = 7.9Hz, *J* = 1.5 Hz, 1H), 7.63–7.60 (m, 1H), 7.36–7.34 (m, 1H), 7.33–7.27 (m, 1H), 7.12 (d, *J* = 8.5 Hz, 1H), 7.09 (d, *J* = 7.9 Hz, 1H), 7.02–6.99 (m, 2H), 5.37 (s, 2H, C*H_2_*). ^13^C NMR (150 MHz, CDCl_3_): δ = 163.25 (d, ^1^*J*_(CF)_ = 247.7 Hz, C3), 161.46 (C(O)), 150.47 (C(O)), 140.91, 138.03 (d, ^3^*J*_(CCCF)_ = 6.8 Hz, C5), 135.61, 130.68 (d, ^3^*J*_(CCCF)_ = 7.9 Hz, C1), 128.96, 123.50, 122.07 (d, ^4^*J*_(CCCCF)_ = 2.6 Hz, C6), 116.17, 114.85 (d, ^2^*J*_(CCF)_ = 21.0 Hz, C2), 114.66, 113.61 (d, ^2^*J*_(CCF)_ = 22.1 Hz, C4), 45.05 (d, ^4^*J*_(CCCCF)_ = 1.6 Hz). Anal. calcd. for C_15_H_11_FN_2_O_2_ × 0.5 H_2_O: C, 64.51; H, 4.33; N, 10.03. Found: C, 64.41; H, 4.03; N, 10.31.

**1-**(**4-Fluorobenzyl**)**quinazoline-2,4-dione** (**27d**)**.** According to the general procedure from 1-(4-fluorobenzyl)benzo[*d*][1,3]oxazine-2,4-dione **26d** (0.500 g, 1.84 mmol) and urea (0.166 g, 2.76 mmol), 1-(4-fluorobenzyl)quinazoline-2,4-dione **27d** (0.250 g, 50%) was obtained as a white amorphous solid, m.p. = 214–215 °C. IR (KBr, cm^–1^) ν_max_: 3153, 3020, 2922, 1679, 1606, 1437, 1321, 1224, 1018, 920, 759. ^1^H NMR (600 MHz, CDCl_3_): δ = 9.08 (s, 1H, N*H*), 8.26 (dd, *J* = 7.9Hz, *J* = 1.5 Hz, 1H), 7.62–7.60 (m, 1H), 7.31–7.26 (m, 3H), 7.15 (d, *J* = 8.5 Hz, 1H), 7.07–7.04 (m, 2H), 5.35 (s, 2H, C*H_2_*). ^13^C NMR (150 MHz, CDCl_3_): δ = 162.29 (d, ^1^*J*_(CF)_ = 246.8 Hz, C4), 161.71 (C(O)), 150.68 (C(O)), 140.96, 135.53, 131.18 (d, ^4^*J*_(CCCCF)_ = 3.2 Hz, C1), 128.94, 128.36 (d, ^3^*J*_(CCCF)_ = 7.9 Hz, C2, C6), 123.41, 116.21, 115.98 (d, ^2^*J*_(CCF)_ = 21.9 Hz, C3, C5), 114.68, 45.93. Anal. calcd. for C_15_H_11_FN_2_O_2_: C, 66.66; H, 4.10; N, 10.37. Found: C, 66.83; H, 4.34; N, 10.57.

### 3.4. General Procedure for Allylation of 1-Benzylquinazolin-2,4-Dione ***27a–d***

Allyl bromide (2.20 mmol) was added to a suspension of the respective 1-benzylquinazoline-2,4-dione **27a–d** (1.00 mmol) and potassium hydroxide (3.00 mmol) in anhydrous acetonitrile (15 mL). The reaction mixture was stirred at 105 °C for 4 h. The solvent was removed in vacuo and the residue was dissolved in methylene chloride (10 mL) and extracted with water (3 × 10 mL). The organic layer was dried (MgSO_4_), concentrated, and purified by column chromatography with a chloroform–hexane mixture (7:3, *v/v*), then crystallized from a chloroform–petroleum ether mixture to produce compounds **24a–d**.

**3-Allyl-1-benzylquinazoline-2,4-dione** (**24a**)**.** According to the general procedure from 1-benzylquinazoline-2,4-dione **27a** (0.150 g, 0.594 mmol), potassium hydroxide (0.100 g, 1.78 mmol), and allyl bromide (0.113 mL, 1.31 mmol), 3-allyl-1-benzylquinazoline-2,4-dione **24a** (0.174g, 89%) was obtained as a white amorphous solid, m.p. = 101–103 °C. IR (KBr, cm^–1^) ν_max_: 3063, 2963, 1701, 1606, 1454, 1341, 1272, 1025, 941, 762. ^1^H NMR (600 MHz, CDCl_3_): δ = 8.27 (dd, *J* = 7.9 Hz, *J* = 1.4 Hz, 1H), 7.58–7.55 (m, 1H), 7.37–7.35 (m, 2H), 7.31–7.28 (m, 3H), 7.25–7.23 (m, 1H), 7.18 (d, *J* = 8.4 Hz, 1H), 6.03 (ddt, ^3^*J* = 17.1 Hz, ^3^*J* = 10.2 Hz, ^3^*J* = 5.8 Hz, 1H, CH_2_–C*H*=CH_2_), 5.42 (s, 2H, C*H_2_*Ph), 5.37 (dd, ^3^*J* = 17.1 Hz, ^2^*J* = 1.3 Hz, 1H, CH_2_–CH=C*H*H), 5.26 (dd, ^3^*J* = 10.2 Hz, ^2^*J* = 1.3 Hz, 1H, CH_2_–CH=CH*H*), 4.80 (d, ^3^*J* = 5.8 Hz, 2H, C*H_2_*–CH=CH_2_). ^13^C NMR (150 MHz, CDCl_3_): δ = 161.46 (C(O)), 151.17 (C(O)), 140.02, 135.74, 135.04, 131.93, 129.10, 128.98, 127.62, 126,48, 123.07, 117.64, 115.93, 115.80, 47.34, 44.01. Anal. calcd. for C_18_H_16_N_2_O_2_: C, 73.95; H, 5.52; N, 9.58. Found: C, 73.87; H, 5.23; N; 9.77.

**3-Allyl-1-**(**2-fluorobenzyl**)**quinazoline-2,4-dione** (**24b**)**.** According to the general procedure from 1-(2-fluorobenzyl)quinazoline-2,4-dione **27b** (0.250 g, 0.925 mmol), potassium hydroxide (0.156 g, 2.78 mmol), and allyl bromide (0.176 mL, 2.04 mmol), 3-allyl-1-(2-fluorobenzyl)quinazoline-2,4-dione **24b** (0.269 g, 94%) was obtained as a white amorphous solid, m.p. = 93–94 °C. IR (KBr, cm^–1^) ν_max_: 3086, 2988, 1703, 1660, 1606, 1454, 1339, 1225, 1025, 935, 756. ^1^H NMR (600 MHz, CDCl_3_): δ = 8.28 (dd, *J* = 7.9 Hz, *J* = 1.4 Hz, 1H), 7.61–7.58 (m, 1H), 7.31–7.25 (m, 2H), 7.15–7.11 (m, 3H), 7.10–7.07 (m, 1H), 5.95 (ddt, ^3^*J* = 17.1 Hz, ^3^*J* = 10.2 Hz, ^3^*J* = 5.8 Hz, 1H, CH_2_–C*H*=CH_2_), 5.47 (s, 2H, C*H_2_*Ph), 5.36 (dd, ^3^*J* = 17.1 Hz, ^2^*J* = 1.3 Hz 1H, CH_2_–CH=C*H*H), 5.27 (d, ^3^*J* = 10.2 Hz, ^2^*J* = 1.3 Hz, 1H, CH_2_–CH=CH*H*), 4.80 (d, ^3^*J* = 5.8 Hz, 2H, C*H_2_*–CH=CH_2_). ^13^C NMR (150 MHz, CDCl_3_): δ = 161.39 (C(O)), 160.33 (d, ^1^*J*_(CF)_ = 246.2 Hz, C2), 151.23 (C(O)), 139.69, 135.22, 131.85, 129.39 (d, ^3^*J*_(CCCF)_ = 8.0 Hz, C6), 129.16, 128.10 (d, ^3^*J*_(CCCF)_ = 4.0 Hz, C4), 124.70 (d, ^4^*J*_(CCCCF)_ = 3.6 Hz, C5), 123.23, 122.78 (d, ^2^*J*_(CCF)_ = 13.8 Hz, C3), 117.96, 115.78, 115.61 (d, ^2^*J*_(CCF)_ = 21.7 Hz, C1), 113.97 (d, ^5^*J* = 1.7 Hz), 44.03, 40.97 (d, ^3^*J*_(CCCF)_ = 5.4 Hz). Anal. calcd. for C_18_H_15_FN_2_O_2_: C, 69.67; H, 4.87; N, 9.03. Found: C, 69.77; H, 4.60; N, 9.26.

**3-Allyl-1-**(**3-fluorobenzyl**)**quinazoline-2,4-dione** (**24c**)**.** According to the general procedure from 1-(3-fluorobenzyl)quinazoline-2,4-dione **27c** (0.260 g, 0.962 mmol), potassium hydroxide (0.162 g, 2.89 mmol), and allyl bromide (0.183 mL, 2.12 mmol), 3-allyl-1-(3-fluorobenzyl)quinazoline-2,4-dione **24c** (0.249 g, 83%) was obtained as a white amorphous solid, m.p. = 115–116 °C. IR (KBr, cm^–1^) ν_max_: 3091, 2963, 1700, 1655, 1604, 1485, 1342, 1252, 1001, 940, 929, 765. ^1^H NMR (600 MHz, CDCl_3_): δ = 8.28 (dd, *J* = 7.9 Hz, *J* = 1.4 Hz 1H), 7.60–7.57 (m, 1H), 7.35–7.32 (m, 1H), 7.29–7.26 (m, 1H), 7.10 (d, *J* = 8.5 Hz, 1H), 7.07 (d, *J* = 7.8 Hz, 1H), 7.01–6.97 (m, 2H), 5.95 (ddt, ^3^*J* = 17.1 Hz, ^3^*J* = 10.2 Hz, ^3^*J* = 5.8 Hz, 1H, CH_2_–C*H*=CH_2_), 5.40 (s, 2H, C*H_2_*Ph), 5.36 (dd, ^3^*J* = 17.1 Hz, ^2^*J* = 1.3 Hz, 1H, CH_2_–CH=C*H*H), 5.24 (d, ^3^*J* = 10.2 Hz, ^2^*J* = 1.3 Hz, 1H, CH_2_–CH=CH*H*), 4.80 (d, ^3^*J* = 5.8 Hz, 2H, C*H_2_*–CH=CH_2_). ^13^C NMR (150 MHz, CDCl_3_): δ = 163.23 (d, ^1^*J*_(CF)_ = 247.6 Hz, C3), 161.33 (C(O)), 151.11 (C(O)), 139.81, 138.38 (d, ^3^*J*_(CCCF)_ = 7.0 Hz, C5), 135.12, 131.83, 130.62 (d, ^3^*J*_(CCCF)_ = 8.5 Hz, C1), 129.25, 123.26, 122.05 (d, ^4^*J*_(CCCCF)_ = 3.1 Hz, C6), 118.05, 115.83, 114.72 (d, ^2^*J*_(CCF)_ = 21.3 Hz, C2), 114.14, 113.57 (d, ^2^*J*_(CCF)_ = 22.1 Hz, C4), 46.91, 44.05. Anal. calcd. for C_18_H_15_FN_2_O_2_: C, 69.67; H, 4.87; N, 9.03. Found: C, 69.59; H, 4.58; N, 9.31.

**3-Allyl-1-**(**4-fluorobenzyl**)**quinazoline-2,4-dione** (**24d**)**.** According to the general procedure from 1-(4-fluorobenzyl)quinazoline-2,4-dione **27d** (0.215 g, 0.796 mmol), potassium hydroxide (0.134 g, 2.39 mmol), and allyl bromide (0.150 mL, 1.75 mmol), 3-allyl-1-(4-fluorobenzyl)quinazoline-2,4-dione **24d** (0.159 g, 64%) was obtained as a white amorphous solid, m.p. = 120–122 °C. IR (KBr, cm^–1^) ν_max_: 3155, 2924, 1702, 1660, 1607, 1485, 1324, 1221, 1051, 967, 743. ^1^H NMR (600 MHz, CDCl_3_): δ = 8.28 (dd, *J* = 7.9 Hz, *J* = 1.5 Hz, 1H), 7.60–7.57 (m, 1H), 7.29–7.25 (m, 3H), 7.13 (d, *J* = 8.5 Hz, 1H), 7.07–7.04 (m, 2H), 6.02 (ddt, ^3^*J* = 16.1 Hz, ^3^*J* = 10.2 Hz, ^3^*J* = 5.8 Hz, 1H, CH_2_–C*H*=CH_2_), 5.37 (s, 2H, C*H_2_*Ph), 5.36 (dd, ^3^*J* = 16.1 Hz, ^2^*J* = 1.4 Hz, 1H, CH_2_–CH=C*H*H), 5.26 (d, ^3^*J* = 10.2 Hz, ^2^*J* = 1.4 Hz, 1H, CH_2_–CH=CH*H*), 4.79 (d, ^3^*J* = 5.8 Hz, 2H, C*H_2_*–CH=CH_2_). ^13^C NMR (150 MHz, CDCl_3_): δ = 162.24 (d, ^1^*J*_(CF)_ = 246.3 Hz, C4), 161.35 (C(O)), 151.13 (C(O)), 139.85, 135.07, 131.87, 131.49 (d, ^4^*J*_(CCCCF)_ = 3.1 Hz, C1), 129.21, 128.31 (d, ^3^*J*_(CCCF)_ = 7.86 Hz, C2, C6), 123.18, 117.99, 115.93 (d, ^2^*J*_(CCF)_ = 21.8 Hz, C3, C5), 115.83, 114.17, 46.69, 44.01. Anal. calcd. for C_18_H_15_FN_2_O_2_: C, 69.67; H, 4.87; N, 9.03. Found: C, 69.76; H, 4.60; N, 9.27.

### 3.5. General Procedure for the Synthesis of Isoxazolidines cis-***20*** and trans-***20*** As Well As cis-***21*** and trans-***21***

Solutions of nitrones **14** or **15** (1.00 mmol) and the respective N3-allylated quinazoline-2,4-dione **24a–d** (1.00 mmol) in toluene were stirred at 60 °C until the starting nitrone disappeared. Solvents were evaporated in vacuo and the crude products were obtained as the mixtures of the respective diastereoisomeric isoxazolidines *cis*-**20**/*trans*-**20** or *cis*-**21**/*trans*-**21,** which were then purified on a silica gel purified on silica gel column chromatography with chloroform–hexane mixtures as eluents.

**Diethyl *trans-*5-**((**1-benzyl-2,4-dioxo-1,4-dihydroquinazolin-3**(**2*H***)**-yl**)**methyl**)**-2-methylisoxazolidin-3-yl**)**phosphonate** (***trans*-20a**)**.** Colorless oil. IR (film, cm^–1^) ν_max_: 2981, 1706, 1660, 1607, 1484, 1350, 1238, 1052, 1025, 968, 759. NMR signals of *trans*-**20a** were extracted from the spectra of 10:90 mixtures of *cis*-**20a** and *trans*-**20a**, ^1^H NMR (600 MHz, CDCl_3_): δ = 8.24 (dd, *J* = 7.9 Hz, *J* = 1.5 Hz, 1H), 7.56–7.54 (m, 1H), 7.35–7.32 (m, 2H), 7.29–7.26 (m, 3H), 7.23–7.21 (m, 1H), 7.13 (d, *J* = 8.5 Hz, 1H), 5.41 (AB, *J*_AB_ = 16.4 Hz, 1H, *H*CHN), 5.36 (AB, *J*_AB_ = 16.4 Hz, 1H, HC*H*N), 4.53–4.48 (m, 2H, *H*C5, HC*H*N), 4.26 (dd, ^2^*J* = 16.7 Hz, ^3^*J*_(HC–H5)_ = 8.4 Hz, 1H, *H*CHN), 4.23–4.16 (m, 4H, 2 × C*H_2_*OP), 3.10 (ddd, ^3^*J*_(H3–H4β)_ = 9.7 Hz, ^3^*J*_(H3–H4α)_ = 7.0 Hz, ^2^*J*_(H3–P)_ = 2.3 Hz, 1H, *H*C3), 2.91 (s, 3H, C*H*_3_N), 2.67 (dddd, ^3^*J*_(H4α–P)_ = 19.4 Hz, ^2^*J*_(H4α–H4β)_ = 12.4 Hz, ^3^*J*_(H4α–H3)_ = 7.0 Hz, ^3^*J*_(H4β–H5)_ = 7.0 Hz, 1H, *H*αC4), 2.42 (dddd, ^2^*J*_(H4β–H4α)_ = 12.4 Hz, ^3^*J*_(H4β–P)_ = 12.4 Hz, ^3^*J*_(H4β–H3)_ = 9.7 Hz, ^3^*J*_(H4β–H5)_ = 6.4 Hz, 1H, *H*βC4), 1.35 (t, ^3^*J* = 7.0 Hz, 3H, C*H_3_*CH_2_OP), 1.34 (t, ^3^*J* = 7.1 Hz, 3H, C*H_3_*CH_2_OP). ^13^C NMR (150 MHz, CDCl_3_): δ = 161.683 (C(O)), 151.38 (C(O)), 139.97, 135.66, 135.12, 129.15, 128.95, 127.66, 126.49, 123.09, 115.68, 114.38, 74.49 (d, ^3^*J*_(CCCP)_ = 7.7 Hz, C5), 63.89 (d, ^1^*J*_(CP)_ = 168.7 Hz, C3), 63.11 (d, ^2^*J*_(COP)_ = 6.4 Hz, CH*_2_*OP), 62.36 (d, ^2^*J*_(COP)_ = 6.3 Hz, CH*_2_*OP), 47.41 (CH_2_N), 46.51 (d, ^3^*J*_(CNCP)_ = 4.3 Hz, CH_3_N), 44.32 (*C*H_2_Ph), 36.27 (d, ^2^*J*_(CCP)_ = 1.7 Hz, C4), 16.50 (d, ^3^*J*_(CCOP)_ = 6.2 Hz, *C*H_3_CH_2_OP), 16.46 (d, ^3^*J*_(CCOP)_ = 6.3 Hz, *C*H_3_CH_2_OP). ^31^P NMR (243 MHz, CDCl_3_): δ = 22.14. Anal.calcd. for. C_24_H_30_N_3_O_6_P: C, 59.13; H, 6.20; N, 8.62. Found: C, 59.38; H, 5.97; N, 8.91 (obtained on 10:90 mixtures of *cis*-**20a** and *trans*-**20a**).

**Diethyl *trans*-**(**5-**((**1-**(**2-fluorobenzyl**)**-2,4-dioxo-1,4-dihydroquinazolin-3**(**2*H***)**-yl**)**methyl**)**-2-methylisoxazolidin-3-yl**)**phosphonate** (***trans*-20b**)**.** Colorless oil. IR (film, cm^–1^) ν_max_: 2981, 1707, 1661, 1608, 1483, 1351, 1231, 1052, 1023, 967, 756. NMR signals of *trans*-**20b** were extracted from the spectra of 8:92 mixtures of *cis*-**20b** and *trans*-**20b**, ^1^H NMR (600 MHz, CDCl_3_): δ = 8.24 (dd, *J* = 7.9 Hz, *J* = 1.5 Hz, 1H), 7.60–7.57 (m, 1H), 7.28–7.23 (m, 2H), 7.14–7.10 (m, 3H), 7.07–7.05 (m, 1H), 5.46 (AB, *J*_AB_ = 16.8 Hz, 1H, *H*CHN), 5.42 (AB, *J*_AB_ = 16.8 Hz, 1H, HC*H*N), 4.54–4.48 (m, 2H, *H*C5, *H*CHN), 4.29–4.25 (m, 1H, HC*H*N), 4.24–4.1 (m, 4H, 2 × C*H_2_*OP), 3.12–3.09 (m, 1H, *H*C3), 2.91 (s, 3H, C*H*_3_N), 2.68 (dddd, ^3^*J*_(H4α–P)_ = 19.8 Hz, ^2^*J*_(H4α–H4β)_ = 12.7 Hz, ^3^*J*_(H4α–H3)_ = 7.3 Hz, ^3^*J*_(H4β–H5)_ = 7.3 Hz, 1H, *H*αC4), 2.43 (dddd, ^2^*J*_(H4β–H4α)_ = 12.7 Hz, ^3^*J*_(H4β–P)_ = 12.7 Hz, ^3^*J*_(H4β–H3)_ = 11.2 Hz, ^3^*J*_(H4β–H5)_ = 6.7 Hz, 1H, *H*βC4), 1.35 (t, ^3^*J* = 7.1 Hz, 3H, C*H_3_*CH_2_OP), 1.34 (t, ^3^*J* = 7.1 Hz, 3H, C*H_3_*CH_2_OP). ^13^C NMR (150 MHz, CDCl_3_): δ = 161.62 (C(O)), 160.30 (d, ^1^*J*_(CF)_ = 245.6 Hz, C2’), 151.44 (C(O)), 139.65, 135.23, 129.40 (d, ^3^*J*_(CCCF)_ = 8.0 Hz, C6’), 129.21, 128.13 (d, ^3^*J*_(CCCF)_ = 3.7 Hz, C4’), 124.69 (d, ^4^*J*_(CCCCF)_ = 3.3 Hz, C5’), 123.26, 122.69 (d, ^2^*J*_(CCF)_ = 14.0 Hz, C3’), 115.66, 115.57 (d, ^2^*J*_(CCF)_ = 21.9 Hz, C1’), 113.97 (d, ^5^*J* = 1.4 Hz), 74.46 (d, ^3^*J*_(CCCP)_ = 7.7 Hz, C5), 63.86 (d, ^1^*J*_(CP)_ = 168.6 Hz, C3), 63.15 (d, ^2^*J*_(COP)_ = 6.5 Hz, CH*_2_*OP), 62.38 (d, ^2^*J*_(COP)_ = 7.1 Hz, CH*_2_*OP), 46.44 (d, ^3^*J*_(CNCP)_ = 3.6 Hz, CH_3_N), 44.33 (CH_2_N), 41.01 (d, ^3^*J*_(CCCF)_ = 5.2 Hz, *C*H_2_Ph), 35.22 (d, ^2^*J*_(CCP)_ = 1.6 Hz, C4), 16.50 (d, ^3^*J*_(CCOP)_ = 6.3 Hz, *C*H_3_CH_2_OP), 16.46 (d, ^3^*J*_(CCOP)_ = 5.9 Hz, *C*H_3_CH_2_OP). ^31^P NMR (243 MHz, CDCl_3_): δ = 21.95. Anal.calcd. for C_24_H_29_FN_3_O_6_P × 1.5 H_2_O: C, 54.13; H, 6.06; N, 7.89. Found: C, 54.16; H, 5.77; N, 7.61 (obtained on 8:92 mixtures of *cis*-**20b** and *trans*-**20b**).

**Diethyl *trans*-**(**5-**((**1-**(**3-fluorobenzyl**)**-2,4-dioxo-1,4-dihydroquinazolin-3**(**2*H***)**-yl**)**methyl**)**-2-methylisoxazolidin-3-yl**)**phosphonate** (***trans*-20c**)**.** Colorless oil. IR (film, cm^–1^) ν_max_: 2983, 1706, 1653, 1609, 1484, 1401, 1346, 1251, 1097, 1024, 967, 762. NMR signals of *trans*-**20c** were extracted from the spectra of 8:92 mixtures of *cis*-**20c** and *trans*-**20c**, ^1^H NMR (600 MHz, CDCl_3_): δ = 8.23 (dd, *J* = 7.9 Hz, *J* = 1.2 Hz, 1H), 7.57–7.55 (m, 1H), 7.32–7.28 (m, 1H), 7.24–7.22 (m, 1H), 7.07 (d, *J* = 8.5 Hz, 1H), 7.05 (d, *J* = 7.8 Hz, 1H), 6.97–6.94 (m, 2H), 5.40 (AB, *J*_AB_ = 16.6 Hz, 1H, *H*CHN), 5.31 (AB, *J*_AB_ = 16.6 Hz, 1H, HC*H*N), 4.51–4.46 (m, 2H, *H*C5, *H*CHN), 4.25 (dd, ^2^*J* = 16.4 Hz, ^3^*J*_(HC–H5)_ = 8.1 Hz, 1H, *H*CHN), 4.21–4.14 (m, 4H, 2 × C*H_2_*OP), 3.10–3.07 (m, 1H, *H*C3), 2.89 (s, 3H, C*H*_3_N), 2.66 (dddd, ^3^*J*_(H4α–P)_ = 19.3 Hz, ^2^*J*_(H4α–H4β)_ = 12.8 Hz, ^3^*J*_(H4α–H3)_ = 7.0 Hz, ^3^*J*_(H4β–H5)_ = 7.0 Hz, 1H, *H*αC4), 2.40 (dddd, ^2^*J*_(H4β–H4α)_ = 12.8 Hz, ^3^*J*_(H4β–P)_ = 12.8 Hz, ^3^*J*_(H4β–H3)_ = 10.2 Hz, ^3^*J*_(H4β–H5)_ = 6.8 Hz, 1H, *H*βC4), 1.34 (t, ^3^*J* = 7.0 Hz, 3H, C*H_3_*CH_2_OP), 1.34 (t, ^3^*J* = 7.1 Hz, 3H, C*H_3_*CH_2_OP). ^13^C NMR (150 MHz, CDCl_3_): δ = 163.19 (d, ^1^*J*_(CF)_ = 246.9 Hz, C3’), 161.55 (C(O)), 151.32 (C(O)), 139.75, 138.32 (d, ^3^*J*_(CCCF)_ = 7.2 Hz, C5’), 135.22, 130.58 (d, ^3^*J*_(CCCF)_ = 8.0 Hz, C1’), 129.26, 123.27, 122.10 (d, ^4^*J*_(CCCCF)_ = 2.4 Hz, C6’), 115.69, 114.71 (d, ^2^*J*_(CCF)_ = 21.1 Hz, C2’), 114.11, 113.60 (d, ^2^*J*_(CCF)_ = 22.1 Hz, C4’), 75.43 (d, ^3^*J*_(CCCP)_ = 7.7 Hz, C5), 63.88 (d, ^1^*J*_(CP)_ = 168.8 Hz, C3), 63.11 (d, ^2^*J*_(COP)_ = 6.3 Hz, CH*_2_*OP), 62.36 (d, ^2^*J*_(COP)_ = 7.2 Hz, CH*_2_*OP), 46.95 (CH_2_N), 46.46 (d, ^3^*J*_(CNCP)_ = 4.1 Hz, CH_3_N), 44.32 (*C*H_2_Ph), 35.06 (d, ^2^*J*_(CCP)_ = 1.4 Hz, C4), 16.49 (d, ^3^*J*_(CCOP)_ = 6.3 Hz, *C*H_3_CH_2_OP), 16.44 (d, ^3^*J*_(CCOP)_ = 6.6 Hz, *C*H_3_CH_2_OP). ^31^P NMR (243 MHz, CDCl_3_): δ = 22.09. Anal.calcd. for C_24_H_29_FN_3_O_6_P × 2.5 H_2_O: C, 52.36; H, 6.23; N, 7.63. Found: C, 52.35; H, 5.95; N, 7.42 (obtained on 8:92 mixtures of *cis*-**20c** and *trans*-**20c**).

**Diethyl *trans*-**(**5-**((**1-**(**4-fluorobenzyl**)**-2,4-dioxo-1,4-dihydroquinazolin-3**(**2*H***)**-yl**)**methyl**)**-2-methylisoxazolidin-3-yl**)**phosphonate** (***trans*-20d**)**.** Colorless oil. IR (film, cm^–1^) ν_max_: 2983, 1706, 1661, 1608, 1483, 1329, 1232, 1052, 967, 1024, 756. NMR signals of *trans*-**20d** were extracted from the spectra of 8:92 mixtures of *cis*-**20d** and *trans*-**20d**, ^1^H NMR (600 MHz, CDCl_3_): δ = 8.13 (dd, *J* = 7.9 Hz, *J* = 1.4 Hz, 1H), 7.60–7.57 (m, 1H), 7.28–7.24 (m, 3H), 7.12–7.28 (d, *J* = 8.4 Hz, 1H), 7.06–7.03 (m, 2H), 5.31 (AB, *J*_AB_ = 16.3 Hz, 1H, *H*CHN), 5.32 (AB, *J*_AB_ = 16.3 Hz, 1H, HC*H*N), 4.61–4.52 (m, 1H, *H*C5), 4.50 (dd, ^2^*J* = 12.4 Hz, ^3^*J*_(HC–H5)_ = 7.6 Hz, 1H, *H*CHN), 4.26 (dd, ^2^*J* = 12.4 Hz, ^3^*J*_(HC–H5)_ = 3.8 Hz, 1H, HC*H*N), 4.24–4.17 (m, 4H, 2 × C*H_2_*OP), 3.14–3.12 (m, 1H, *H*C3), 2.70 (dddd, ^3^*J*_(H4α–P)_ = 19.8 Hz, ^2^*J*_(H4α–H4β)_ = 12.6 Hz, ^3^*J*_(H4α–H3)_ = 7.3 Hz, ^3^*J*_(H4β–H5)_ = 7.3 Hz, 1H, *H*αC4), 2.43 (dddd, ^2^*J*_(H4β–H4α)_ = 12.6 Hz, ^3^*J*_(H4β–P)_ = 12.6 Hz, ^3^*J*_(H4β–H3)_ = 9.9 Hz, ^3^*J*_(H4β–H5)_ = 6.7 Hz, 1H, *H*βC4), 1.37 (t, ^3^*J* = 7.1 Hz, 3H, C*H_3_*CH_2_OP), 1.36 (t, ^3^*J* = 7.1 Hz, 3H, C*H_3_*CH_2_OP). ^13^C NMR (150 MHz, CDCl_3_): δ = 162.25 (d, ^1^*J*_(CF)_ = 246.3 Hz, C4’), 161.44 (C(O)), 151.35 (C(O)), 139.82, 135.19, 130.89 (d, ^4^*J*_(CCCCF)_ = 3.0 Hz, C1’), 129.30, 128.32 (d, ^3^*J*_(CCCF)_ = 8.4 Hz, C2’, C6’), 123.24, 115.17 (d, ^2^*J*_(CCF)_ = 21.8 Hz, C3’, C6’), 115.71, 114.16, 74.67 (d, ^3^*J*_(CCCP)_ = 7.8 Hz, C5), 63.81 (d, ^1^*J*_(CP)_ = 163.9 Hz, C3), 63.25 (d, ^2^*J*_(COP)_ = 6.4 Hz, CH*_2_*OP), 62.49 (d, ^2^*J*_(COP)_ = 6.7 Hz, CH*_2_*OP), 46.80 (CH_2_N), 46.30 (d, ^3^*J*_(CNCP)_ = 2.0 Hz, CH_3_N), 44.32 (*C*H_2_Ph), 36.16 (C4), 16.51 (d, ^3^*J*_(CCOP)_ = 5.5 Hz, *C*H_3_CH_2_OP), 16.4 (d, ^3^*J*_(CCOP)_ = 5.6 Hz, *C*H_3_CH_2_OP). ^31^P NMR (243 MHz, CDCl_3_): δ = 21.70. Anal.calcd. for C_24_H_29_FN_3_O_6_P × 1.5 H_2_O: C, 54.13; H, 6.06; N, 7.89. Found: C, 54.32; H, 5.90; N, 7.73 (obtained on 10:90 mixtures of *cis*-**20d** and *trans*-**20d**).

**Diethyl *trans*-**(**2-benzyl-5-**((**1-benzyl-2,4-dioxo-1,4-dihydroquinazolin-3**(**2*H***)**-yl**)**methyl**)**isoxazolidin-3-yl**)**phosphonate** (***trans*-21a**)**.** Colorless oil. IR (film, cm^–1^) ν_max_: 2979, 1710, 1657, 1607, 1483, 1404, 1231, 1054, 1020, 967, 764. NMR signals of *trans*-**21a** were extracted from the spectra of 10:90 mixtures of *cis*-**21a** and *trans*-**21a**. ^1^H NMR (600 MHz, CDCl_3_): δ = 8.25 (dd, *J* = 7.9 Hz, *J* = 1.4 Hz, 1H), 7.56–7.53 (m, 1H), 7.43–7.39 (m, 2H), 7.33–7.31 (m, 2H), 7.29–7.21 (m, 7H), 7.11 (d, *J* = 8.5 Hz, 1H), 5.40 (AB, *J*_AB_ = 16.3 Hz, 1H, *H*CHN), 5.34 (AB, *J*_AB_ = 16.3 Hz, 1H, HC*H*N), 4.57–4.53 (m, 1H, *H*C5), 4.49 (dd, ^2^*J* = 12.9 Hz, ^3^*J*_(HC–H5)_ = 5.5 Hz, 1H, *H*CHN), 4.46 (d, ^2^*J* = 13.9 Hz, 1H, HC*H*Ph), 4.26–4.18 (m, 5H, 2 × C*H_2_*OP, HC*H*N), 4.09 (d, ^2^*J* = 13.9 Hz, 1H, *H*CHPh), 3.42 (ddd, ^3^*J*_(H3–H4β)_ = 9.2 Hz, ^3^*J*_(H3–H4α)_ = 7.3 Hz, ^2^*J*_(H3–P)_ = 3.0 Hz, 1H, *H*C3), 2.69 (dddd, ^3^*J*_(H4α–P)_ = 17.3 Hz, ^2^*J*_(H4α–H4β)_ = 12.6 Hz, ^3^*J*_(H4α–H3)_ = 7.3 Hz, ^3^*J*_(H4β–H5)_ = 7.3 Hz, 1H, *H*αC4), 2.42 (dddd, ^2^*J*_(H4β–H4α)_ = 12.6 Hz, ^3^*J*_(H4β–P)_ = 12.6 Hz, ^3^*J*_(H4β–H3)_ = 9.2 Hz, ^3^*J*_(H4α–H5)_ = 7.3 Hz, 1H, *H*βC4), 1.35 (t, ^3^*J* = 7.0 Hz, 3H, C*H_3_*CH_2_OP), 1.34 (t, ^3^*J* = 7.0 Hz, 3H, C*H_3_*CH_2_OP). ^13^C NMR (150 MHz, CDCl_3_): δ = 161.73 (C(O)), 151.35 (C(O)), 139.98, 137.17, 135.65, 135.11, 129.56, 128.98, 128.07, 127.63, 127.63, 127.18, 126.43, 123.10, 115.71, 114.42, 74.47 (d, ^3^*J*_(CCCP)_ = 7.4 Hz, C5), 63.25 (d, ^2^*J*_(COP)_ = 6.5 Hz, CH*_2_*OP), 62.96 (d, ^3^*J*_(CNCP)_ = 5.5 Hz, *C*H_2_Ph), 62.44 (d, ^2^*J*_(COP)_ = 6.8 Hz, CH*_2_*OP), 60.90 (d, ^1^*J*_(CP)_ = 170.3 Hz, C3), 47.43 (CH_2_N), 44.69 (*C*H_2_Ph), 35.49 (C4), 16.53 (d, ^3^*J*_(CCOP)_ = 5.6 Hz, *C*H_3_CH_2_OP), 16.50 (d, ^3^*J*_(CCOP)_ = 5.7 Hz, *C*H_3_CH_2_OP). ^31^P NMR (243 MHz, CDCl_3_): δ = 22.13. Anal.calcd. for C_30_H_34_N_3_O_6_P: C, 63.93; H, 6.08; N, 7.46. Found: C, 63.66; H, 6.38; N, 7.20 (obtained on 10:90 mixtures of *cis*-**21a** and *trans*-**21a**).

**Diethyl *trans*-**(**2-benzyl-5-**((**1-**(**2-fluorobenzyl**)**-2,4-dioxo-1,2-dihydroquinazolin-3**(**2*H***)**-yl**)**methyl**)**isoxazolidin-3-yl**)**phosphonate** (***trans*-21b**)**.** Colorless oil. IR (film, cm^–1^) ν_max_: 2981, 1710, 1657, 1607, 1482, 1354, 1246, 1054, 1020, 967, 764. NMR signals of *trans*-**21b** were extracted from the spectra of 15:85 mixtures of *cis*-**21b** and *trans*-**21b**. ^1^H NMR (600 MHz, CDCl_3_): δ = 8.26 (dd, *J* = 7.9 Hz, *J* = 1.5 Hz, 1H), 7.60–7.57 (m, 1H), 7.42–7.38 (m, 2H), 7.29–7.21 (m, 5H), 7.14–7.10 (m, 2H), 7.07–7.05 (m, 1H), 7.03–7.00 (m, 1H), 5.45 (AB, *J*_AB_ = 16.9 Hz, 1H, *H*CHN), 5.41 (AB, *J*_AB_ = 16.9 Hz, 1H, HC*H*N), 4.56 (d, ^2^*J* = 13.9 Hz, 1H, *H*CHPh), 4.50 (dd, ^2^*J* = 12.8 Hz, ^3^*J*_(HC–H5)_ = 7.5 Hz, 1H, *H*CHN), 4.40–4.39 (m, 1H, *H*C5), 4.26–4.18 (m, 5H, 2 × C*H_2_*OP, HC*H*N), 4.08 (d, ^2^*J* = 13.9 Hz, 1H, HC*H*Ph), 3.42 (ddd, ^3^*J*_(H3–H4β)_ = 9.1 Hz, ^3^*J*_(H3–H4α)_ = 7.2 Hz, ^2^*J*_(H3–P)_ = 3.0 Hz, 1H, *H*C3), 2.69 (dddd, ^3^*J*_(H4α–P)_ = 19.7 Hz, ^2^*J*_(H4α–H4β)_ = 12.8 Hz, ^3^*J*_(H4α–H3)_ = 7.2 Hz, ^3^*J*_(H4β–H5)_ = 7.2 Hz, 1H, *H*αC4), 2.42 (dddd, ^3^*J*_(H4β–P)_ = 12.8 Hz, ^2^*J*_(H4β–H4α)_ = 12.8 Hz, ^3^*J*_(H4β–H3)_ = 9.1 Hz, ^3^*J*_(H4β–H5)_ = 6.5 Hz, 1H, *H*βC4), 1.35 (t, ^3^*J* = 7.1 Hz, 3H, C*H_3_*CH_2_OP), 1.34 (t, ^3^*J* = 7.1 Hz, 3H, C*H_3_*CH_2_OP). ^13^C NMR (150 MHz, CDCl_3_): δ = 161.66 (C(O)), 160.29 (d, ^1^*J*_(CF)_ = 245.6 Hz, C2’), 151.40 (C(O)), 139.66, 136.87, 135.30, 129.63, 129.35 (d, ^3^*J*_(CCCF)_ = 8.3 Hz, C6’), 129.18, 128.08, 128.05 (d, ^3^*J*_(CCCF)_ = 3.5 Hz, C4’), 127.26, 124.75 (d, ^4^*J*_(CCCCF)_ = 3.3 Hz, C5’), 123.27 122.67 (d, ^2^*J*_(CCF)_ = 14.0 Hz, C3’), 115.70, 115.57 (d, ^2^*J*_(CCF)_ = 21.5 Hz, C1’), 113.98, 74.48 (d, ^3^*J*_(CCCP)_ = 7.3 Hz, C5), 63.32 (d, ^2^*J*_(COP)_ = 6.5 Hz, CH*_2_*OP), 62.80 (d, ^3^*J*_(CNCP)_ = 5.4 Hz, *C*H_2_Ph), 62.47 (d, ^2^*J*_(COP)_ = 6.9 Hz, CH*_2_*OP), 60.79 (d, ^1^*J*_(CP)_ = 169.6 Hz, C3), 44.68 (CH_2_N), 41.08 (d, ^3^*J*_(CCCF)_ = 5.3 Hz, *C*H_2_Ph), 35.43 (d, ^2^*J*_(CCP)_ = 1.6 Hz, C4), 16.53 (d, ^3^*J*_(CCOP)_ = 5.6 Hz, *C*H_3_CH_2_OP), 16.49 (d, ^3^*J*_(CCOP)_ = 5.8 Hz, *C*H_3_CH_2_OP). ^31^P NMR (243 MHz, CDCl_3_): δ = 22.12. Anal.calcd. for C_30_H_33_FN_3_O_6_P: C, 61.96; H, 5.72; N, 7.23. Found: C, 61.86; H, 5.57; N, 7.35 (obtained on 15:85 mixtures of *cis*-**21b** and *trans*-**21b**).

**Diethyl *trans*-**(**2-benzyl-5-**((**1-**(**3-fluorobenzyl**)**-2,4-dioxo-1,2-dihydroquinazolin-3**(**2*H***)**-yl**)**methyl**)**isoxazolidin-3-yl**)**phosphonate** (***trans*-21c**)**.** Colorless oil. IR (film, cm^–1^) ν_max_: 2978, 1704, 1656, 1607, 1483, 1351 1246, 1053, 1022, 968, 765. NMR signals of *trans*-**21c** were extracted from the spectra of 15:85 mixtures of *cis*-**21c** and *trans*-**21c**. ^1^H NMR (600 MHz, CDCl_3_): δ = 8.13 (dd, *J* = 7.9 Hz, *J* = 1.5 Hz, 1H), 7.58 (ddd, *J* = 8.6 Hz, *J* = 7.3 Hz, *J* = 1.6 Hz, 1H), 7.42–7.38 (m, 2H), 7.31–7.21 (m, 5H), 7.07 (d, *J* = 8.6 Hz, 1H), 7.03 (d, *J* = 7.3 Hz, 1H), 7.00–6.94 (m, 2H), 5.39 (AB, *J*_AB_ = 16.7 Hz, 1H, *H*CHN), 5.33 (AB, *J*_AB_ = 16.7 Hz, 1H, HC*H*N), 4.56–4.47 (m, 1H, *H*C5), 4.50 (dd, ^2^*J* = 13.8 Hz, ^3^*J*_(HC–H5)_ = 5.3 Hz, 1H, *H*CHN), 4.45 (d, ^2^*J* = 13.8 Hz, 1H, *H*CHPh), 4.26–4.17 (m, 5H, 2 × C*H_2_*OP, HC*H*N), 4.08 (d, ^2^*J* = 13.8 Hz, 1H, HC*H*Ph), 3.41 (ddd, ^3^*J*_(H3–H4β)_ = 9.2 Hz, ^3^*J*_(H3–H4α)_ = 7.1 Hz, ^2^*J*_(H3–P)_ = 3.1 Hz, 1H, *H*C3), 2.69 (dddd, ^3^*J*_(H4α–P)_ = 17.3 Hz, ^2^*J*_(H4α–H4β)_ = 12.9 Hz, ^3^*J*_(H4α–H3)_ = 7.1 Hz, ^3^*J*_(H4β–H5)_ = 7.1 Hz, 1H, *H*αC4), 2.42 (dddd, ^2^*J*_(H4β–H4α)_ = 12.9 Hz, ^3^*J*_(H4β–P)_ = 12.9 Hz, ^3^*J*_(H4β–H3)_ = 9.2 Hz, ^3^*J*_(H4β–H5)_ = 6.6 Hz, 1H, *H*βC4), 1.36 (t, ^3^*J* = 7.1 Hz, 3H, C*H_3_*CH_2_OP), 1.35 (t, ^3^*J* = 7.1 Hz, 3H, C*H_3_*CH_2_OP). ^13^C NMR (150 MHz, CDCl_3_): δ = 163.20 (d, ^1^*J*_(CF)_ = 247.6 Hz, C3’), 161.61 (C(O)), 151.30 (C(O)), 139.78, 138.30 (d, ^3^*J*_(CCCF)_ = 6.8 Hz, C5’), 137.11, 135.20, 130.65 (d, ^3^*J*_(CCCF)_ = 8.0 Hz, C1’), 129.56, 129.25, 128.06, 127.19, 123.29, 122.01 (d, ^4^*J*_(CCCCF)_ = 2.8 Hz, C6’), 115.75, 115.70 (d, ^2^*J*_(CCF)_ = 21.0 Hz, C2’), 114.14, 113.57 (d, ^2^*J*_(CCF)_ = 22.4 Hz, C4’), 75.45 (d, ^3^*J*_(CCCP)_ = 7.1 Hz, C5), 63.25 (d, ^2^*J*_(COP)_ = 6.5 Hz, CH*_2_*OP), 62.94 (d, ^3^*J*_(CNCP)_ = 5.7 Hz, *C*H_2_Ph), 62.45 (d, ^2^*J*_(COP)_ = 6.8 Hz, CH*_2_*OP), 60.87 (d, ^1^*J*_(CP)_ = 169.8 Hz, C3), 47.01 (CH_2_N), 44.73 (*C*H_2_Ph), 35.17 (d, ^2^*J*_(CCP)_ = 1.7 Hz, C4), 16.51 (d, ^3^*J*_(CCOP)_ = 5.7 Hz, *C*H_3_CH_2_OP), 16.48 (d, ^3^*J*_(CCOP)_ = 5.6 Hz, *C*H_3_CH_2_OP). ^31^P NMR (243 MHz, CDCl_3_): δ = 22.09. Anal.calcd. for C_30_H_33_FN_3_O_6_P: C, 61.69; H, 5.72; N, 7.23. Found: C, 61.97; H, 5.89; N, 7.11 (obtained on 10:90 mixtures of *cis*-**21c** and *trans*-**21c**).

**Diethyl *trans*-**(**2-benzyl-5-**((**1-**(**4-fluorobenzyl**)**-2,4-dioxo-1,2-dihydroquinazolin-3**(**2*H***)**-yl**)**methyl**)**isoxazolidin-3-yl**)**phosphonate** (***trans*-21d**)**.** Colorless oil. IR (film, cm^–1^) ν_max_: 2978, 1710, 1657, 1607, 1482, 1353, 1243, 1054, 1020, 967, 764. NMR signals of *trans*-**21d** were extracted from the spectra of 15:85 mixtures of *cis*-**21d** and *trans*-**21d**. ^1^H NMR (600 MHz, CDCl_3_): δ = 8.24 (dd, *J* = 7.8 Hz, *J* = 1.5 Hz, 1H), 7.59–7.57 (m, 1H), 7.42–7.38 (m, 2H), 7.27–7.22 (m, 6H), 7.10 (d, *J* = 8.5 Hz, 1H), 7.03–6.99 (m, 2H), 5.38 (AB, *J*_AB_ = 15.1 Hz, 1H, *H*CHN), 5.30 (AB, *J*_AB_ = 15.1 Hz, 1H, HC*H*N), 4.56–4.48 (m, 2H, *H*C5, *H*CHN), 4.46 (d, ^2^*J* = 13.8 Hz, 1H, *H*CHPh), 4.26–4.18 (m, 5H, 2 × C*H_2_*OP, HC*H*N), 4.08 (d, ^2^*J* = 13.8 Hz, 1H, HC*H*Ph), 3.41 (ddd, ^3^*J*_(H3–H4β)_ = 9.2 Hz, ^3^*J*_(H3–H4α)_ = 7.1 Hz, ^2^*J*_(H3–P)_ = 3.1 Hz, 1H, *H*C3), 2.70 (dddd, ^3^*J*_(H4α–P)_ = 19.6 Hz, ^2^*J*_(H4α–H4β)_ = 12.07 Hz, ^3^*J*_(H4α–H3)_ = 7.1 Hz, ^3^*J*_(H4β–H5)_ = 7.1 Hz, 1H, *H*αC4), 2.42 (dddd, ^2^*J*_(H4β–H4α)_ = 12.7 Hz, ^3^*J*_(H4β–P)_ = 12.7 Hz, ^3^*J*_(H4β–H3)_ = 9.2 Hz, ^3^*J*_(H4β–H5)_ = 6.3 Hz, 1H, *H*βC4), 1.37 (t, ^3^*J* = 7.0 Hz, 3H, C*H_3_*CH_2_OP), 1.36 (t, ^3^*J* = 7.0 Hz, 3H, C*H_3_*CH_2_OP). ^13^C NMR (150 MHz, CDCl_3_): δ = 162.21 (d, ^1^*J*_(CF)_ = 246.5 Hz, C4’), 161.63 (C(O)), 151.31 (C(O)), 139.82, 137.18, 135.13, 131.37 (d, ^4^*J*_(CCCCF)_ = 3.0 Hz, C1’), 129.52, 129.23, 128.85, 128.25 (d, ^3^*J*_(CCCF)_ = 7.9 Hz, C2’, C6’), 128.06, 127.99, 127.18, 123.21, 115.93 (d, ^2^*J*_(CCF)_ = 21.3 Hz, C3’, C5’), 115.77, 114.17, 74.42 (d, ^3^*J*_(CCCP)_ = 7.0 Hz, C5), 63.23 (d, ^2^*J*_(COP)_ = 6.5 Hz, CH*_2_*OP), 62.95 (d, ^3^*J*_(CNCP)_ = 5.5 Hz, *C*H_2_Ph), 62.45 (d, ^2^*J*_(COP)_ = 6.8 Hz, CH*_2_*OP), 60.92 (d, ^1^*J*_(CP)_ = 169.8 Hz, C3), 46.79 (CH_2_N), 44.75 (*C*H_2_Ph), 35.50 (d, ^2^*J*_(CCP)_ = 1.8 Hz, C4), 16.52 (d, ^3^*J*_(CCOP)_ = 5.4 Hz, *C*H_3_CH_2_OP), 16.48 (d, ^3^*J*_(CCOP)_ = 5.4 Hz, *C*H_3_CH_2_OP). ^31^P NMR (243 MHz, CDCl_3_): δ = 22.09. Anal.calcd. for C_30_H_33_FN_3_O_6_P: C, 61.96; H, 5.72; N, 7.23. Found: C, 62.26; H, 5.80; N, 7.00 (obtained on 10:90 mixtures of *cis*-**21d** and *trans*-**21d**).

### 3.6. Antiviral Activity Assays

The compounds were evaluated against different herpesviruses, including herpes simplex virus type 1 (HSV-1) strain KOS, thymidine kinase-deficient (TK^–^) HSV-1 KOS strain resistant to ACV (ACV^r^), herpes simplex virus type 2 (HSV-2) strain G, varicella-zoster virus (VZV) strain Oka, TK^–^ VZV strain 07-1, and human cytomegalovirus (HCMV) strains AD-169 and Davis as well as vaccinia virus, adenovirus-2, human coronavirus, parainfluenza-3 virus, reovirus-1, Sindbis virus, Coxsackie virus B4, Punta Toro virus, respiratory syncytial virus (RSV), and influenza A virus subtypes H1N1 (A/PR/8), H3N2 (A/HK/7/87), and influenza B virus (B/HK/5/72), based on inhibition of virus-induced cytopathicity or plaque formation in human embryonic lung (HEL) fibroblasts, African green monkey kidney cells (Vero), human epithelial cervix carcinoma cells (HeLa), or Madin–Darby canine kidney cells (MDCK). Confluent cell cultures in microtiter 96-well plates were inoculated with 100 CCID_50_ of virus (1 CCID_50_ being the virus dose to infect 50% of the cell cultures) or with 20 plaque forming units (PFU), and the cell cultures were incubated in the presence of varying concentrations of the test compounds. Viral cytopathicity or plaque formation (VZV) was recorded as soon as it reached completion in the control virus-infected cell cultures that were not treated with the test compounds. Antiviral activity was expressed as the EC_50_ or compound concentration required to reduce virus-induced cytopathicity or viral plaque formation by 50%. Cytotoxicity of the test compounds was expressed as the minimum cytotoxic concentration (MCC) or the compound concentration that caused a microscopically detectable alteration of cell morphology.

### 3.7. Antibacterial Activity Assays

The antimicrobial tests were performed using reference strains of microbia from the American Type Culture Collection (ATCC), including *E. faecalis* ATCC 29212, *S. aureus* ATCC 2593, *E. coli* ATCC 25922, *P. aeruginosa* ATCC 27853, and two fungal strains, *C. albicans* ATCC 10241 and *A. brasiliensis* ATCC 16404. From the Polish Collection of Microorganisms (PCM), *B. cereus* PCM 1948 was used. The antimicrobial activity of the compounds was assessed according to their minimal inhibitory concentrations (MIC) and minimal bactericidal concentrations (MBC). The MIC and MBC were expressed in mg/mL. Antibacterial and antifungal activities were determined using the broth microdilution method in a liquid medium according to The European Committee on Antimicrobial Susceptibility (EUCAST) recommendations. The Mueller–Hinton liquid medium (pH~7.2) (BioMerieux, Marcy L′Etoile, France) was used for bacteria. Liquid medium RPMI-1640 (pH~7.2) (Sigma, Darmstadt, Germany) was used for the fungal strains. Each tested compound was dissolved in 10 mg/mL in sterile water. Two-fold series dilutions of the different compounds in the growth medium were performed in the 96-well sterile microtiter plates (Kartell Labware, Noviglio, Italy). Inocula were freshly prepared and standardized as microbial suspensions (McFarland scale) containing 10^8^ colony forming units (cfu/mL), added at a volume of 10 μL, to the wells of the microtiter plate together with the serial dilutions of the compounds in the growth medium. After 24 h of incubation at 37 °C, microbial growth was evaluated spectrophotometrically at 595 nm using a Microplate reader 680 (BioRad, Hercules, CA, USA). The lowest concentration of the tested compounds resulting in total growth inhibition was taken as the MIC value. To determine the MBC, 10 μL of the culture were collected from each well, where no visible growth of microorganisms was recorded and plated onto the surface of Brain Heart Infusion Agar (BioMerieux, Marcy L′Etoile, France). The cultures were incubated for 24 h at 37 °C. An absence of microbial growth indicated bactericidal activity by the tested compounds. Plates with *A. brasiliensis* were incubated at 37 °C for three days. The tests were performed in two independent experiments. Amikacin (Sigma, Darmstadt, Germany) and fluconazole (Sigma) were used as antimicrobial standards.

### 3.8. Microbial Mutagenicity Assay—The Ames Test

Mutagenicity was determined using a standard microplate AMES MPF^TM^ PENTA I kit according to the manufacturer’s instructions (Xenometrix, Allschwil, Switzerland) [29]. Bacteria were exposed to 25 µL of the tested compound (0.625 mg/mL) as well as positive and negative controls for 90 min in a medium-containing sufficient histidine (*S. typhimurium*) or tryptophan (*E. coli*) to support approximately two cell divisions. After exposure, the cultures were diluted in pH indicator medium lacking histidine or tryptophan and aliquoted into 48 wells of a 384-well plate. Within two days, cells that had undergone reversion to amino acid prototrophy grew. Bacterial metabolism reduces the pH of the medium, changing the color of that well. The number of wells containing revertant colonies was counted for each dose and compared to a solvent (negative) control. Each dose was performed in triplicate to allow for statistical analysis of the data. A dose-dependent increase in the number of revertant colonies upon exposure to the test sample relative to the solvent control indicates that the sample is mutagenic in the Ames MPF assay. The mutagenic potential of the sample was assessed after metabolic activation in the presence of Aroclor 1254-induced rat liver S9 (S9 Cofactor kit, Xenometrix, Allschwil, Switzerland). The following mutagenic compounds were used as positive controls: 2-aminoanthracene (for *S. typhimurium* TA98, TA100, TA1535 and TA1537) and 2-aminofluorene (for *E.coli* strain WP2 uvrA[pKM101]). As the negative control, 50% DMSO was used.

## 4. Conclusions

Good yields (61–96%) and *trans*/*cis* diastereoselectivities (d.e = 70–84%) for the functionalized diastereoisomeric 3-(diethoxyphosphonyl)isoxazolidines *trans*-**20**/*cis*-**20** and *trans*-**21**/*cis*-**21** were observed in 1,3-dipolar cycloadditions of *N*-methyl- and *N*-benzyl-C-(diethoxyphosphonyl)nitrones **22** and **23** with selected *N*^3^-allyl-*N*^1^-benzylquinazoline-2,4-diones **24a–d**. The inhibitory activities of alkenes **24a–d** and isoxazolidines **20** and **21** were assayed toward a broad panel of DNA and RNA viruses and several isoxazolidines appeared active toward VZV (EC_50_ = 12.63–58.48 µM). The inseparable mixtures of isoxazolidienes *cis*-**21b/***trans*-**21b** (15:85) (EC_50_ = 12.63 µM) and cis-**21a**/trans-**21a** (10:90) (EC_50_ = 14.5 µM) showed the highest activity against TK^+^ VZV OKA strain, but much lower than that of the reference compounds acyclovir (EC_50_ = 0.49 µM) and brivudine (EC_50_ = 0.026 µM). On the other hand, the mixture of isoxazolidines *cis*-**20c/***trans*-**20c** (6:94) inhibited the growth of *B. cereus* PCM 1948, the bacteria responsible for foodborne illnesses, showing the MIC value of 0.625 mg/mL while not being mutagenic at this concentration. Unfortunately, the observed MIC value was lower than that of the reference drug amikacin (MIC = 0.02 mg/mL).

## Figures and Tables

**Table 1:** Cycloadditions of the nitrone **22**/**23** and *N*^3^-allyl-*N*^1^-benzylquinazoline-2,4-diones **24a–d**.

Nitrone 22/23 (R)	Alkene 24 (R′) 13	*cis:trans* Ratio	Yield (%)
**22** (Me)	Ph	10:90	*cis*-**20a** + *trans*-**20a** (87) ^a^
**22** (Me)	2-F-C_6_H_4_	8:92	*cis*-**20b** + *trans*-**20b** (80) ^a^
**22** (Me)	3-F-C_6_H_4_	6:94	*cis*-**20c** + *trans*-**20c** (96) ^a^
**22** (Me)	4-F-C_6_H_4_	10:90	*cis*-**20d** + *trans*-**20d** (92) ^a^
**23** (Bn)	Ph	10:90	*cis*-**21a** + *trans*-**21a** (95) ^a^
**23** (Bn)	2-F-C_6_H_4_	15:85	*cis*-**21b** + *trans*-**21b** (71) ^a^
**23** (Bn)	3-F-C_6_H_4_	10:90	*cis*-**21c** + *trans*-**21c** (91) ^a^
**23** (Bn)	3-F-C_6_H_4_	15:85	*cis*-**21d** + *trans*-**21d** (84) ^a^

^a^ Yield of pure mixture of *cis*- and *trans*-isomers.

**Table 2:** Antiviral activity and cytotoxicity against varicella-zoster virus (VZV) in HEL cell cultures.

Compound	R	R′	Antiviral Activity EC_50_ (μM) ^a^		Cytotoxicity (μM)
			TK^+^ VZV Strain (OKA)	TK^–^ VZV Strain (07-1)	Cell Morphology MCC ^b^
*cis*-**20a**/*trans*-**20a** (10:90)	Me	Ph	31.68	100	>100
*cis*-**20b**/*trans*-**20b** (8:92)	Me	2-F-C_6_H_4_	31.68	58.48	>100
*cis*-**20c**/*trans*-**20c** (6:94)	Me	3-F-C_6_H_4_	100	58.48	>100
*cis*-**20d**/*trans*-**20d** (10:90)	Me	4-F-C_6_H_4_	58.48	38.07	>100
*cis*-**21a**/*trans*-**21a** (10:90)	Bn	Ph	14.5	34.2	100
*cis*-**21b**/*trans*-**21b** (15:85)	Bn	2-F-C_6_H_4_	12.63	27.59	100
*cis*-**21c**/*trans*-**21c** (10:90)	Bn	3-F-C_6_H_4_	20	14.49	20
*cis*-**21d**/*trans*-**21d** (15:85)	Bn	4-F-C_6_H_4_	20	20	100
**24a**	-	Ph	100	100	>100
**24b**	-	2-F-C_6_H_4_	>100	64.47	>100
**24c**	-	3-F-C_6_H_4_	>100	>100	>100
**24d**	-	4-F-C_6_H_4_	54.69	>20	100
**Acyclovir**			0.49	23.22	>440
**Brivudin**			0.026	12.01	>300

^a^ Effective concentration required to reduce virus plaque formation by 50%. Virus input was 100 plaque forming units (PFU). ^b^ Minimum cytotoxic concentration that causes a microscopically detectable alternation of cell morphology.

**Table 3:** Antimicrobial activity of isoxazolidines *cis*-**20**/*trans*-**20** and *cis*-**2a**/*trans*-**2a**, and alkene **24**.

Compound	*E. faecalis* ATCC 29212	*S. aureus* ATCC 2593	*B. cereus* PCM 1948	*E. coli* ATCC 25922	*P. aeruginosa* ATCC 27853	*C. albicans* ATCC 10241	*A. brasiliensis* ATCC 16404
	MIC ^a^/MBC ^b^ (mg/mL)	MIC ^a^ = MBC ^b^ (mg/mL)					
*cis*-**20a**/*trans*-**20a** (10:90)	>5/>5	2.5	1.25	1.25	2.5	2.5	5
*cis*-**20b**/*trans*-**20b** (8:92)	>5/>5	5	1.25	2.5	2.5	2.5	5
*cis*-**20c**/*trans*-**20c** (6:94)	>5/>5	5	0.625	2.5	2.5	2.5	5
*cis*-**20d**/*trans*-**20d** (10:90)	>5/>5	5	1.25	2.5	2.5	2.5	5
*cis*-**21a**/*trans*-**21a** (10:90)	>5/>5	5	1.25	2.5	2.5	5	5
*cis*-**21b**/*trans*-**21b** (15:85)	>5/>5	5	1.25	2.5	2.5	2.5	5
*cis*-**21c**/*trans*-**21c** (10:90)	>5/>5	5	2.5	1.25	2.5	2.5	5
*cis*-**21d**/*trans*-**21d** (15:85)	>5/>5	5	1.25	1.25	2.5	2.5	5
**24a**	>5/>5	5	5	2.5	2.5	5	5
**24b**	>5/>5	5	2.5	2.5	2.5	5	5
**24c**	>5/>5	5	5	1.25	2.5	2.5	5
**24d**	>5/>5	5	5	2.5	2.5	2.5	5
**Amikacin**	0.000625	0.001	0.02	0.000625	0.02	-	-
**Fluconazole**	-	-	-	-	-	0.005/>0.005	0.005/>0.005

^a^ Minimal inhibitory concentrations. ^b^ Minimal bactericidal concentrations.

## Data Availability

Data are contained within the article.

## Supplementary Materials

The following supporting information can be downloaded at: https://www.mdpi.com/article/10.3390/molecules27196526/s1, Figure S1: ^1^H NMR Spectrum for *trans*-**20a** in CDCl_3_; Figure S2: ^31^P NMR Spectrum for *trans*-**20a** in CDCl_3_; Figure S3: ^13^C NMR Spectrum for *trans*-**20a** in CDCl_3_; Figure S4: ^1^H NMR Spectrum for *trans*-**20b** in CDCl_3_; Figure S5: ^31^P NMR Spectrum for *trans*-**20b** in CDCl_3_; Figure S6: ^13^C NMR Spectrum for *trans*-**20b** in CDCl_3_; Figure S7: ^1^H NMR Spectrum for *trans*-**20c** in CDCl_3_; Figure S8: ^31^P NMR Spectrum for *trans*-**20c** in CDCl_3_; Figure S9: ^13^C NMR Spectrum for *trans*-**20c** in CDCl_3_; Figure S10: ^1^H NMR Spectrum for *trans*-**20d** in CDCl_3_; Figure S11: ^31^P NMR Spectrum for *trans*-**20d** in CDCl_3_; Figure S12: ^13^C NMR Spectrum for *trans*-**20d** in CDCl_3_; Figure S13: ^1^H NMR Spectrum for *trans*-**21a** in CDCl_3_; Figure S14: ^31^P NMR Spectrum for *trans*-**21a** in CDCl_3_; Figure S15: ^13^C NMR Spectrum for *trans*-**21a** in CDCl_3_; Figure S16: ^1^H NMR Spectrum for *trans*-**21b** in CDCl_3_; Figure S17: ^31^P NMR Spectrum for *trans*-**21b** in CDCl_3_; Figure S18: ^13^C NMR Spectrum for *trans*-**21b** in CDCl_3_; Figure S19: ^1^H NMR Spectrum for *trans*-**21c** in CDCl_3_; Figure S20: ^31^P NMR Spectrum for *trans*-**21c** in CDCl_3_; Figure S21: ^13^C NMR Spectrum for *trans*-**21c** in CDCl_3_; Figure S22: ^1^H NMR Spectrum for *trans*-**21d** in CDCl_3_; Figure S23: ^31^P NMR Spectrum for *trans*-**21d** in CDCl_3_; Figure S24: ^13^C NMR Spectrum for *trans*-**21d** in CDCl_3_; Figure S25: ^1^H NMR Spectrum for **24a** in CDCl_3_; Figure S26: ^13^C NMR Spectrum for **24a** in CDCl_3_; Figure S27: ^1^H NMR Spectrum for **24b** in CDCl_3_; Figure S28: ^13^C NMR Spectrum for **24b** in CDCl_3_; Figure S29: ^1^H NMR Spectrum for **24c** in CDCl_3_; Figure S30: ^13^C NMR Spectrum for **24c** in CDCl_3_; Figure S31: ^1^H NMR Spectrum for **24d** in CDCl_3_; Figure S32: ^13^C NMR Spectrum for **24d** in CDCl_3_; Figure S33: ^1^H NMR Spectrum for **26a** in CDCl_3_; Figure S34: ^13^C NMR Spectrum for **26a** in CDCl_3_; Figure S35: ^1^H NMR Spectrum for **26b** in CDCl_3_; Figure S36: ^13^C NMR Spectrum for **26b** in CDCl_3_; Figure S37: ^1^H NMR Spectrum for **26c** in CDCl_3_; Figure S38: ^13^C NMR Spectrum for **26c** in CDCl_3_; Figure S39: ^1^H NMR Spectrum for **26d** in CDCl_3_; Figure S40: ^13^C NMR Spectrum for **26d** in CDCl_3_; Figure S41: ^1^H NMR Spectrum for **27a** in CDCl_3_; Figure S42: ^13^C NMR Spectrum for **27a** in CDCl_3_; Figure S43: ^1^H NMR Spectrum for **27b** in CDCl_3_; Figure S44: ^13^C NMR Spectrum for **27b** in CDCl_3_; Figure S45: ^1^H NMR Spectrum for **27c** in CDCl_3_; Figure S46: ^13^C NMR Spectrum for **27c** in CDCl_3_; Figure S47: ^1^H NMR Spectrum for **27d** in CDCl_3_; Figure S48: ^13^C NMR Spectrum for **27d** in CDCl_3_.

## Sample Availability

Samples are not available.
