# Supplementary material for: Design, Synthesis, Anti-Varicella-Zoster and Antimicrobial Activity of (Isoxazolidin-3-yl)Phosphonate Conjugates of N1-Functionalised Quinazoline-2,4-Diones
Source: Molecules. 2022 Oct 2;27(19):6526. doi: 10.3390/molecules27196526 (PMC9571433; doi:10.3390/molecules27196526)

Design, Synthesis, Anti-Varicella-Zoster, and Antimicrobial Activity of (Isoxazolidin-3-yl)Phosphonate Conjugates of N1-Functionalised Quinazoline-2,4-Diones

Magdalena Łysakowska [1], Iwona E. Głowacka [1], Graciela Andrei [2], Dominique Schols [2], Robert Snoeck [2], Paweł Lisiecki [3], Magdalena Szemraj [3] and Dorota G. Piotrowska [1,*]

[1.] Bioorganic Chemistry Laboratory, Faculty of Pharmacy, Medical University of Lodz, Muszynskiego 1, 90-151 Lodz, Poland; magdalena.lysakowska@umed.lodz.pl (M.Ł.); iwona.glowacka@umed.lodz.pl (I.E.G.)

[2.] KU Leuven Department of Microbiology, Immunology and Transplantation, Rega Institute, Laboratory of Virology and Chemotherapy, Herestraat 49, box 1030, B-3000 Leuven, Belgium; graciela.andrei@kuleuven.be (G.A.); dominique.schols@kuleuven.be (D.S.); robert.snoeck@kuleuven.be (R.S.);

[3.] Department of Pharmaceutical Microbiology and Microbiological Diagnostics, Faculty of Pharmacy, Medical University of Lodz, Muszynskiego 1, 90-151 Łódź, Poland; pawel.lisiecki@umed.lodz.pl (P.L.); magdalena.szemraj@umed.lodz.pl (M.S.)

* Correspondence: dorota.piotrowska@umed.lodz.pl (D.G.P.)

Contents

Figure S1: ^{1}H NMR Spectrum for *trans*-**20a** in $CDCl_3$

Figure S2: ^{31}P NMR Spectrum for *trans*-**20a** in $CDCl_3$

Figure S3: ^{13}C NMR Spectrum for *trans*-**20a** in $CDCl_3$

Figure S4: ^{1}H NMR Spectrum for *trans*-**20b** in $CDCl_3$

Figure S5: ^{31}P NMR Spectrum for *trans*-**20b** in $CDCl_3$

Figure S6: ^{13}C NMR Spectrum for *trans*-**20b** in $CDCl_3$

Figure S7: ^{1}H NMR Spectrum for *trans*-**20c** in $CDCl_3$

Figure S8: ^{31}P NMR Spectrum for *trans*-**20c** in $CDCl_3$

Figure S9: ^{13}C NMR Spectrum for *trans*-**20c** in $CDCl_3$

Figure S10: ^{1}H NMR Spectrum for *trans*-**20d** in $CDCl_3$

Figure S11: ^{31}P NMR Spectrum for *trans*-**20d** in $CDCl_3$

Figure S12: ^{13}C NMR Spectrum for *trans*-**20d** in $CDCl_3$

Figure S13: ^{1}H NMR Spectrum for *trans*-**21a** in $CDCl_3$

Figure S14: ^{31}P NMR Spectrum for *trans*-**21a** in $CDCl_3$

Figure S15: ^{13}C NMR Spectrum for *trans*-**21a** in $CDCl_3$

Figure S16: ^{1}H NMR Spectrum for *trans*-**21b** in $CDCl_3$

Figure S17: ^{31}P NMR Spectrum for *trans*-**21b** in $CDCl_3$

Figure S18: ^{13}C NMR Spectrum for *trans*-**21b** in $CDCl_3$

Figure S19: ^{1}H NMR Spectrum for *trans*-**21c** in $CDCl_3$

Figure S20: ^{31}P NMR Spectrum for *trans*-**21c** in $CDCl_3$

Figure S21: ^{13}C NMR Spectrum for *trans*-**21c** in $CDCl_3$

Figure S22: ^{1}H NMR Spectrum for *trans*-**21d** in $CDCl_3$

Figure S23: ^{31}P NMR Spectrum for *trans*-**21d** in $CDCl_3$

Figure S24: ^{13}C NMR Spectrum for *trans*-**21d** in $CDCl_3$

Figure S25: ^{1}H NMR Spectrum for **24a** in $CDCl_3$

Figure S26: ^{13}C NMR Spectrum for **24a** in $CDCl_3$

Figure S27: ^{1}H NMR Spectrum for **24b** in $CDCl_3$

Figure S28: ^{13}C NMR Spectrum for **24b** in $CDCl_3$

Figure S29: ^{1}H NMR Spectrum for **24c** in $CDCl_3$

Figure S30: ^{13}C NMR Spectrum for **24c** in $CDCl_3$

Figure S31: ^{1}H NMR Spectrum for **24d** in $CDCl_3$

Figure S32: ^{13}C NMR Spectrum for **24d** in $CDCl_3$

Figure S33: ^{1}H NMR Spectrum for **26a** in $CDCl_3$

Figure S34: ^{13}C NMR Spectrum for **26a** in $CDCl_3$

Figure S35: ^{1}H NMR Spectrum for **26b** in $CDCl_3$

Figure S36: ^{13}C NMR Spectrum for **26b** in $CDCl_3$

Figure S37: ^{1}H NMR Spectrum for **26c** in $CDCl_3$

Figure S38: ^{13}C NMR Spectrum for **26c** in $CDCl_3$

Figure S39: ^{1}H NMR Spectrum for **26d** in $CDCl_3$

Figure S40: ^{13}C NMR Spectrum for **26d** in $CDCl_3$

Figure S41: ^{1}H NMR Spectrum for **27a** in $CDCl_3$

Figure S42: ^{13}C NMR Spectrum for **27a** in $CDCl_3$

Figure S43: ^{1}H NMR Spectrum for **27b** in $CDCl_3$

Figure S44: ^{13}C NMR Spectrum for **27b** in $CDCl_3$

Figure S45: ^{1}H NMR Spectrum for **27c** in $CDCl_3$

Figure S46: ^{13}C NMR Spectrum for **27c** in $CDCl_3$

Figure S47: ^{1}H NMR Spectrum for **27d** in $CDCl_3$

Figure S48: ^{13}C NMR Spectrum for **27d** in $CDCl_3$

Figure S1: ^{1}H NMR Spectrum for *trans*-**20a** in $CDCl_3$

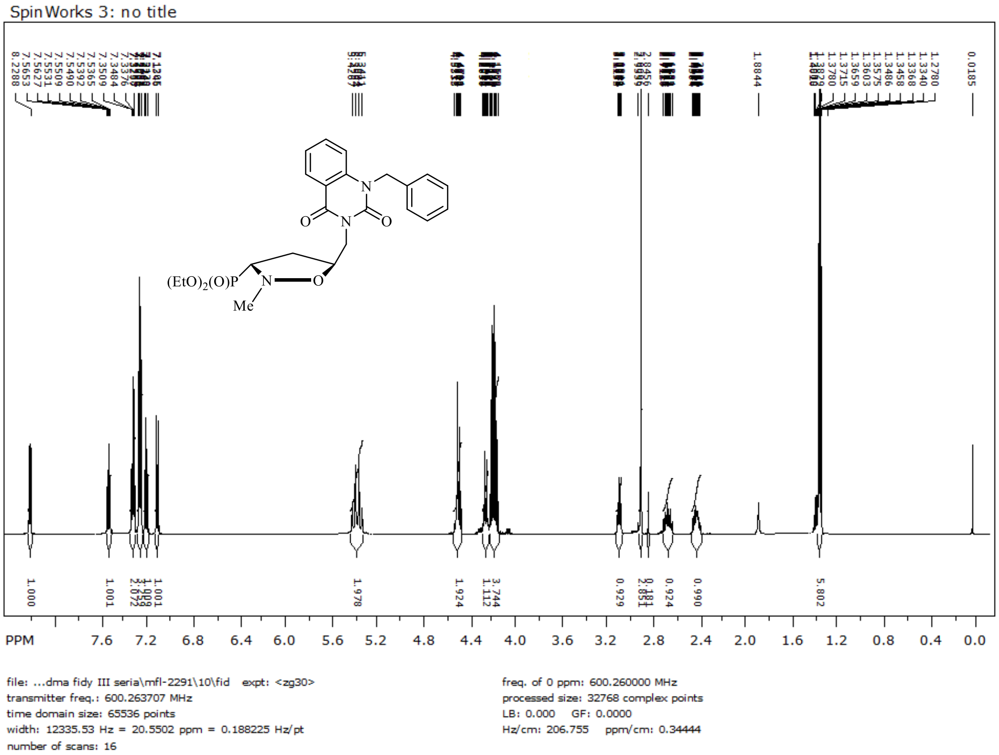

Figure S2: ^{31}P NMR Spectrum for *trans*-**20a** in $CDCl_3$

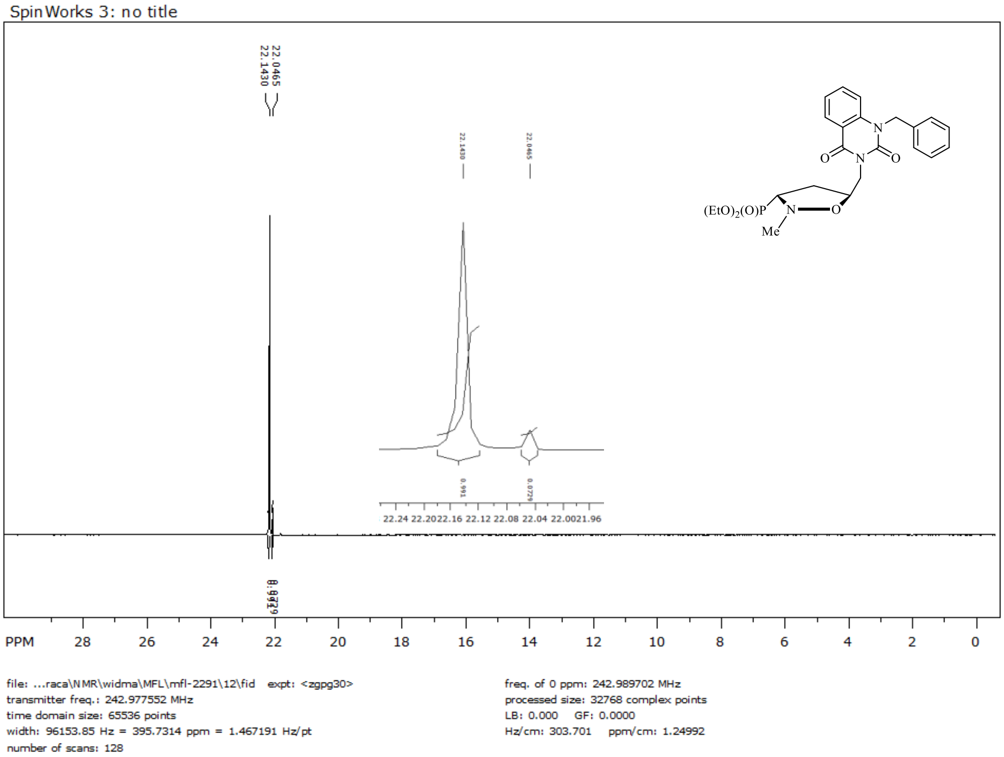

Figure S3: ^{13}C NMR Spectrum for *trans*-**20a** in $CDCl_3$

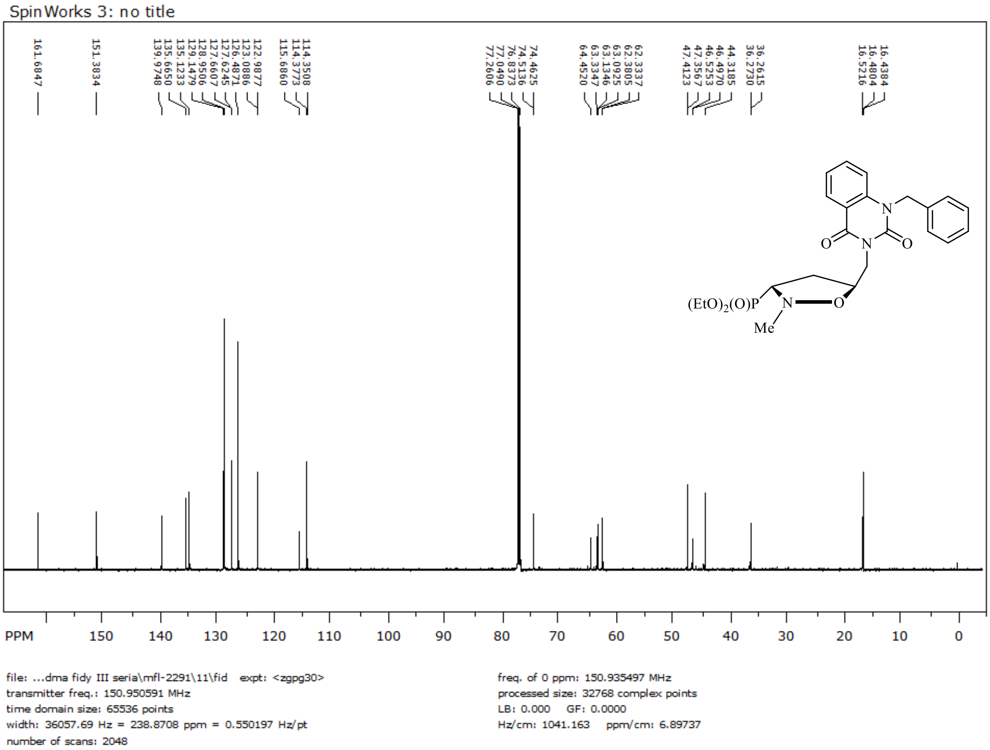

Figure S4: ^{1}H NMR Spectrum for *trans*-**20b** in $CDCl_3$

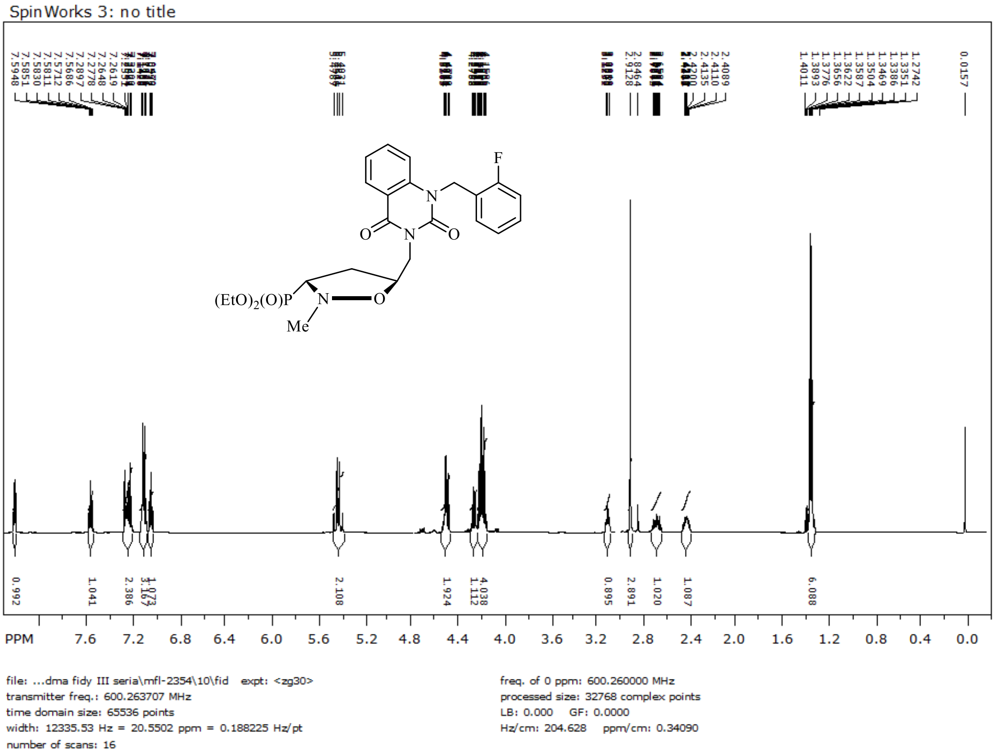

Figure S5: ^{31}P NMR Spectrum for *trans*-**20b** in $CDCl_3$

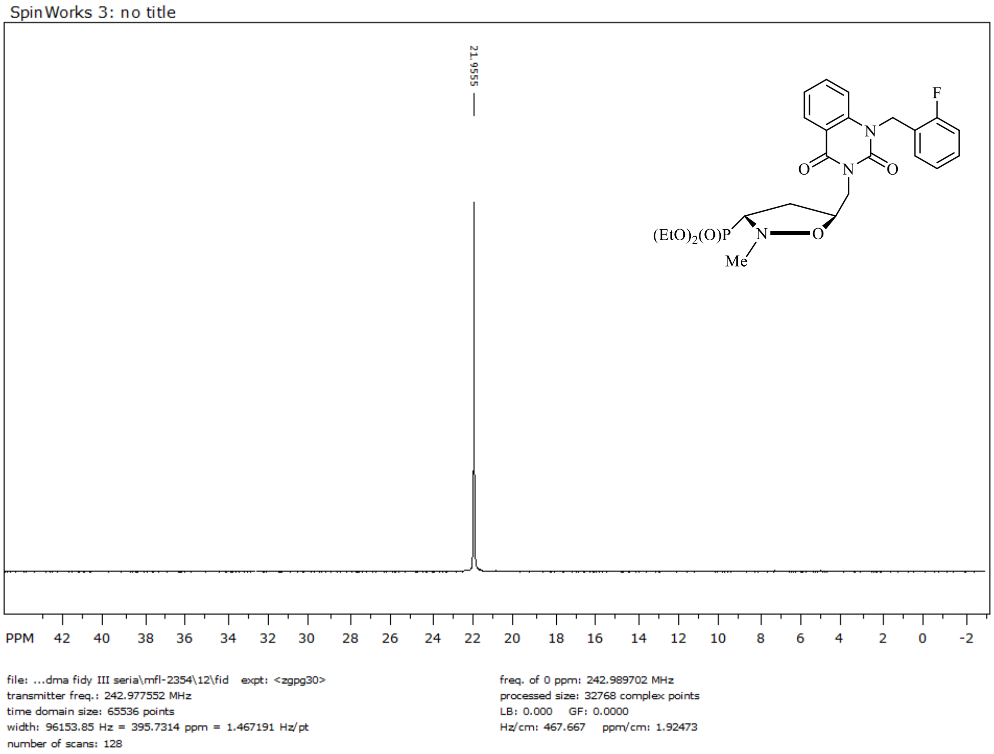

Figure S6: ^{13}C NMR Spectrum for *trans*-**20b** in $CDCl_3$

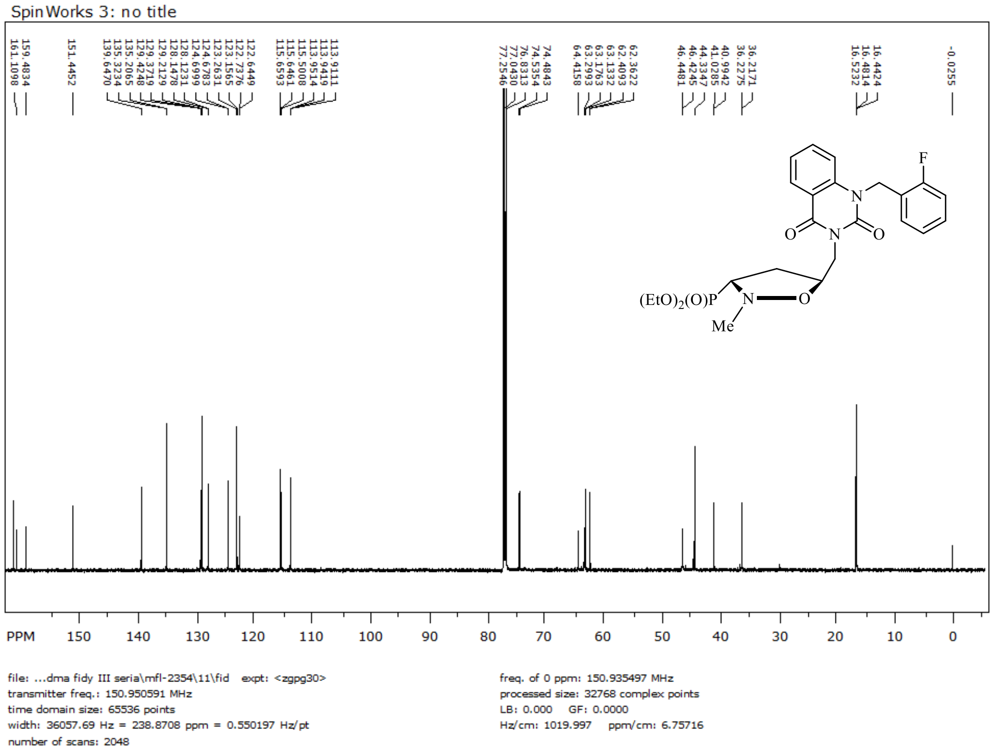

Figure S7: ^{1}H NMR Spectrum for *trans*-**20c** in $CDCl_3$

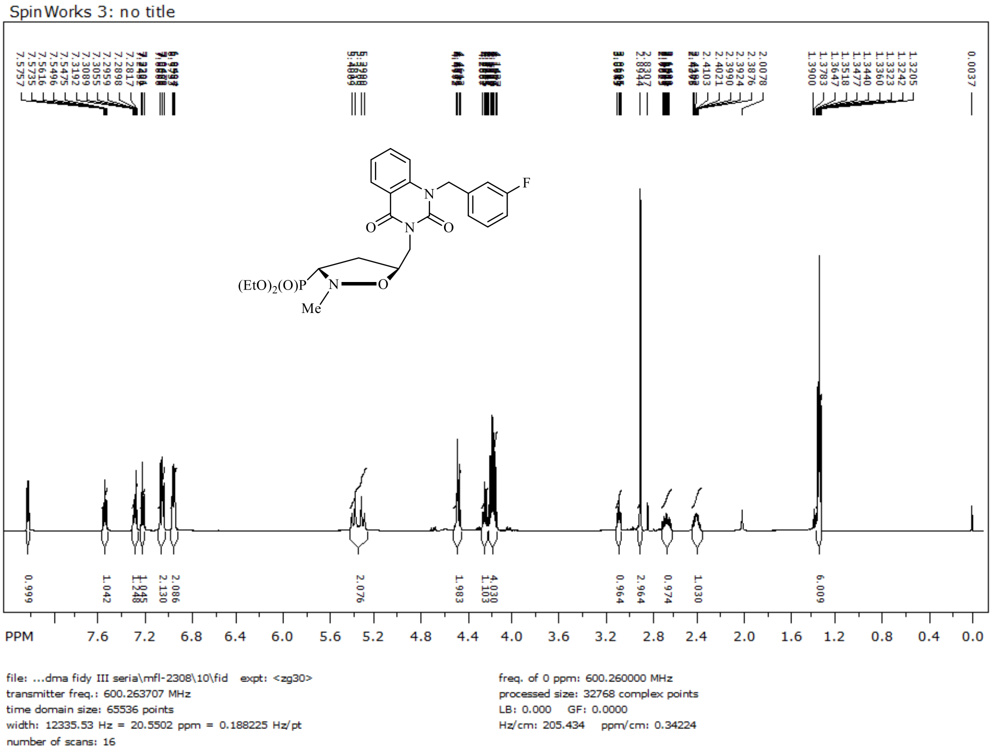

Figure S8: ^{31}P NMR Spectrum for *trans*-**20c** in $CDCl_3$

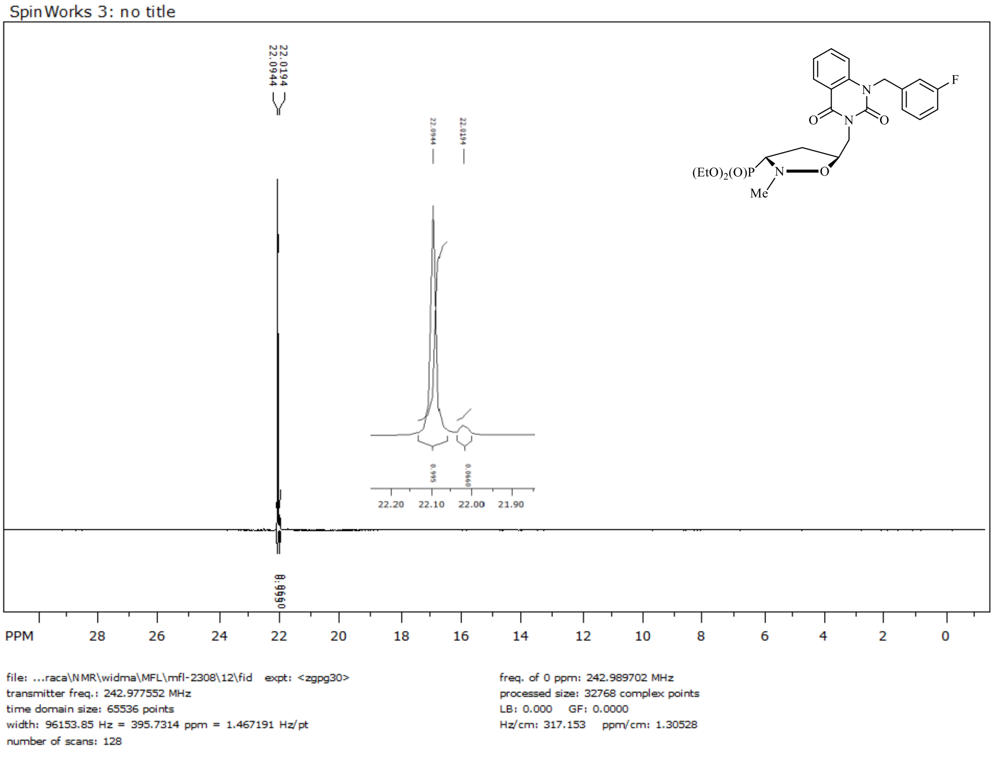

Figure S9: ^{13}C NMR Spectrum for *trans*-**20c** in $CDCl_3$

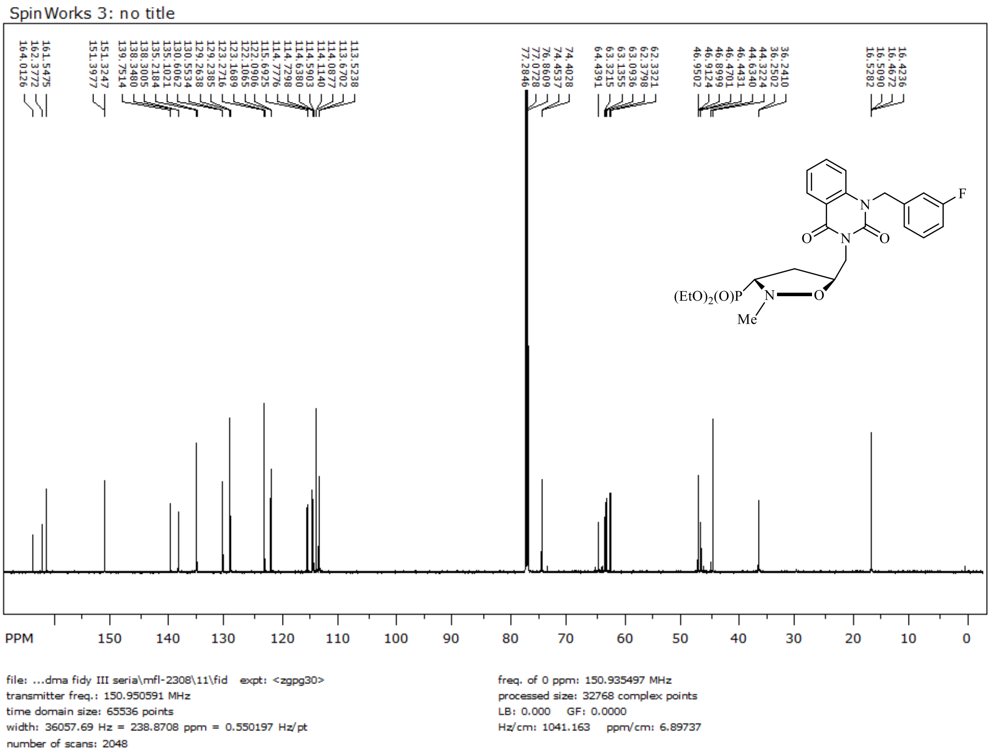

Figure S10: ^{1}H NMR Spectrum for *trans*-**20d** in $CDCl_3$

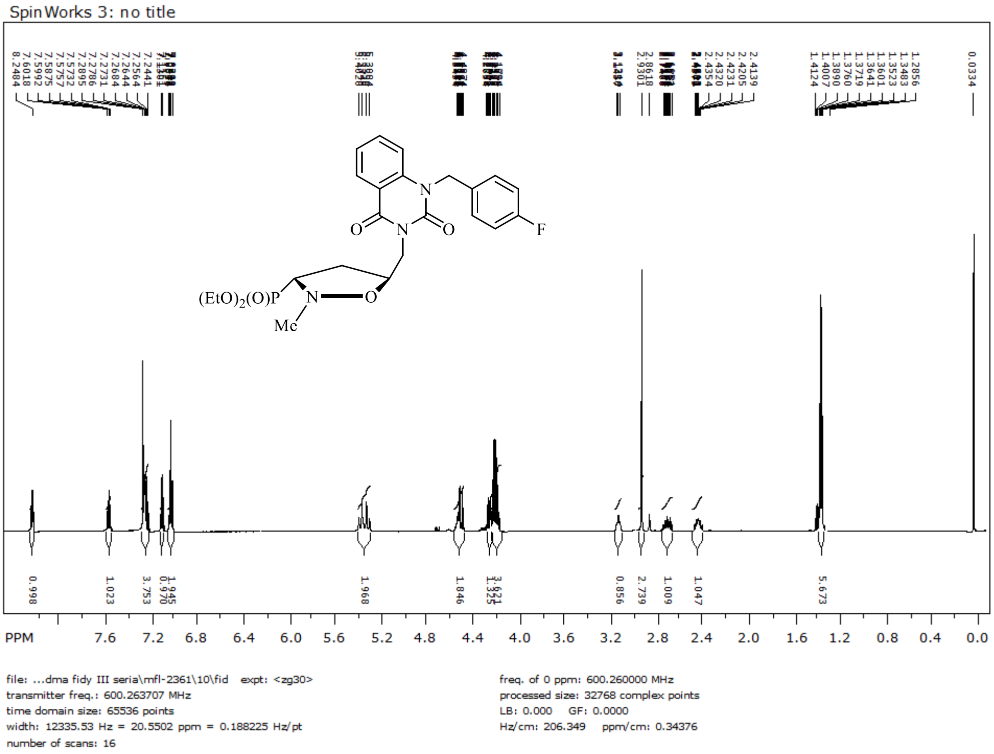

Figure S11: ^{31}P NMR Spectrum for *trans*-**20d** in $CDCl_3$

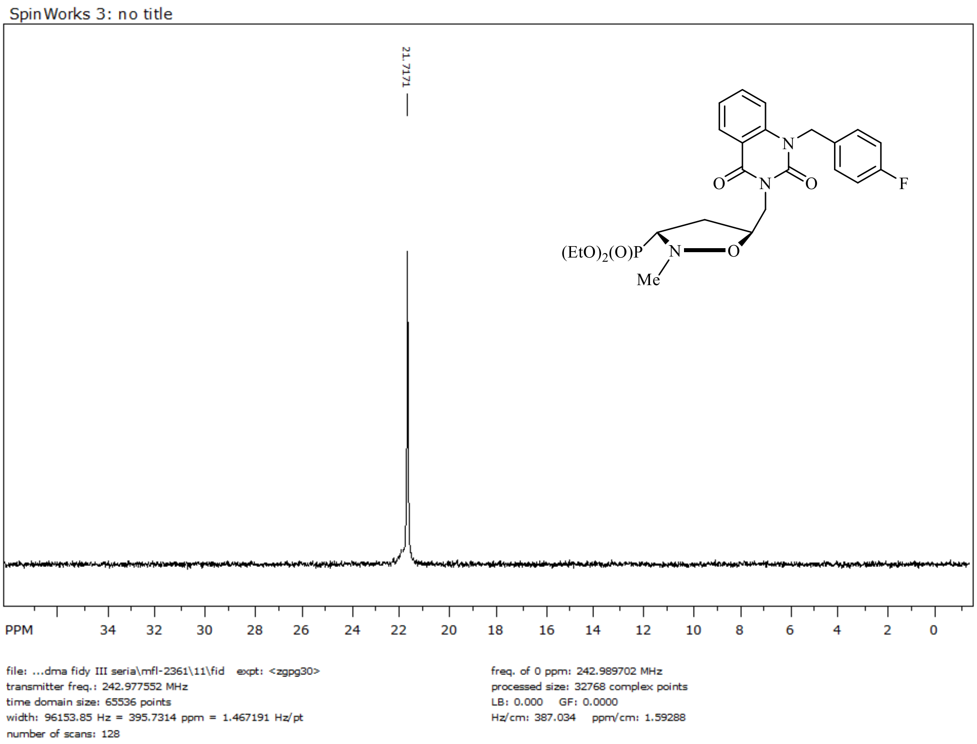

Figure S12: ^{13}C NMR Spectrum for *trans*-**20d** in $CDCl_3$

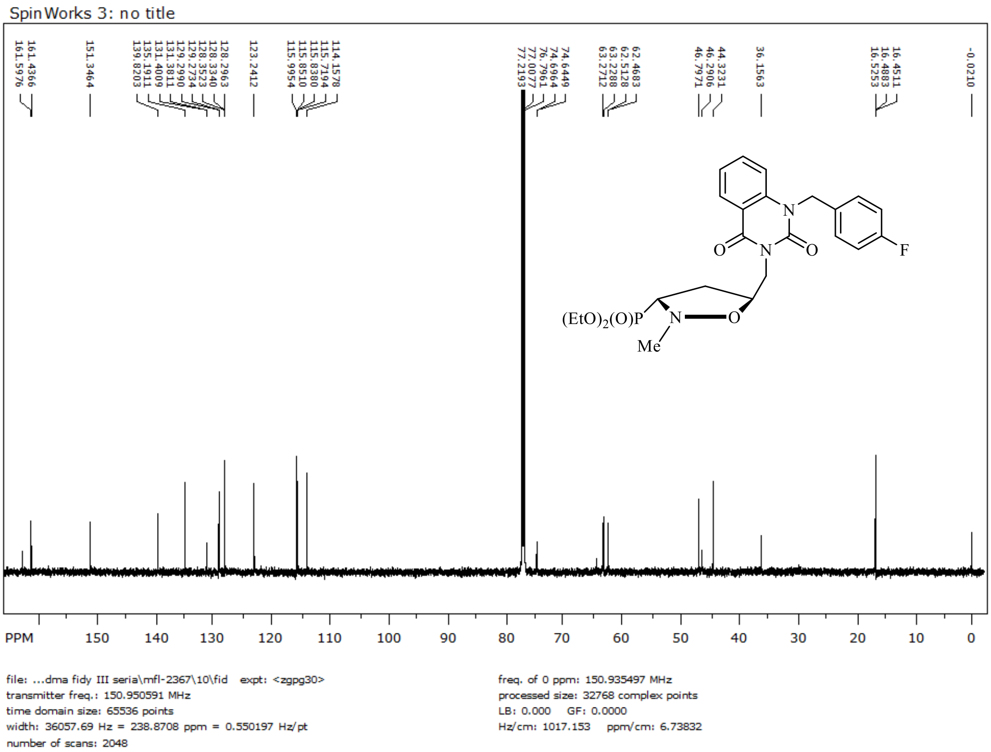

Figure S13: ^{1}H NMR Spectrum for *trans*-**21a** in $CDCl_3$

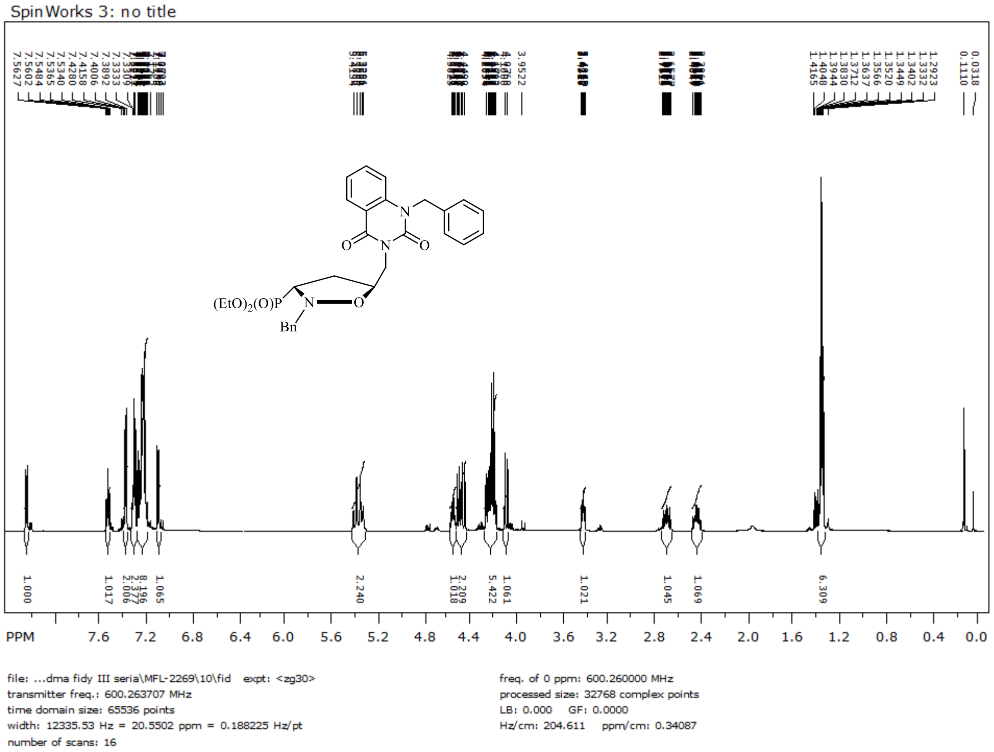

Figure S14: ^{31}P NMR Spectrum for *trans*-**21a** in $CDCl_3$

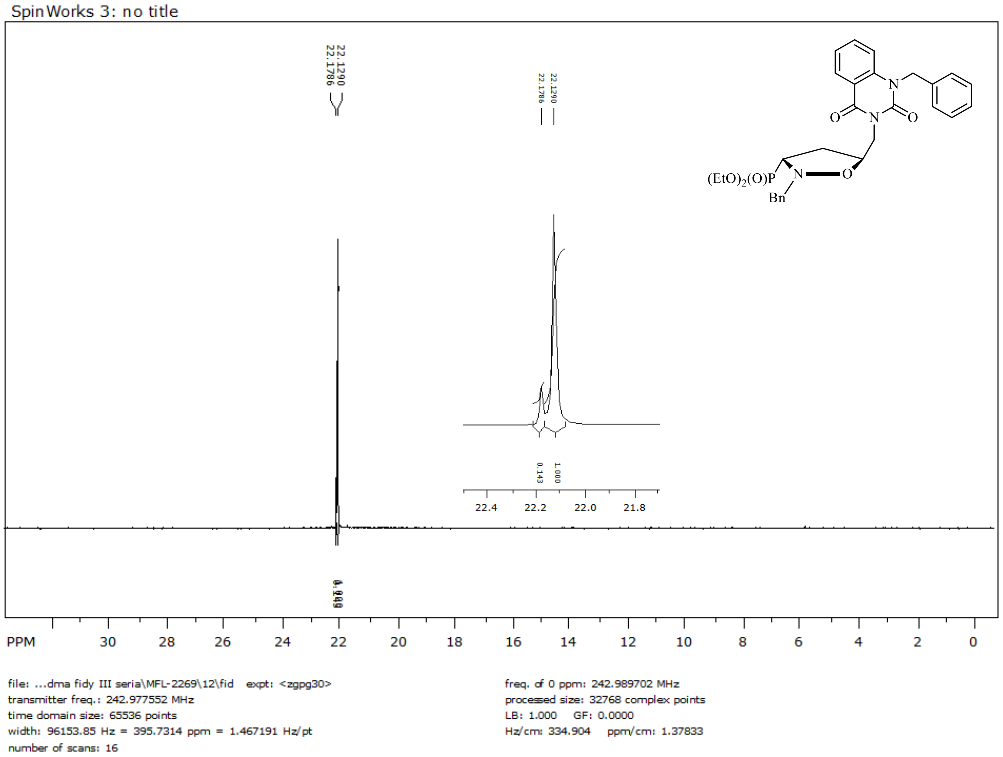

Figure S15: ^{13}C NMR Spectrum for *trans*-**21a** in $CDCl_3$

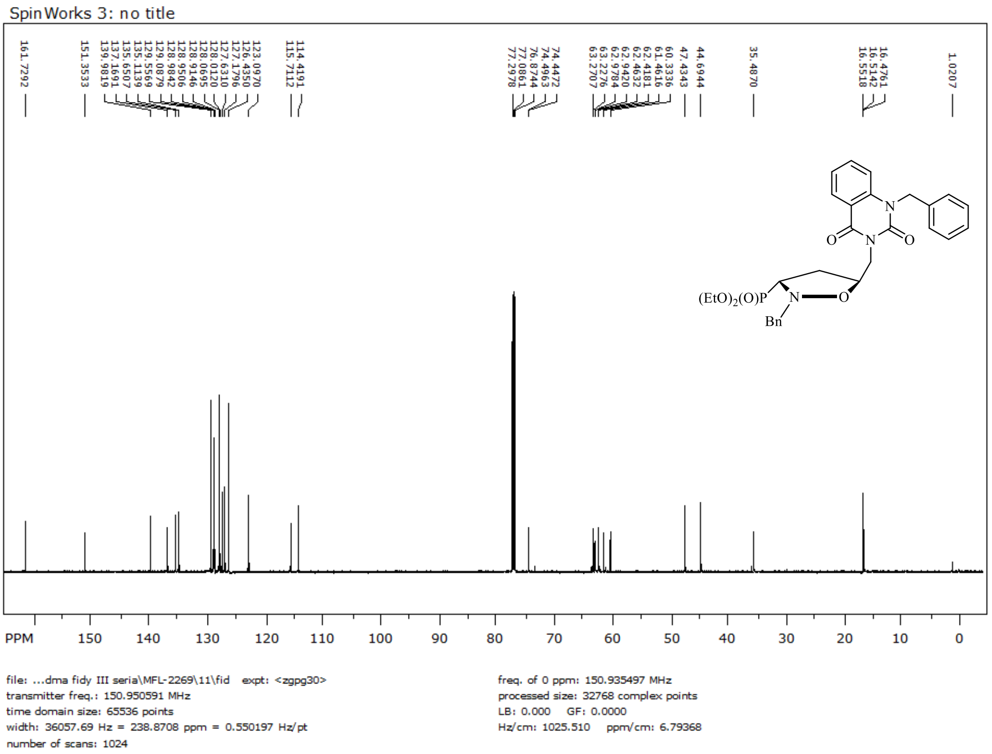

Figure S16: ^{1}H NMR Spectrum for *trans*-**21b** in $CDCl_3$

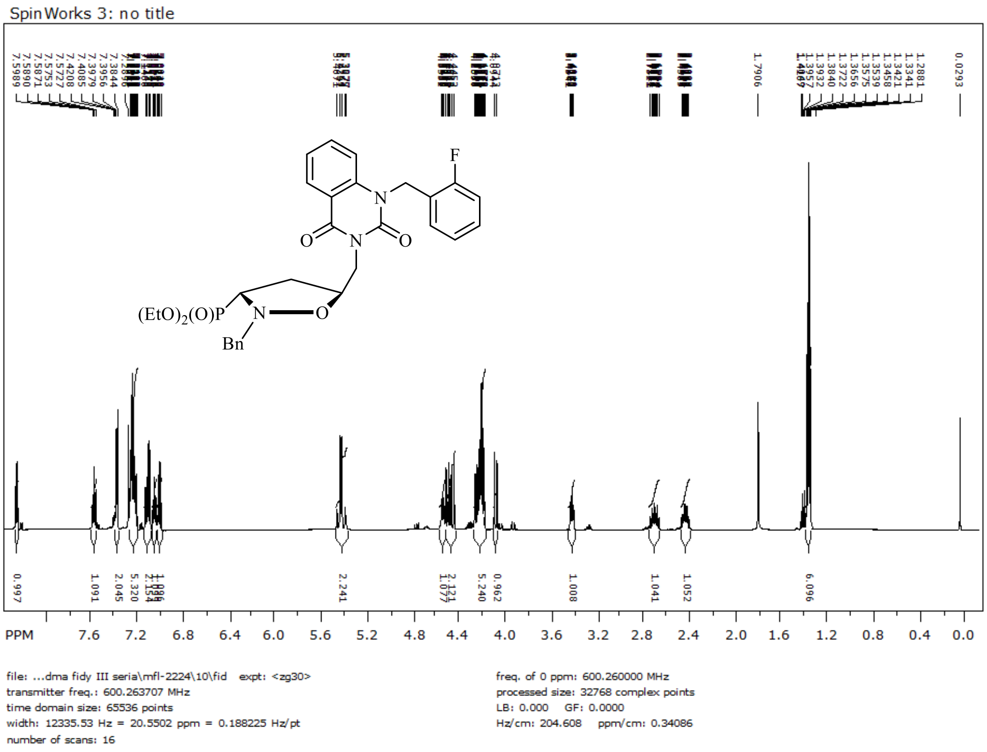

Figure S17: ^{31}P NMR Spectrum for *trans*-**21b** in $CDCl_3$

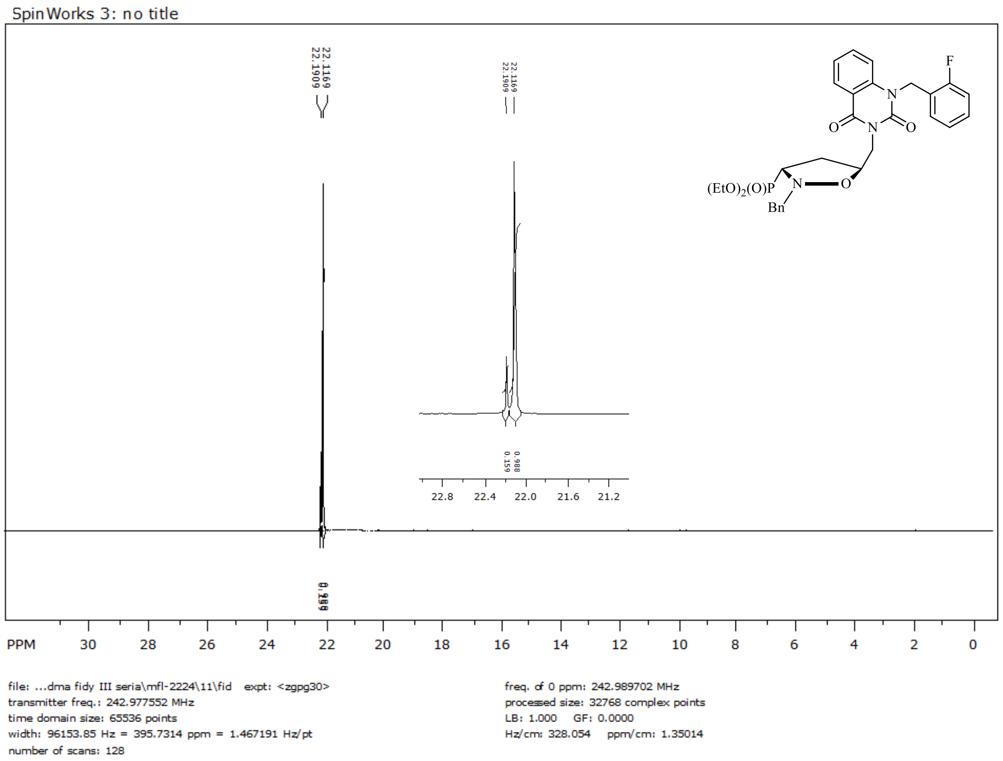

Figure S18: ^{13}C NMR Spectrum for *trans*-**21b** in $CDCl_3$

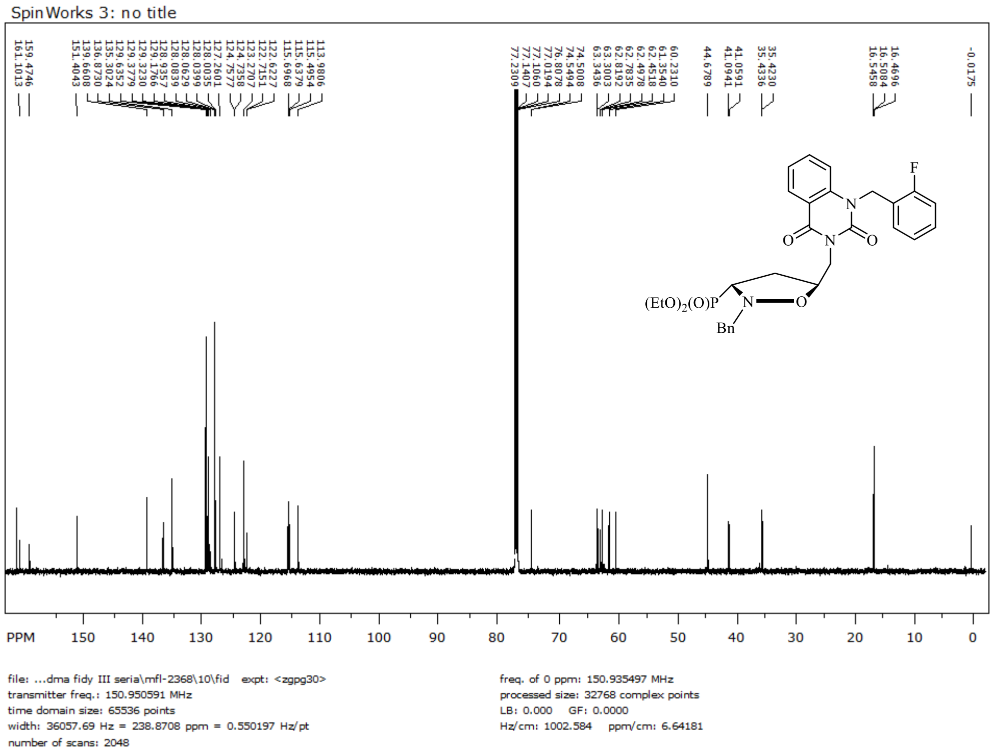

Figure S19: ^{1}H NMR Spectrum for *trans*-**21c** in $CDCl_3$

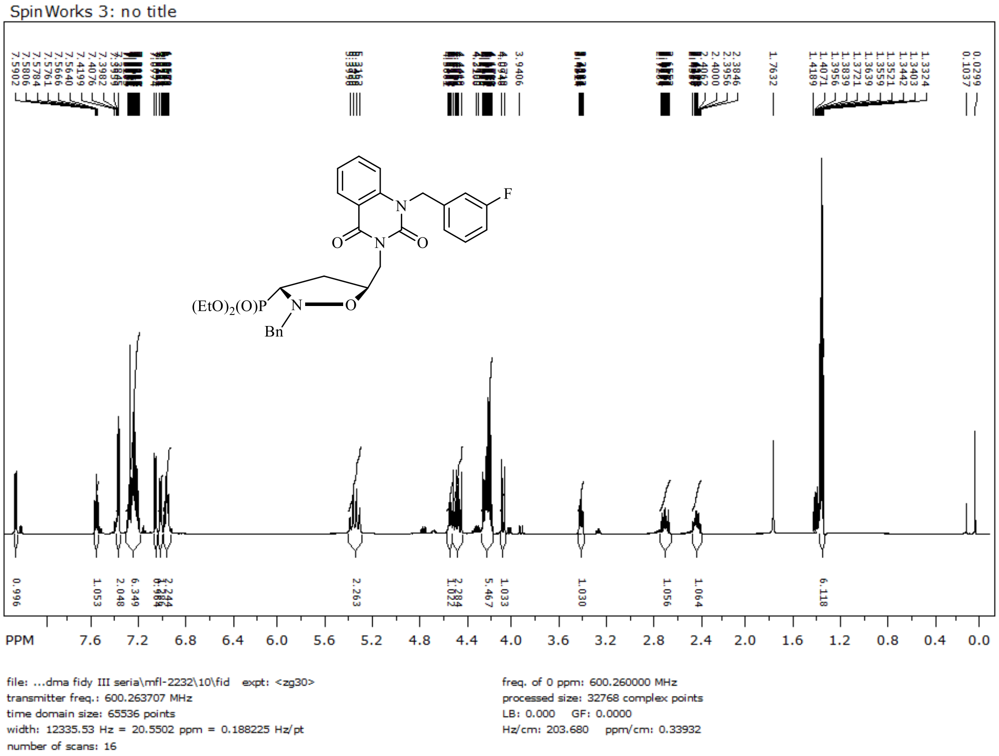

Figure S20: ^{31}P NMR Spectrum for *trans*-**21c** in $CDCl_3$

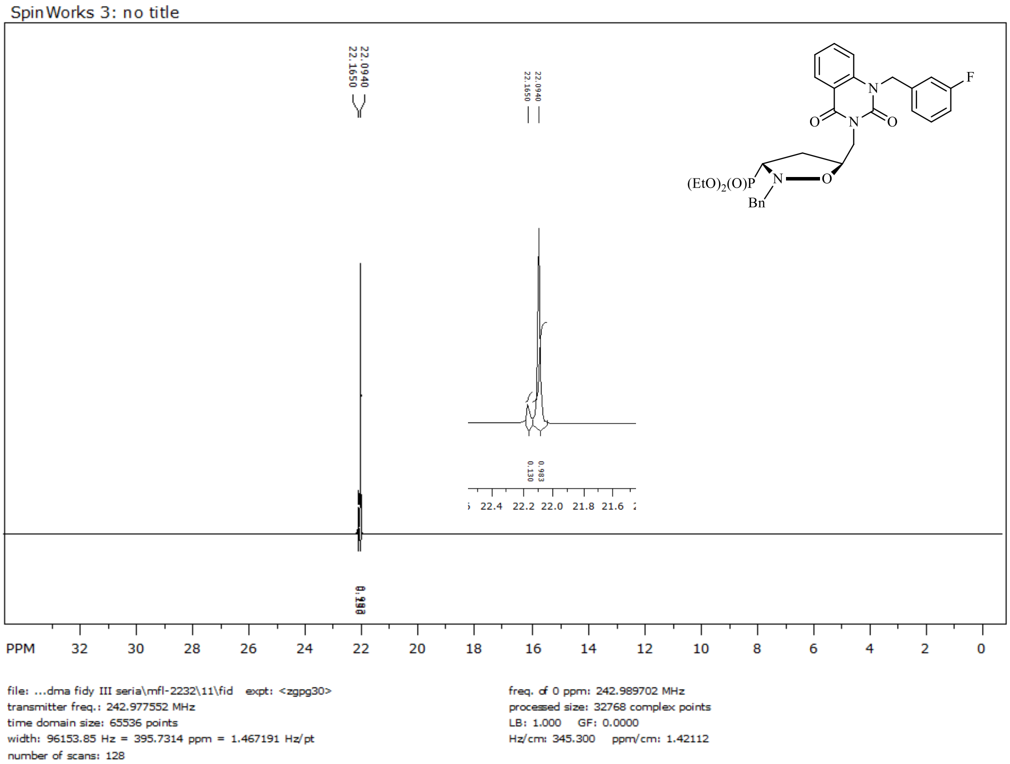

Figure S21: ^{13}C NMR Spectrum for *trans*-**21c** in $CDCl_3$

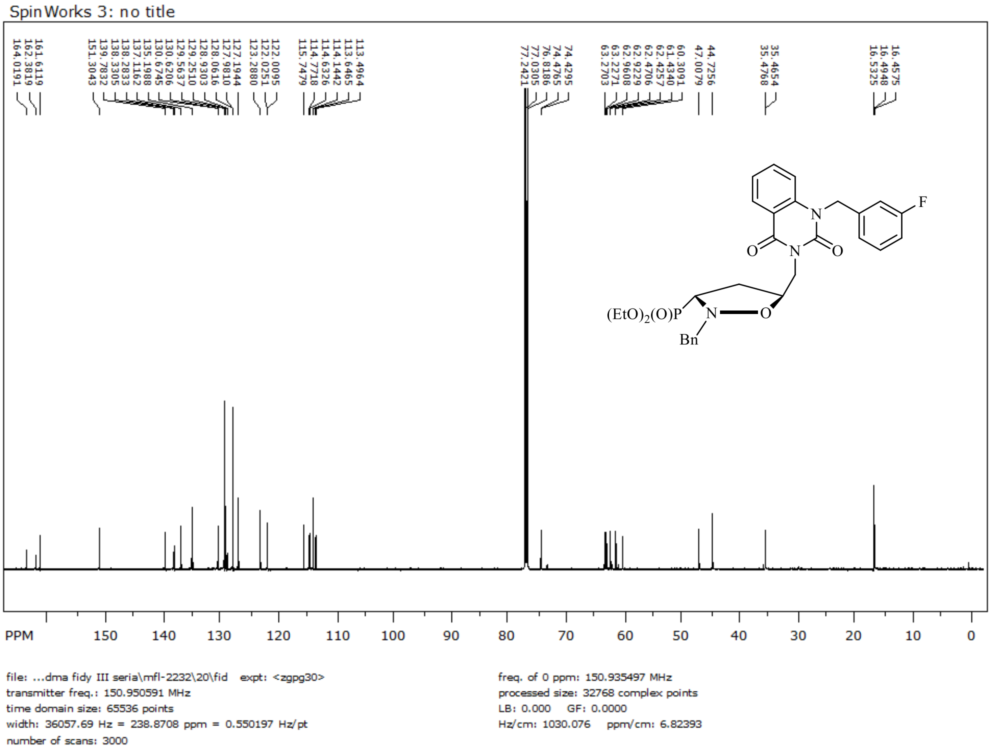

Figure S22: ^{1}H NMR Spectrum for *trans*-**21d** in $CDCl_3$

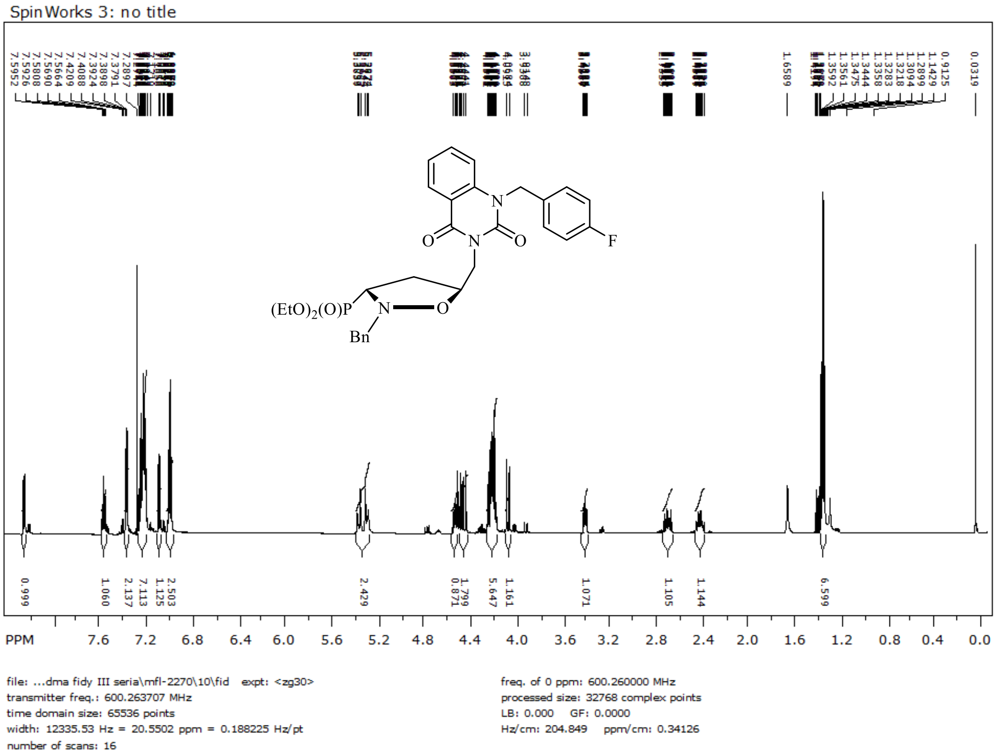

Figure S23: ^{31}P NMR Spectrum for *trans*-**21d** in $CDCl_3$

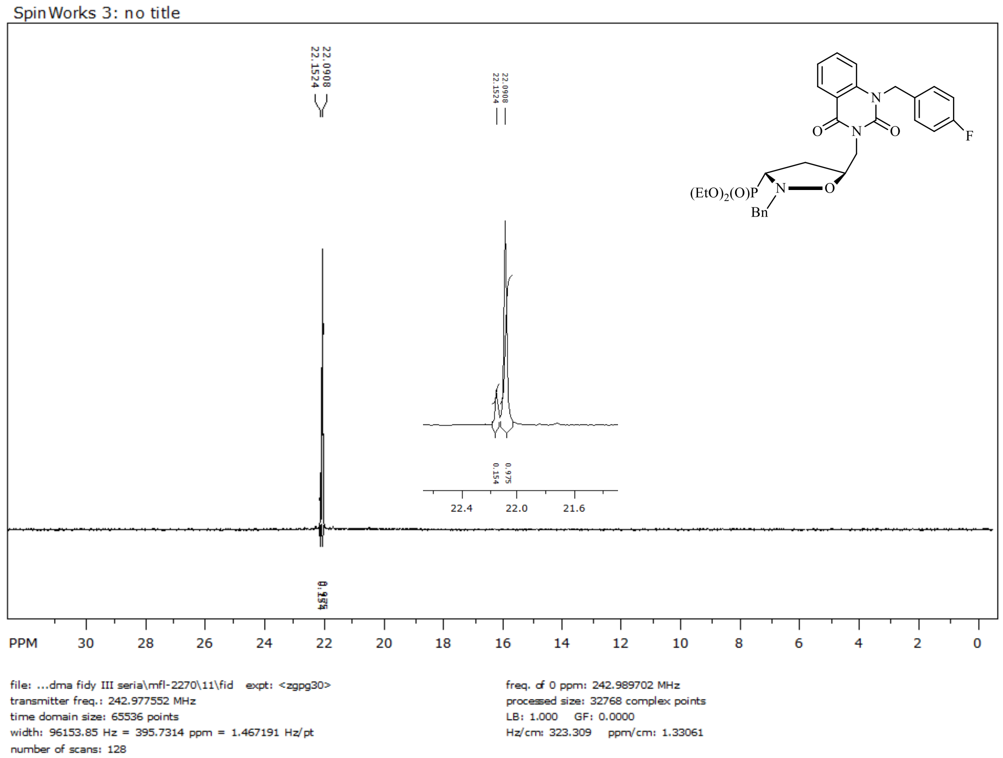

Figure S24: ^{13}C NMR Spectrum for *trans*-**21d** in $CDCl_3$

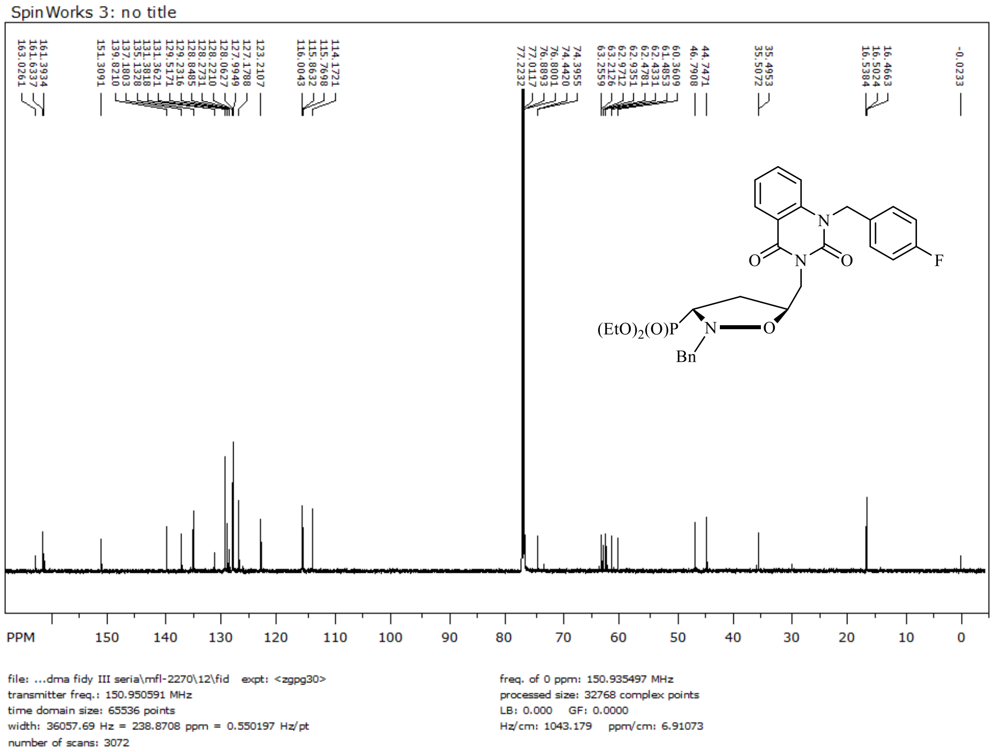

Figure S25: ^{1}H NMR Spectrum for **24a** in $CDCl_3$

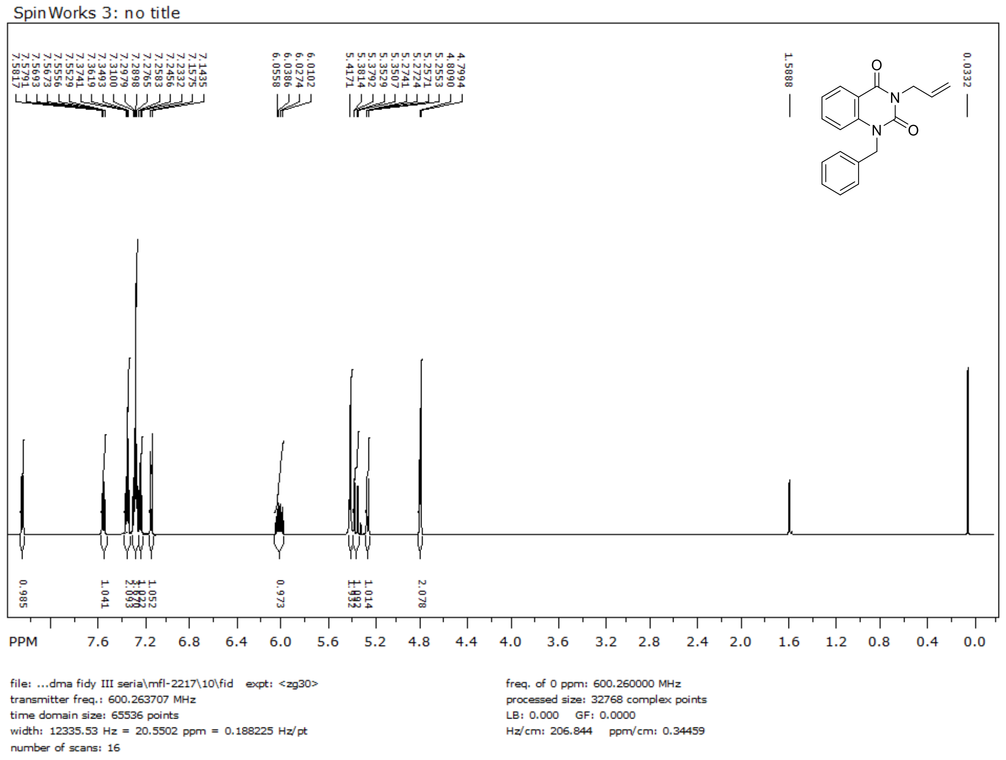

Figure S26: ^{13}C NMR Spectrum for **24a** in $CDCl_3$

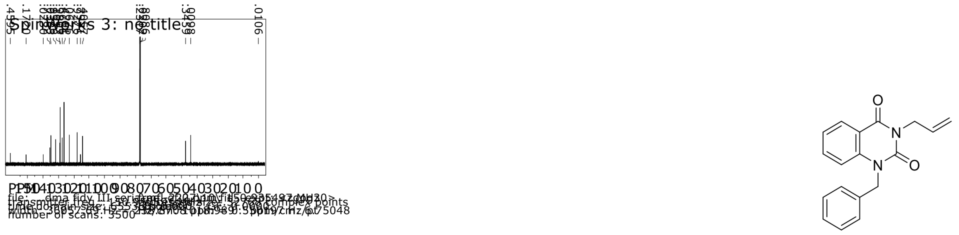

Figure S27: ^{1}H NMR Spectrum for **24b** in $CDCl_3$

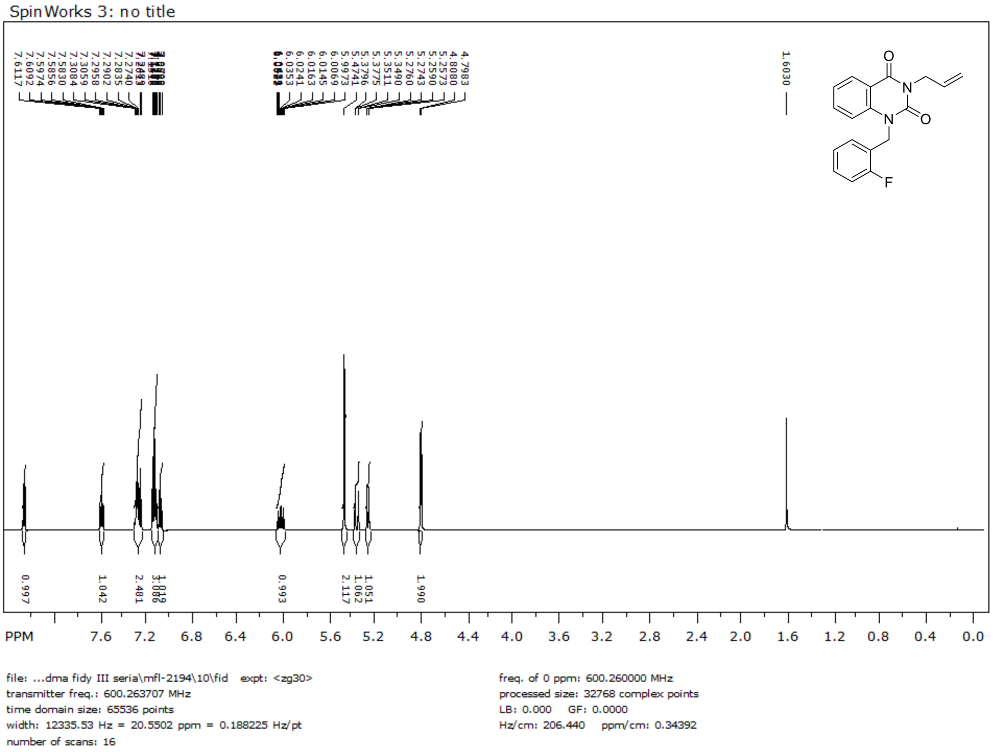

Figure S28: ^{13}C NMR Spectrum for **24b** in $CDCl_3$

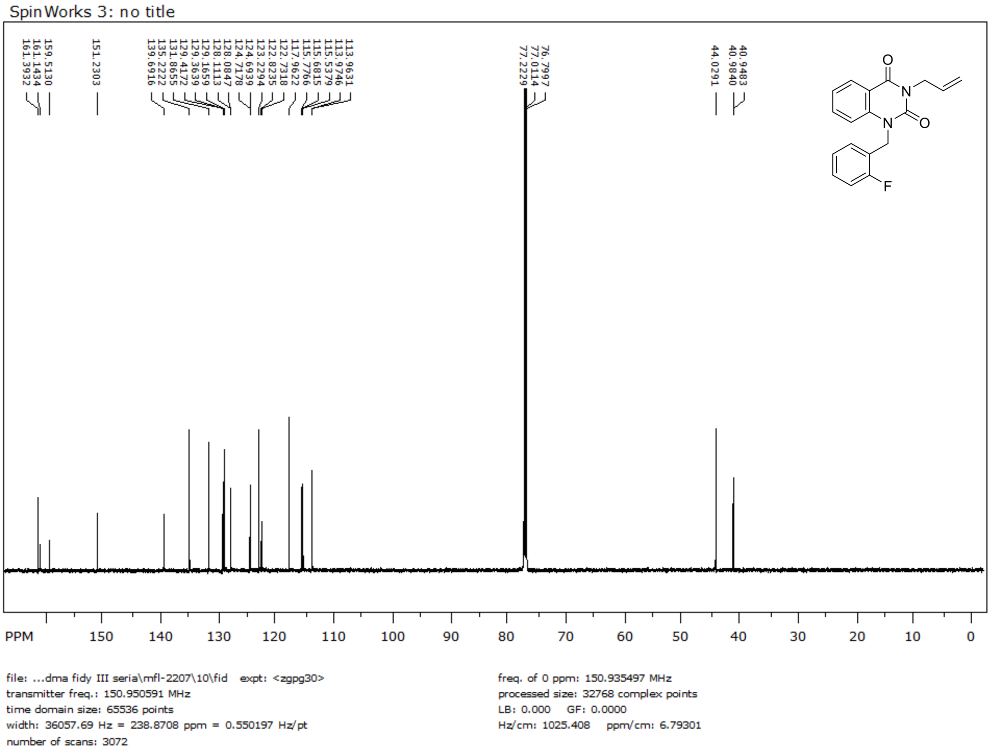

Figure S29: ^{1}H NMR Spectrum for **24c** in $CDCl_3$

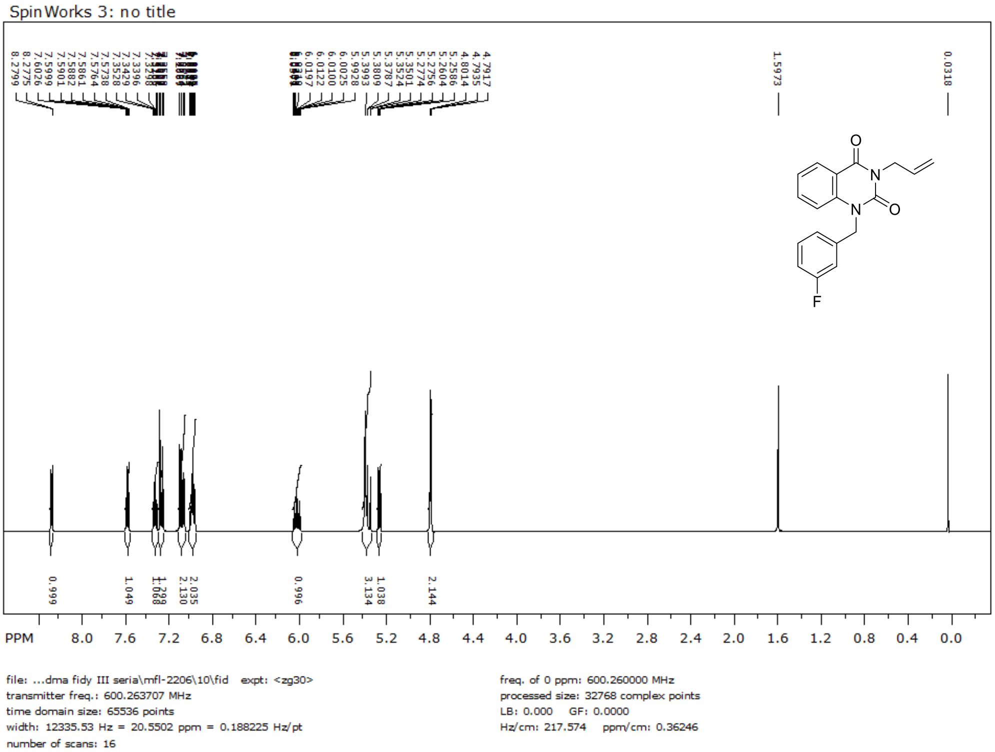

Figure S30: ^{13}C NMR Spectrum for **24c** in $CDCl_3$

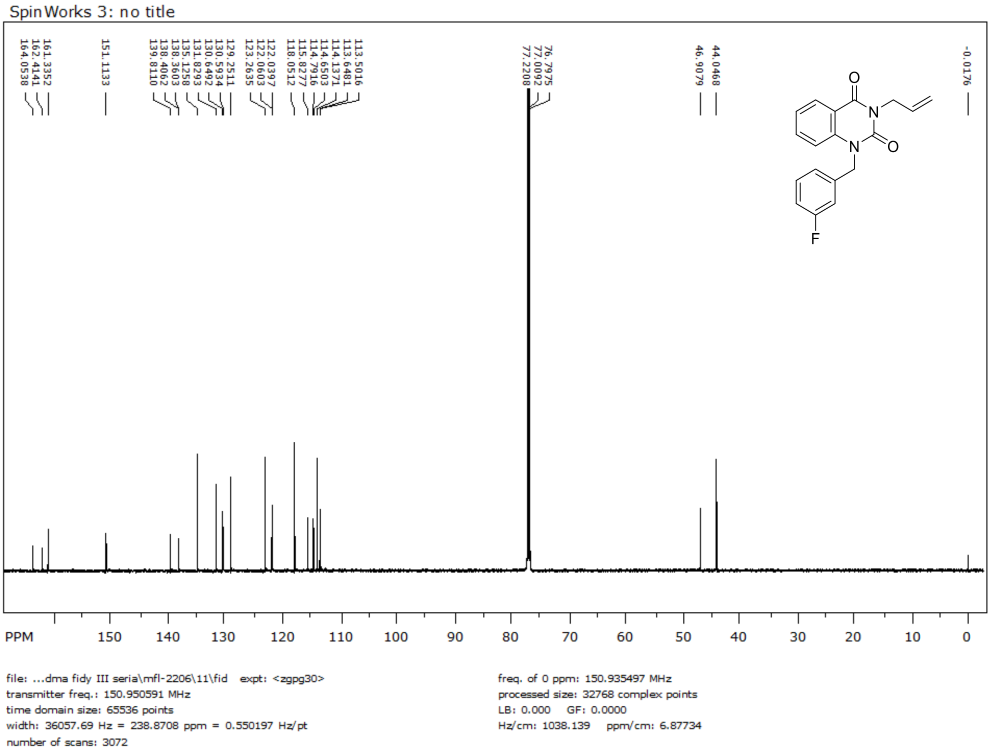

Figure S31: ^{1}H NMR Spectrum for **24d** in $CDCl_3$

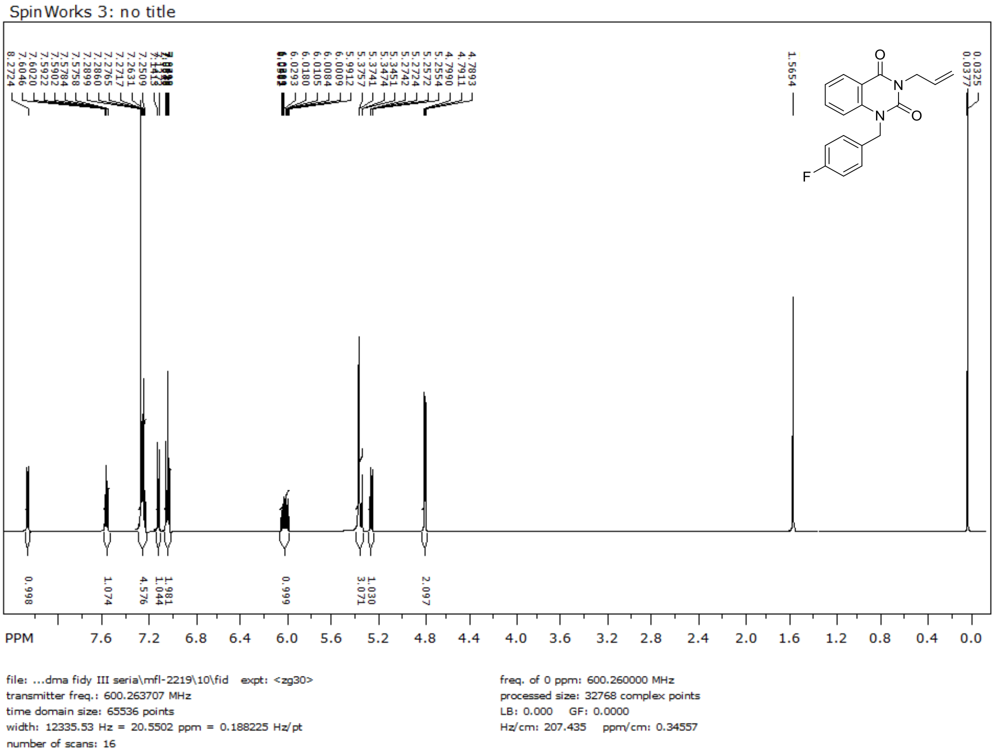

Figure S32: ^{13}C NMR Spectrum for **24d** in $CDCl_3$

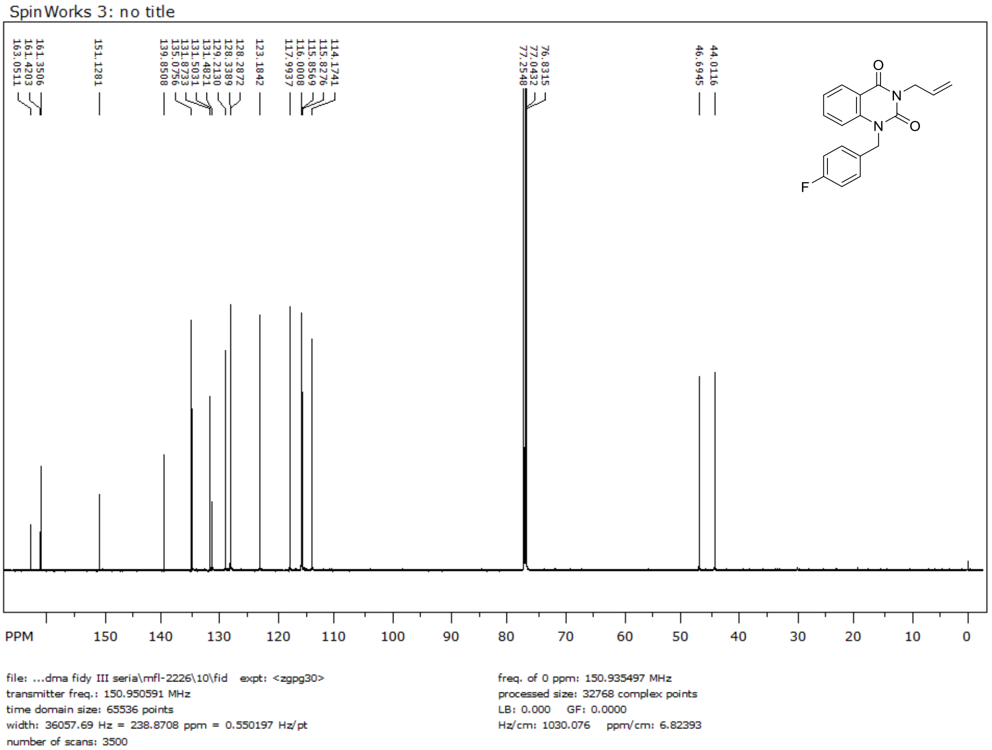

Figure S33: ^{1}H NMR Spectrum for **26a** in $CDCl_3$

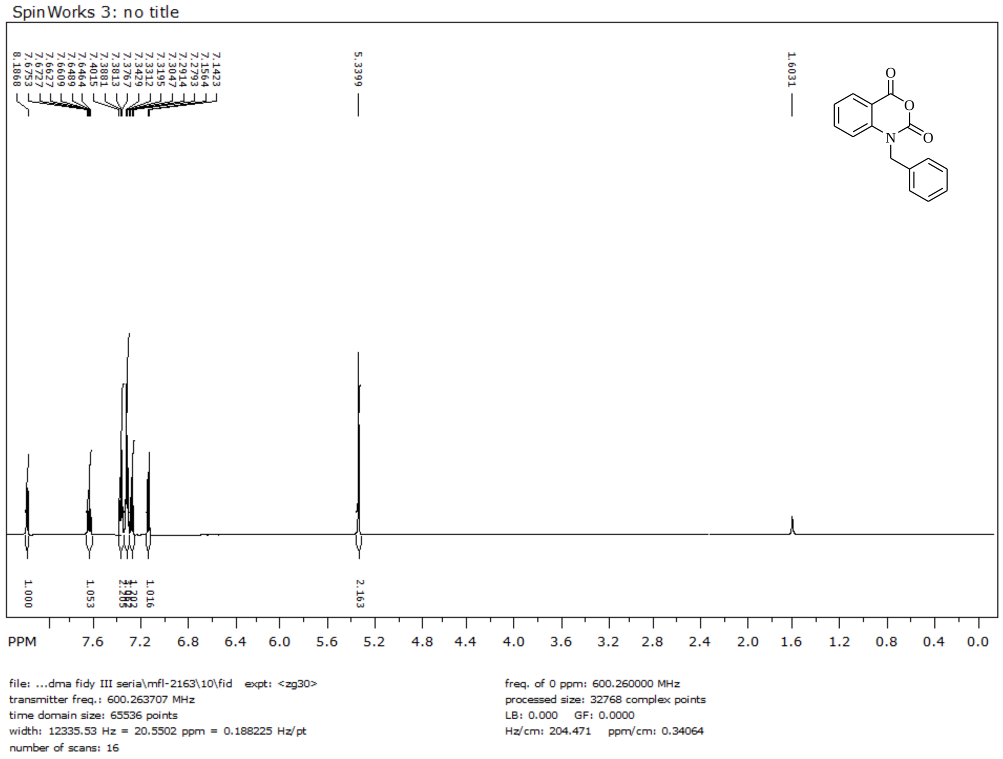

Figure S34: ^{13}C NMR Spectrum for **26a** in $CDCl_3$

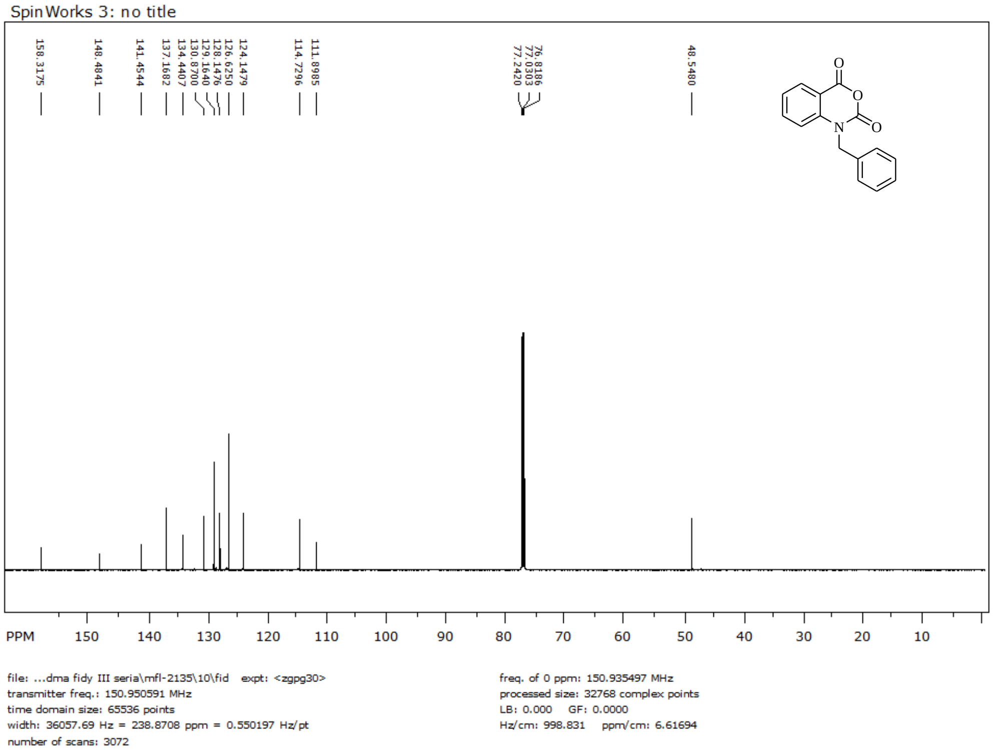

Figure S35: ^{1}H NMR Spectrum for **26b** in $CDCl_3$

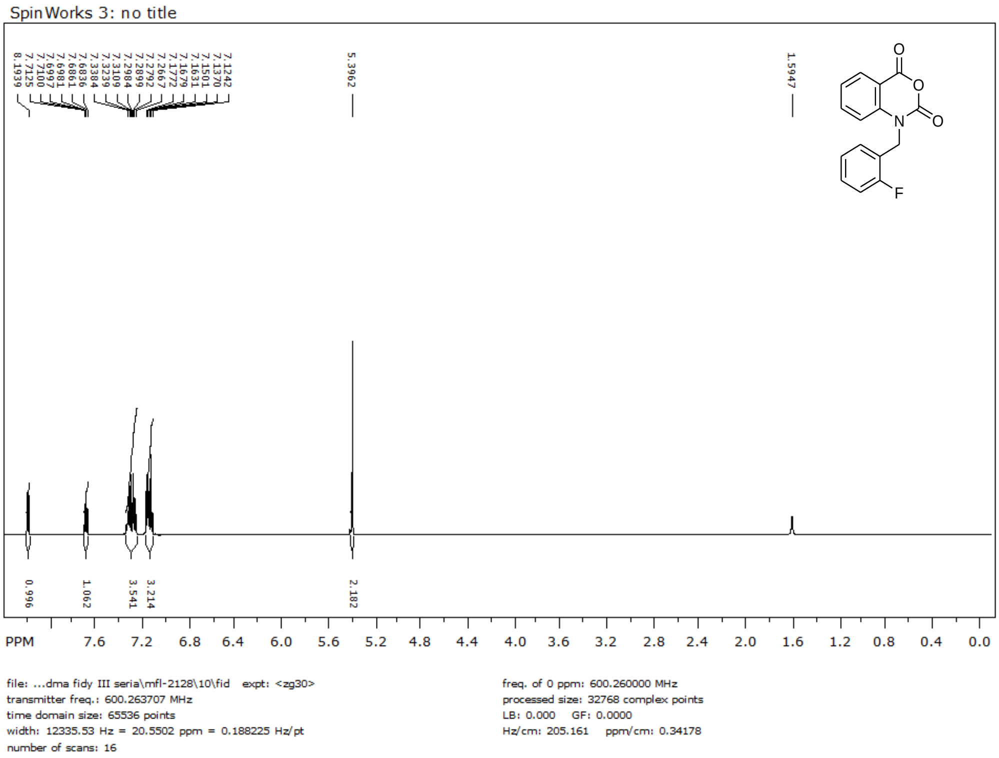

Figure S36: ^{13}C NMR Spectrum for **26b** in $CDCl_3$

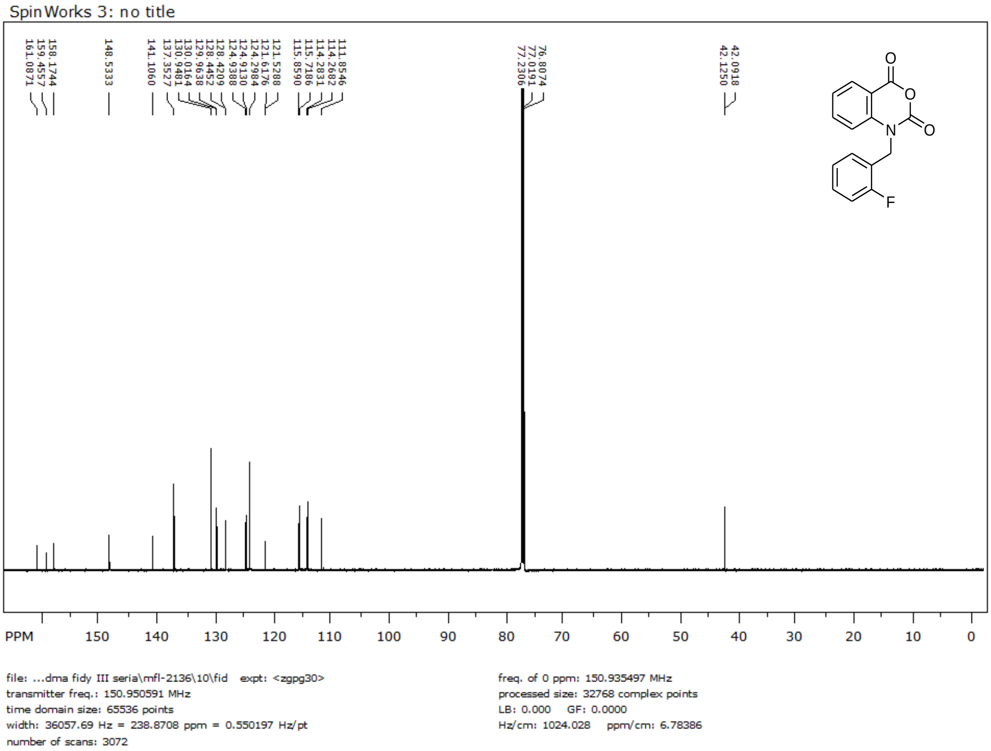

Figure S37: ^{1}H NMR Spectrum for **26c** in $CDCl_3$

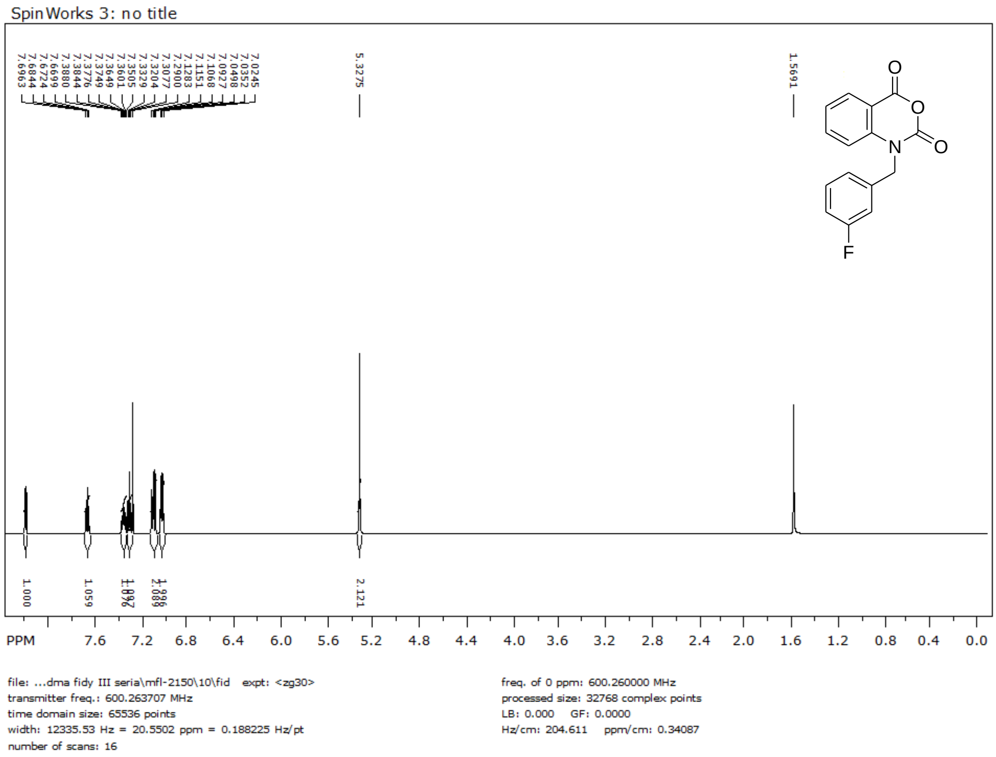

Figure S38: ^{13}C NMR Spectrum for **26c** in $CDCl_3$

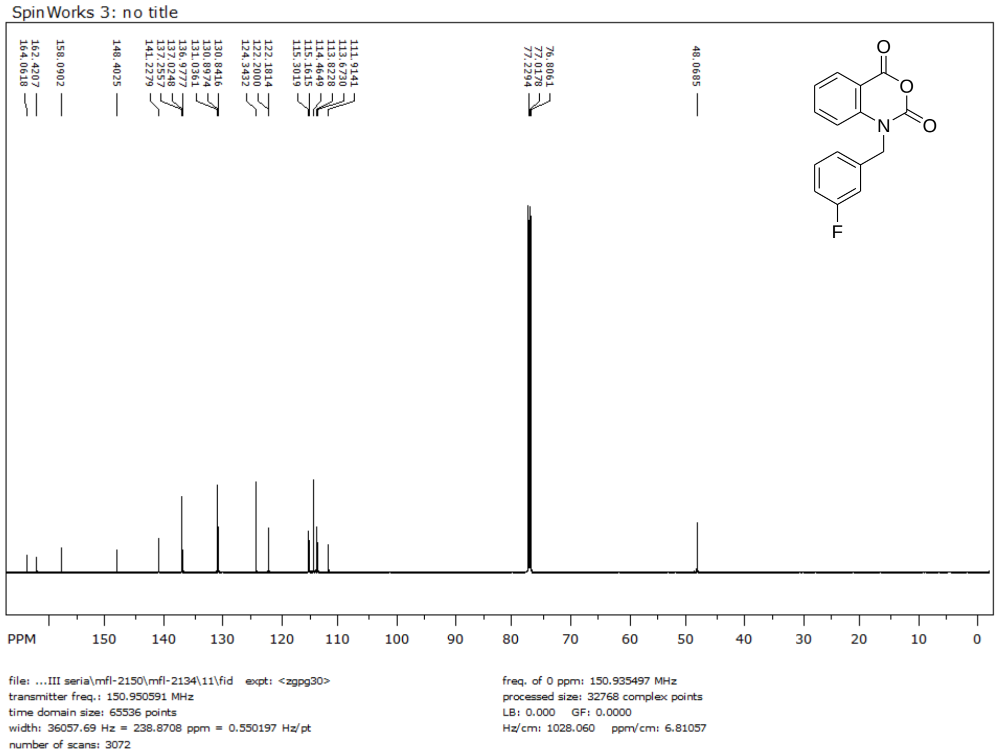

Figure S39: ^{1}H NMR Spectrum for **26d** in $CDCl_3$

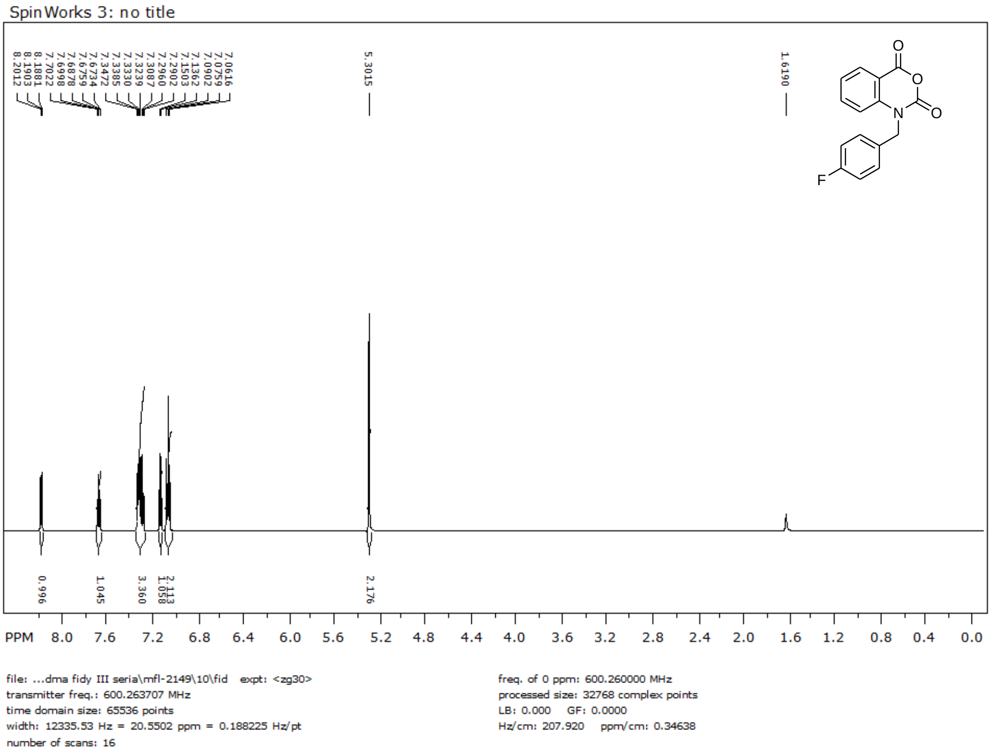

Figure S40: ^{13}C NMR Spectrum for **26d** in $CDCl_3$

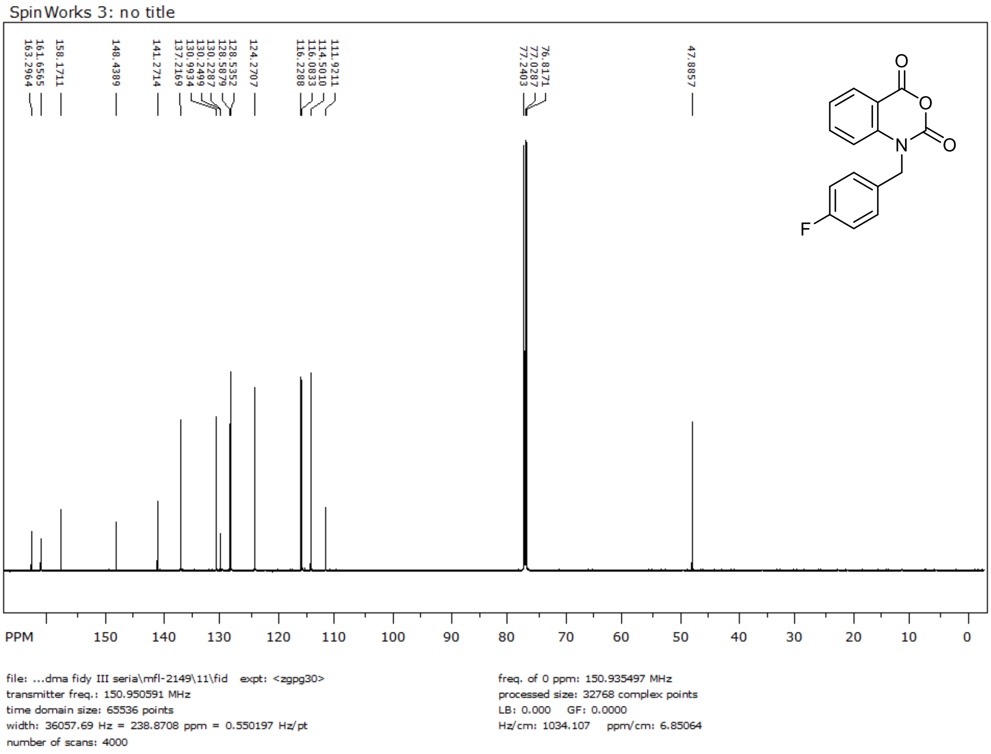

Figure S41: ^{1}H NMR Spectrum for **27a** in $CDCl_3$

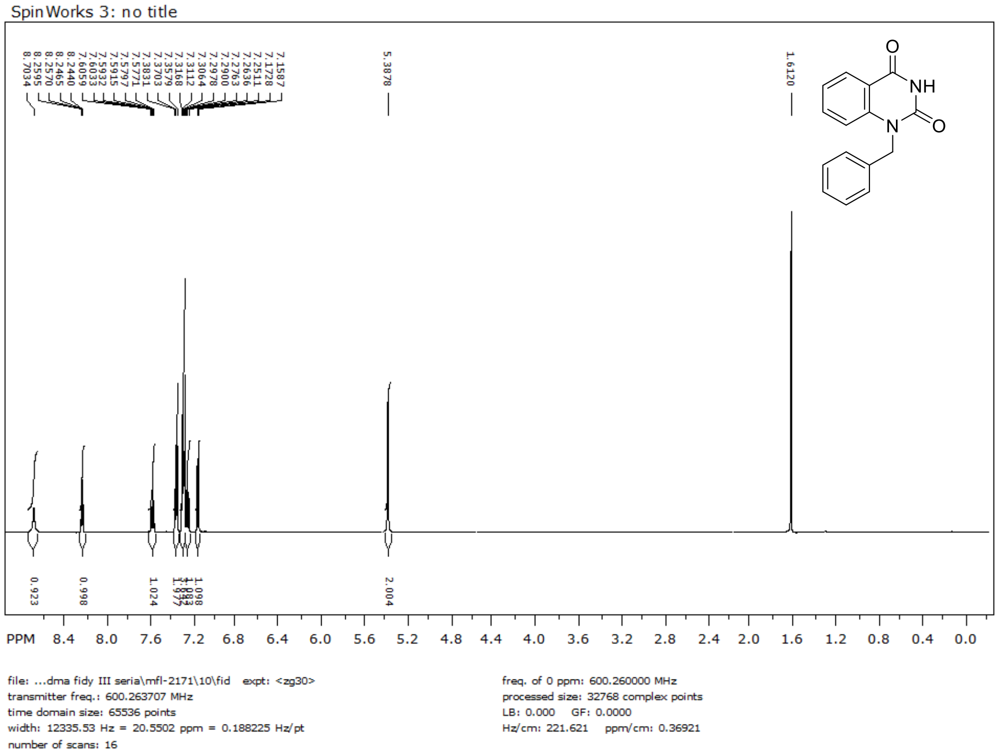

Figure S42: ^{13}C NMR Spectrum for **27a** in $CDCl_3$

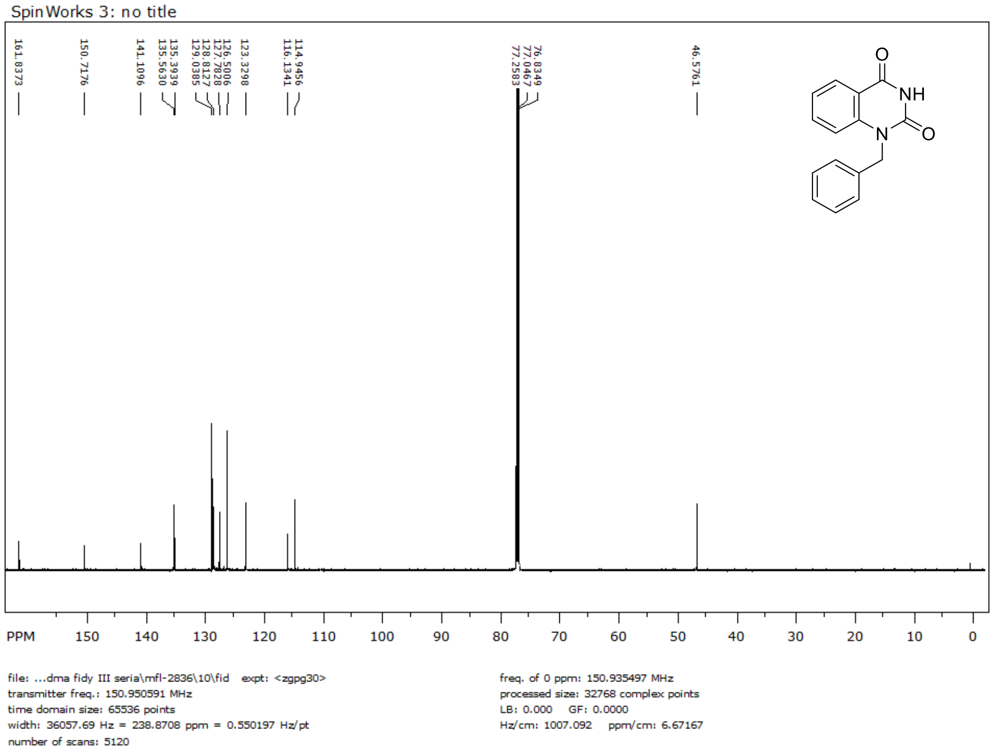

Figure S43: ^{1}H NMR Spectrum for **27b** in $CDCl_3$

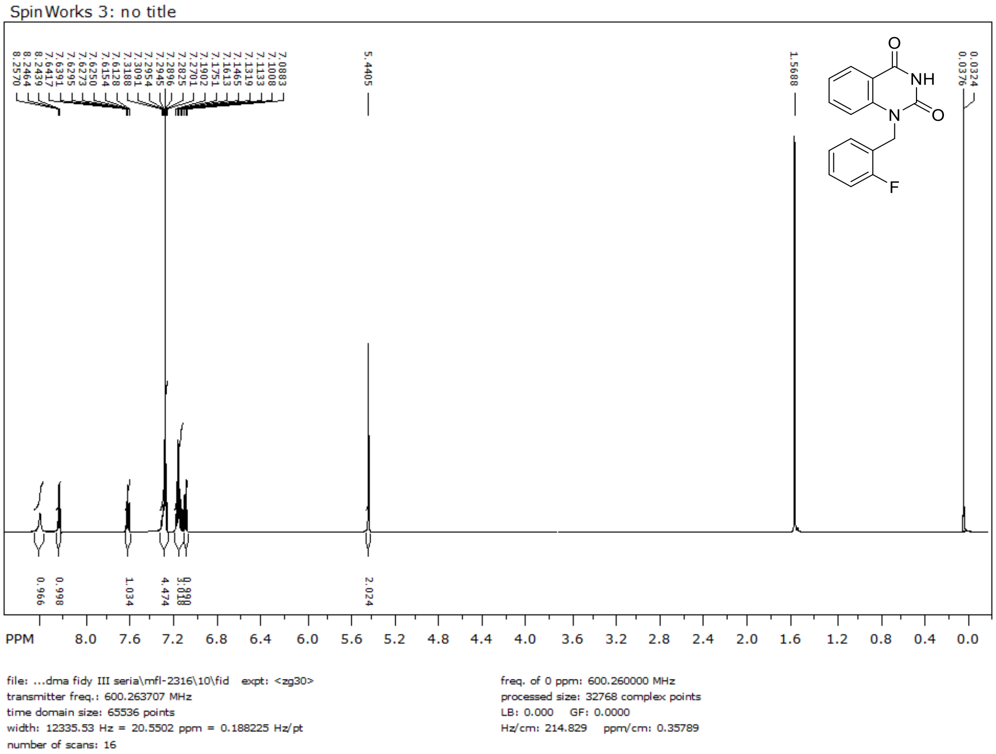

Figure S44: ^{13}C NMR Spectrum for **27b** in $CDCl_3$

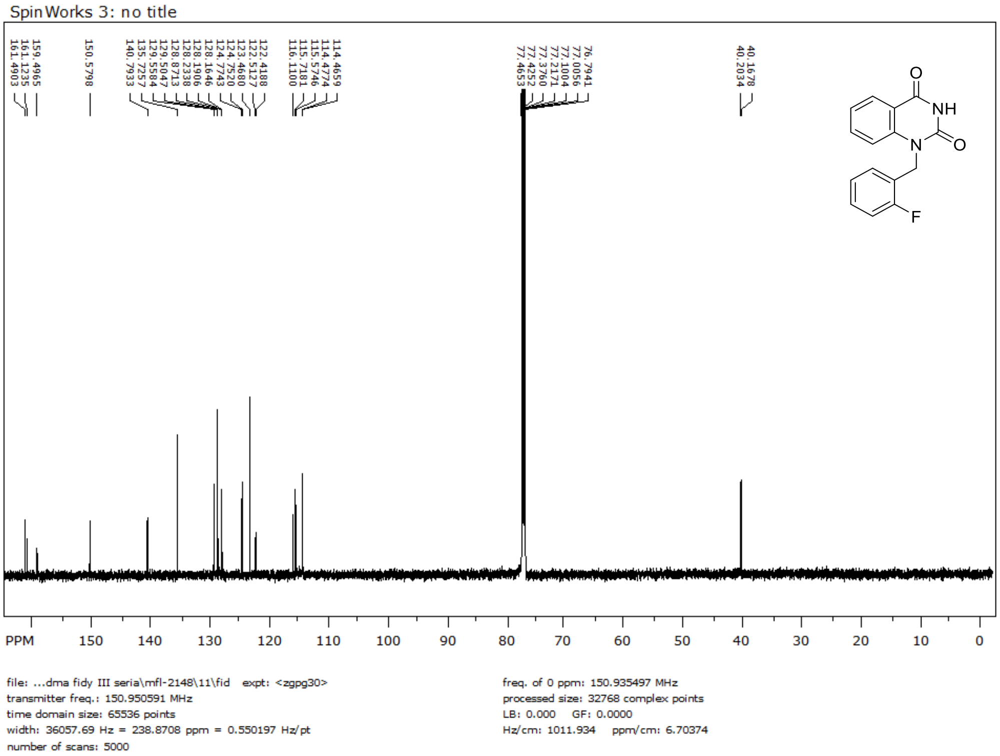

Figure S45: ^{1}H NMR Spectrum for **27c** in $CDCl_3$

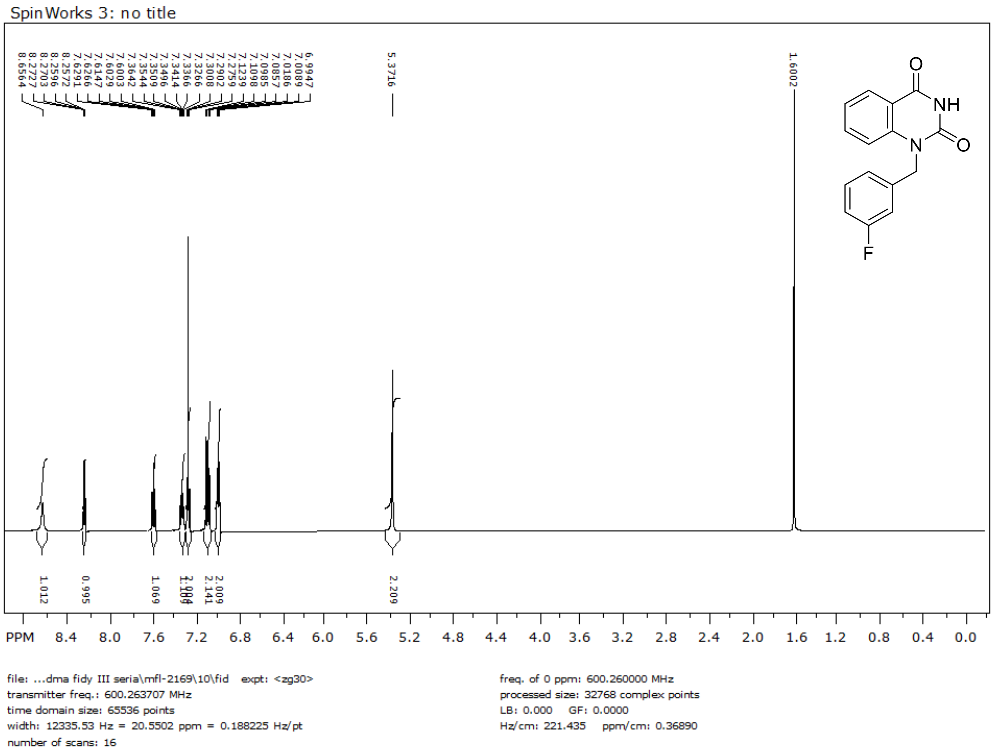

Figure S46: ^{13}C NMR Spectrum for **27c** in $CDCl_3$

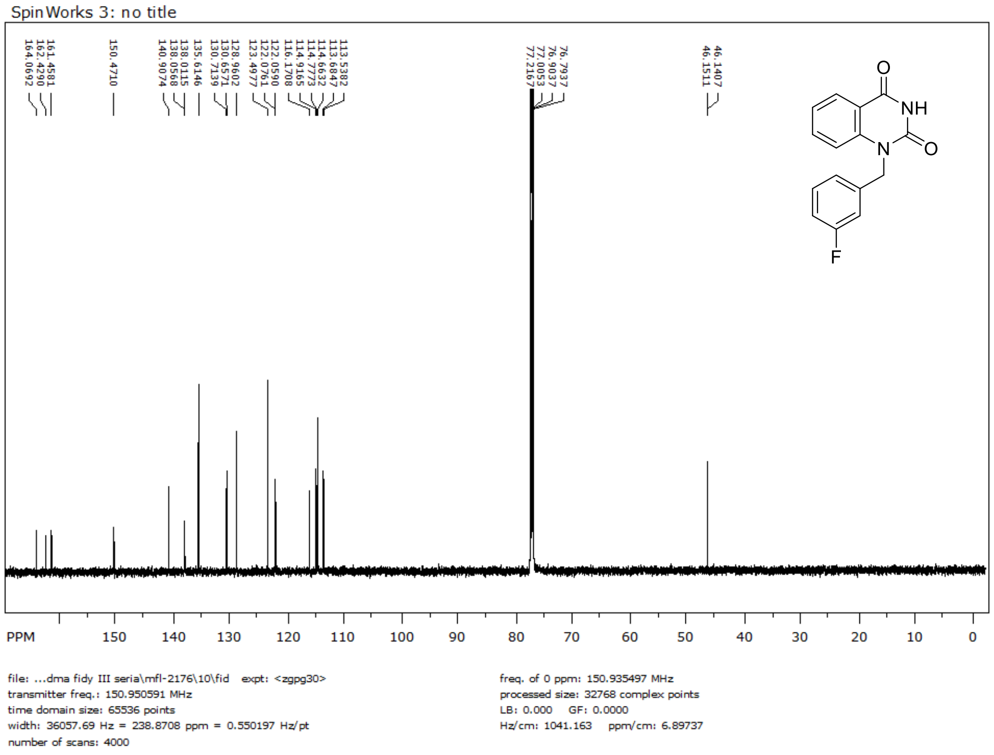

Figure S47: ^{1}H NMR Spectrum for **27d** in $CDCl_3$

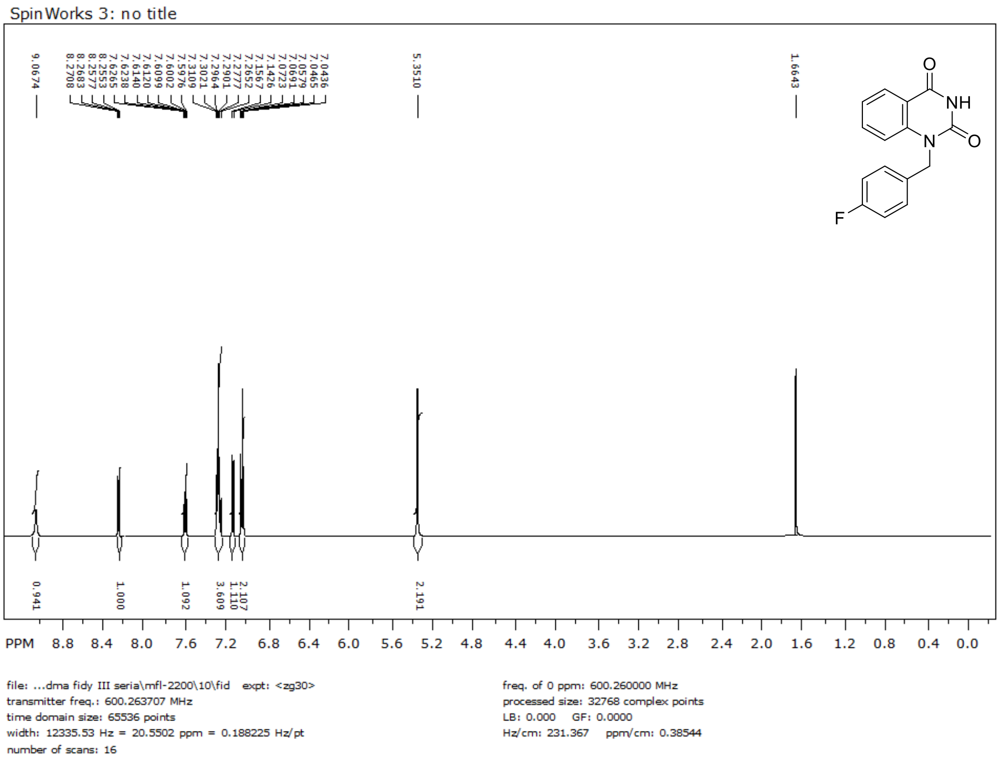

Figure S48. ^{13}C NMR Spectrum for **27d** in $CDCl_3$

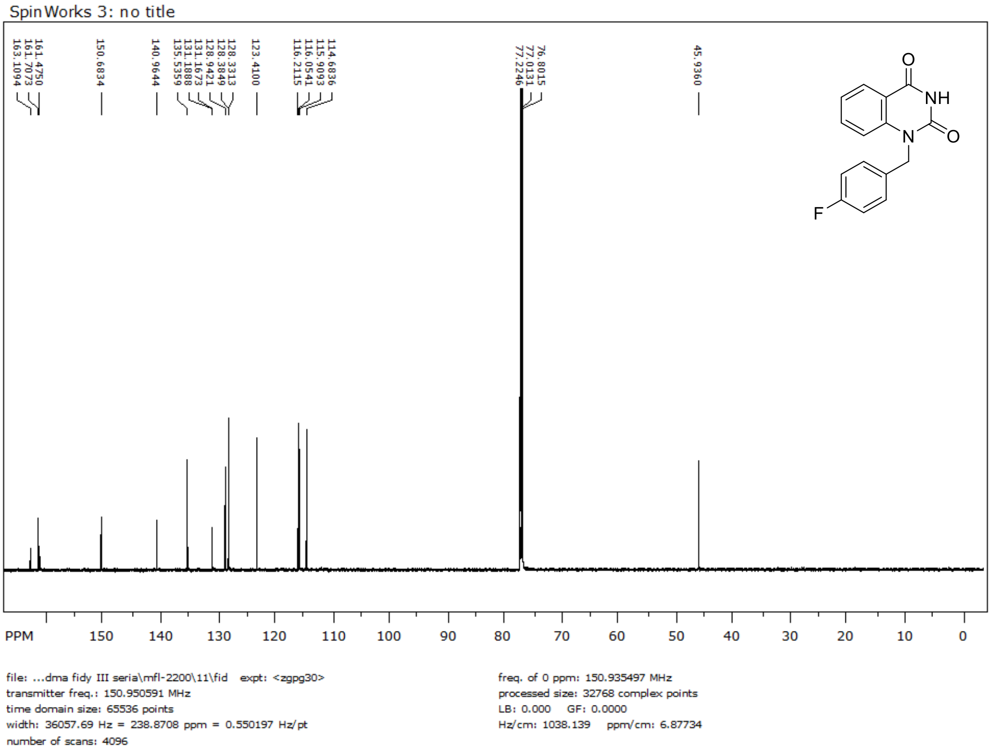

Supplement: Supplementary file 1 [file molecules-27-06526-s001.zip › molecules-1942962-SI.pdf]
